# Supplementary material for: Distinct pathogenic mutations in ARF1 allow dissection of its dual role in cGAS-STING signalling
Source: EMBO Rep. 2025 Mar 24;26(9):2232–61. doi: 10.1038/s44319-025-00423-7 (PMC7617634; doi:10.1038/s44319-025-00423-7)
Supplement: Supplementary file 1 — Appendix [file 44319_2025_423_MOESM1_ESM.pdf]

Appendix

Distinct pathogenic mutations in ARF1 allow dissection of its dual role in cGAS-STING signalling

Johannes Lang[1], Tim Bergner[2], Julia Zinngrebe[3], Alice Lepelley[4], Katharina Vill[5], Steffen Leiz[6], Meinhard Wlaschek[7], Matias Wagner[8,9], Karin Scharffetter-Kochanek[7], Pamela Fischer-Posovszky[3,10], Clarissa Read[2], Yanick J Crow[4,11], Maximilian Hirschenberger[1,*] and Konstantin MJ Sparrer[1,12,*]

*Address correspondence to:
Konstantin MJ Sparrer: Konstantin.Sparrer@uni-ulm.de, Maximilian Hirschenberger: maximilian.hirschenberger@uni-ulm.de

Supplementary patient description

Supplementary patient description

AGS3238

This male was born to non-consanguineous parents (parental age at birth: 32 years/32 years) following a complication-free pregnancy with average birth weight and size. He has two older, healthy brothers. At age 18 months he was not yet walking or articulating. Neurodevelopmental examination indicated delays in motor skills and speech, along with difficulties in oral-motor coordination that manifested as drooling and problems with swallowing. Cerebral magnetic resonance imaging (MRI) revealed periventricular nodular heterotopia (PVNH). Further examination identified hypotonia, Sprengel's deformity and joint hypermobility. No skin lesions were ever observed. Assessment of the expression of 24 interferon stimulated genes in blood using NanoString at at 20 months of age showed an elevated score (7.615, cut-off: 2.758, Fig. 1A). Overall, his condition has been stable, making some developmental progress. He was found to harbour a de novo heterozygous p.(Arg19Cys) mutation in ARF1.

The patient was most recently assessed during a developmental follow-up and extended communication consultation in January 2024:

Developmental and neurological status

The patient presents with generalised developmental delay (ICD-10: F89) with significant challenges in motor and communication:

- Motor development:

o Mobilises using a rear-facing walker and occasionally by scooting on the floor.

o Demonstrates improved transitions (e.g. sitting to standing) but lacks adequate arm support for falls.

o Fine motor skills include stacking blocks and assembling train tracks, although variability of play is limited.

- Communication:

o Receptive language: Understands simple words and two-element sentences (e.g. "the big cup") but struggles with more complex directions.

o Expressive language: Produces about 20 words or protowords with a limited consonantal repertoire, accompanied by dysarthric features and poor syllable diversity.

- Cognitive and play behaviour:

o Limited variability in play with emerging symbolic play elements.

o Shows good attention to familiar tasks, although overall cognitive skills remain underdeveloped.

Physical examination

- Anthropometry: Height and weight below age-specific norms; head circumference in the 94th percentile.

- Muscle tone: Generalised hypotonia without significant strength impairment.

- Craniofacial and neurological examination: MRI revealed subependymal heterotopias with thin corpus callosum; no gross structural abnormalities or cerebellar atrophy.

Additional findings

- Growth: Decreased IGF-1 levels; continued monitoring recommended.

- Nutrition: Persistent caloric deficit (~200 kcal/day) with limited acceptance of nutritional supplements.

- Gastrointestinal: Constipation treated with Laxbene.

- Orthopaedics: Mild acetabular dysplasia without subluxation; no indication for surgical intervention.
