# Supplementary figures and images for: Distinct pathogenic mutations in ARF1 allow dissection of its dual role in cGAS-STING signalling (part 1 of 2)
Source: EMBO Rep. 2025 Mar 24;26(9):2232–61. doi: 10.1038/s44319-025-00423-7 (PMC7617634; doi:10.1038/s44319-025-00423-7)

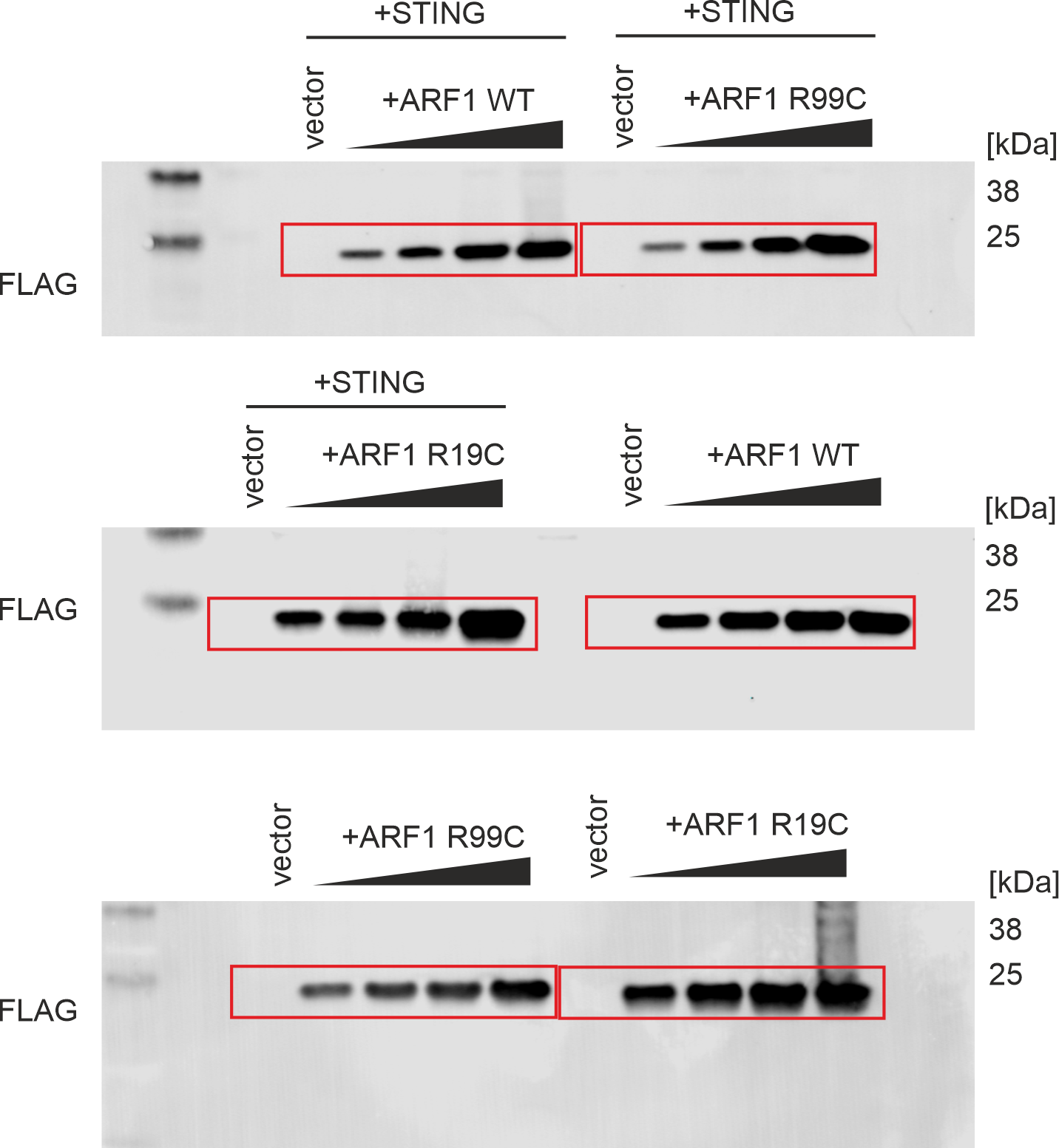

Supplement: Supplementary file 3 — Source data Fig. 1 [file 44319_2025_423_MOESM3_ESM.zip › Figure 1/1C/Western blots FLAG WT, R19C, R99C.png]

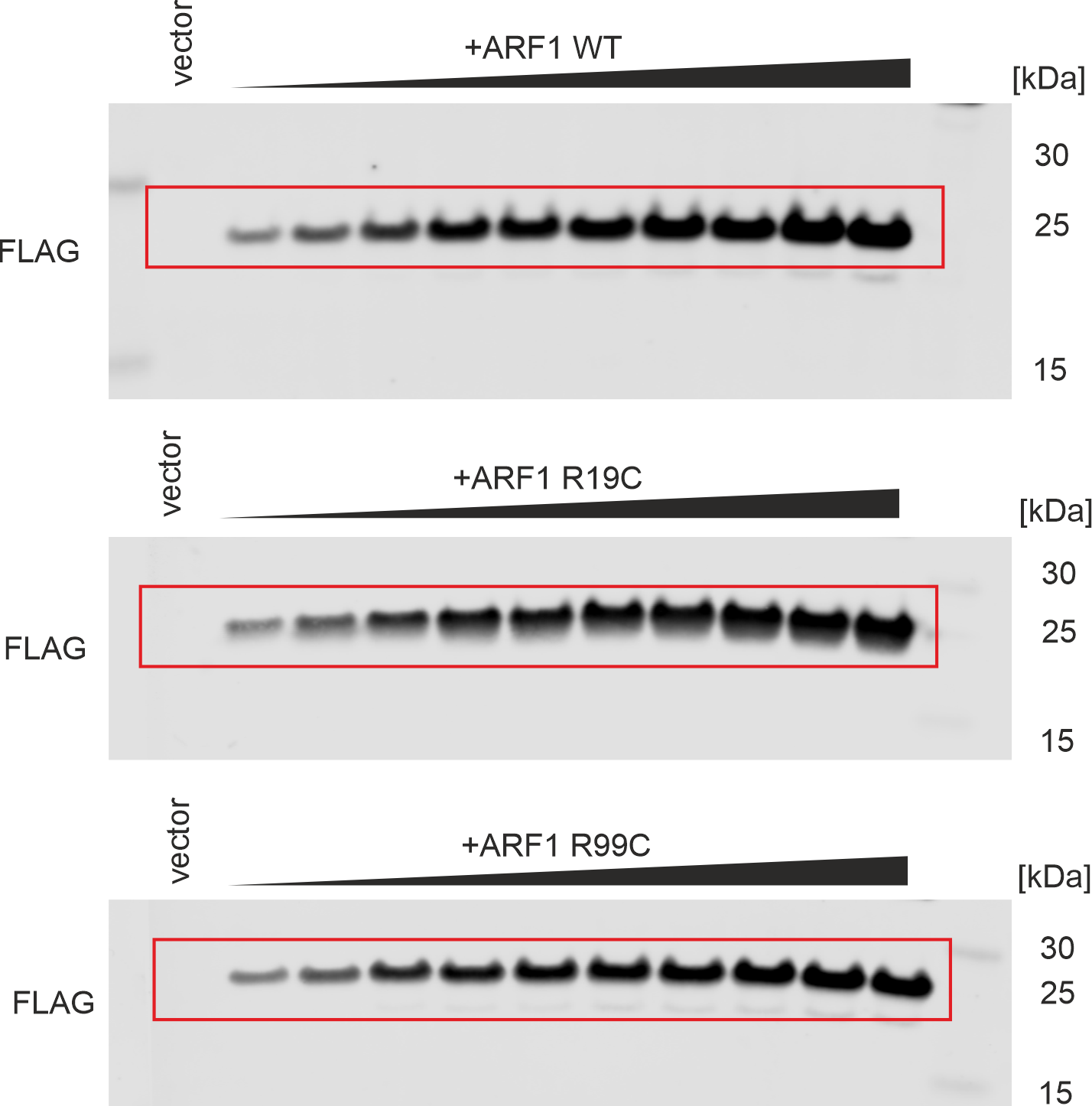

Supplement: Supplementary file 3 — Source data Fig. 1 [file 44319_2025_423_MOESM3_ESM.zip › Figure 1/1D/Western Blot FLAG WT, R19C, R99C.png]

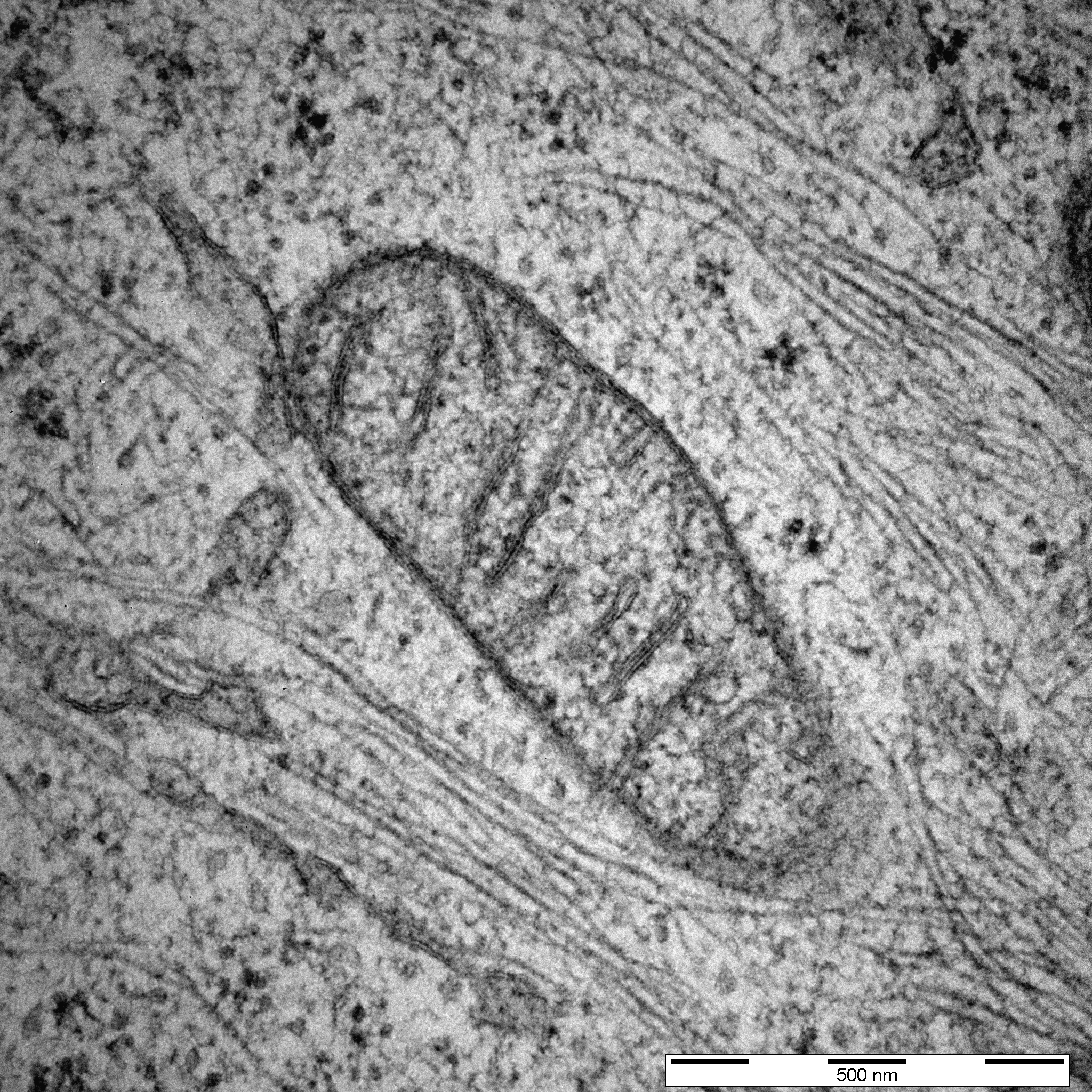

Supplement: Supplementary file 4 — Source data Fig. 2 [file 44319_2025_423_MOESM4_ESM.zip › Figure 2/2A/R19C inset.tif]

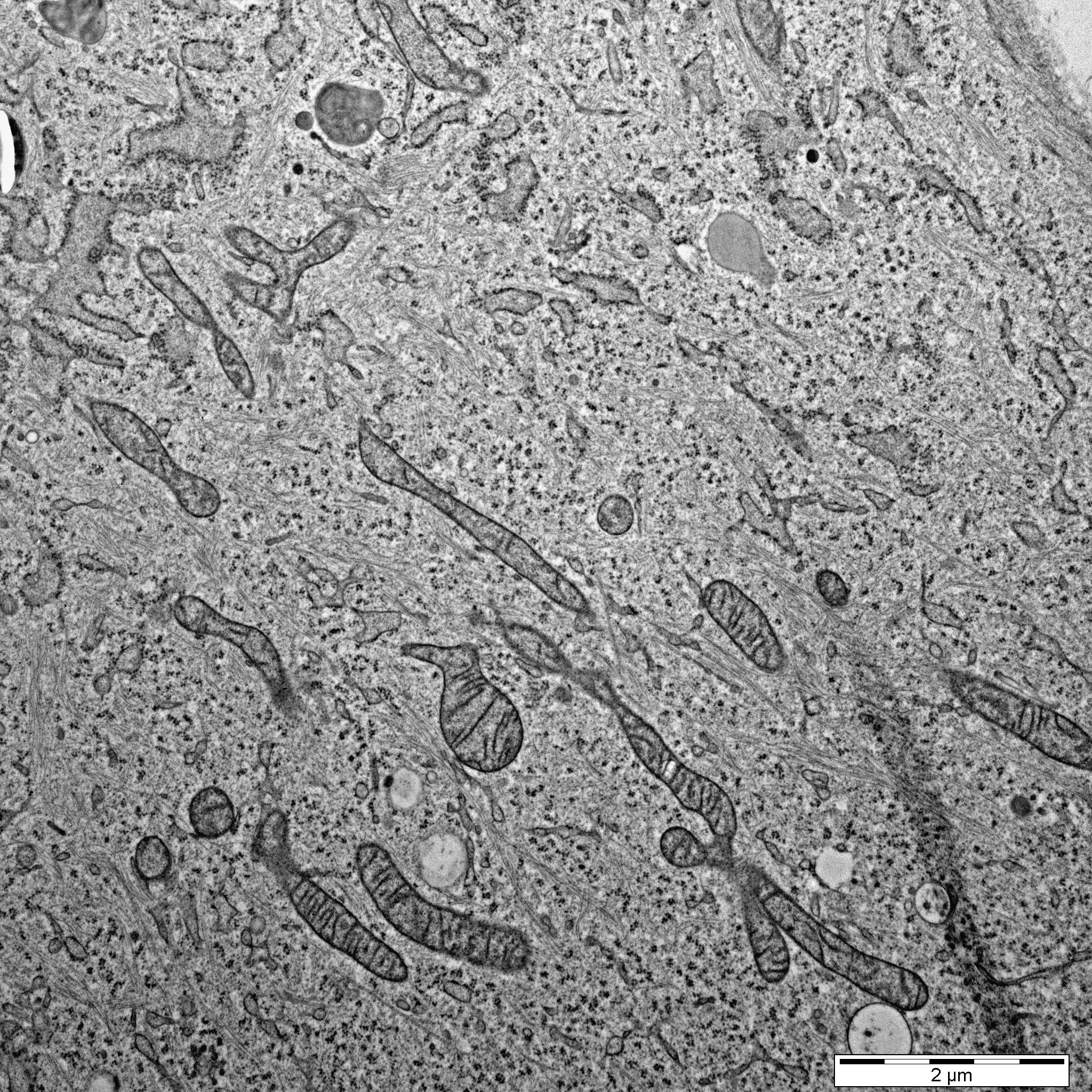

Supplement: Supplementary file 4 — Source data Fig. 2 [file 44319_2025_423_MOESM4_ESM.zip › Figure 2/2A/R19C overview.tif]

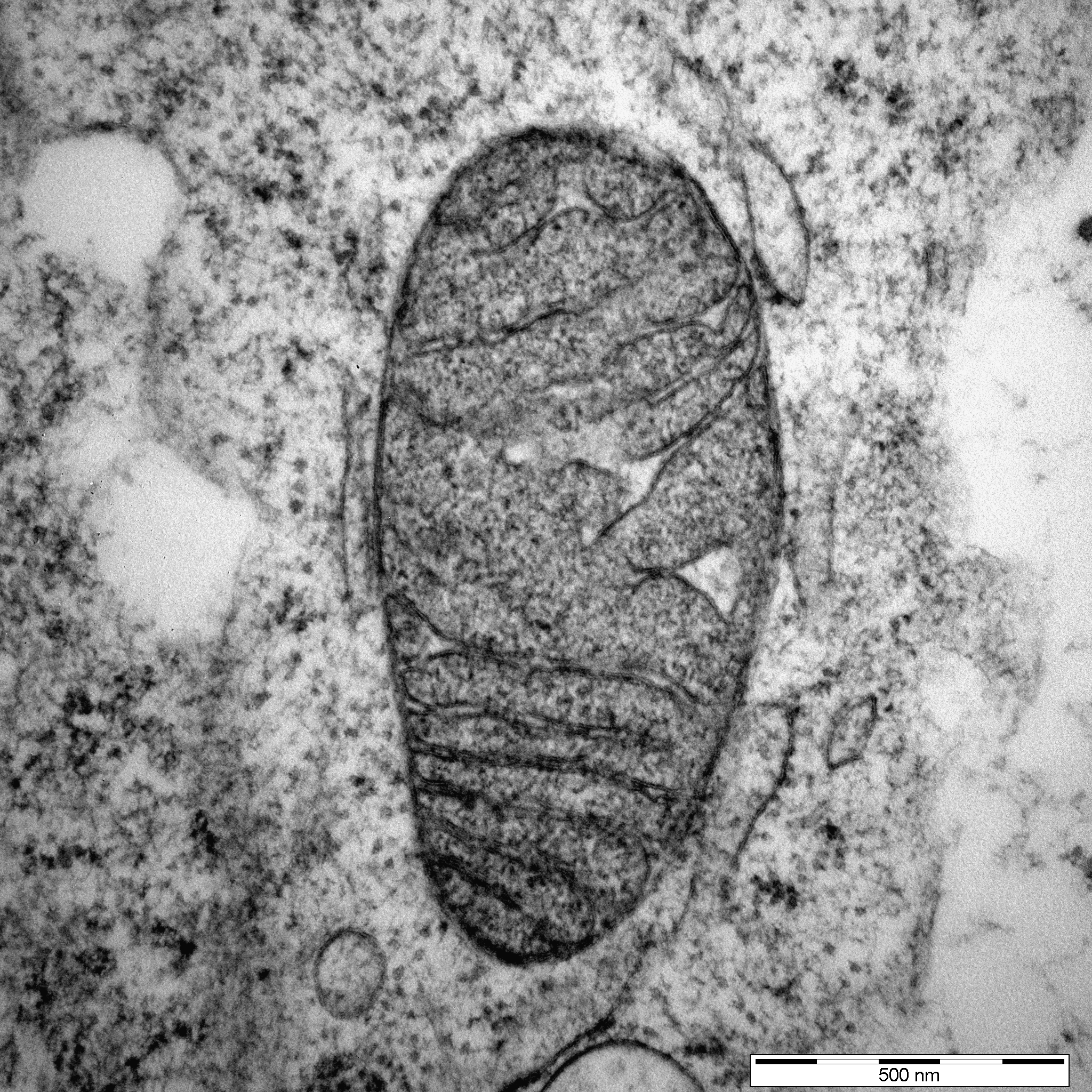

Supplement: Supplementary file 4 — Source data Fig. 2 [file 44319_2025_423_MOESM4_ESM.zip › Figure 2/2A/R99C inset.tif]

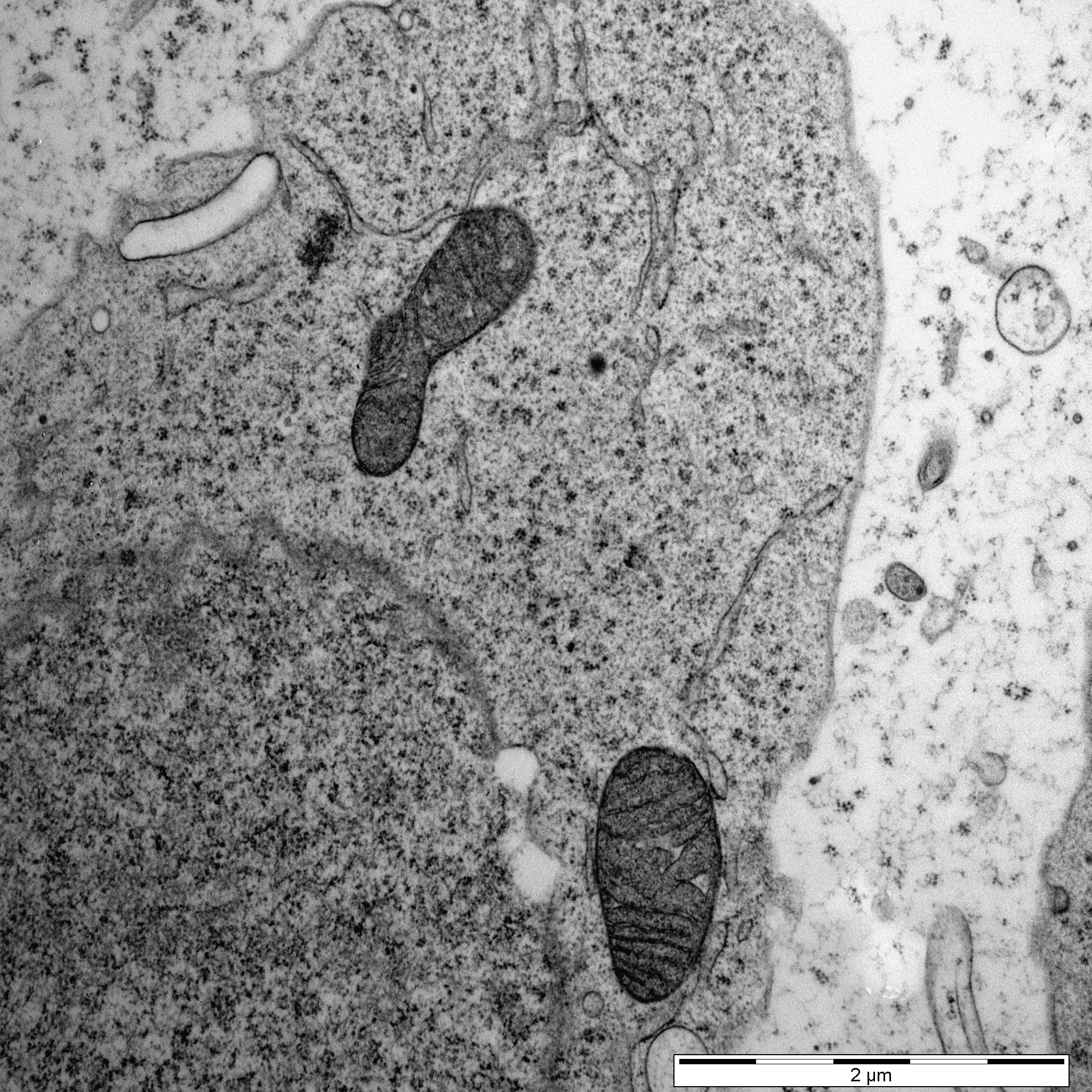

Supplement: Supplementary file 4 — Source data Fig. 2 [file 44319_2025_423_MOESM4_ESM.zip › Figure 2/2A/R99C overview.tif]

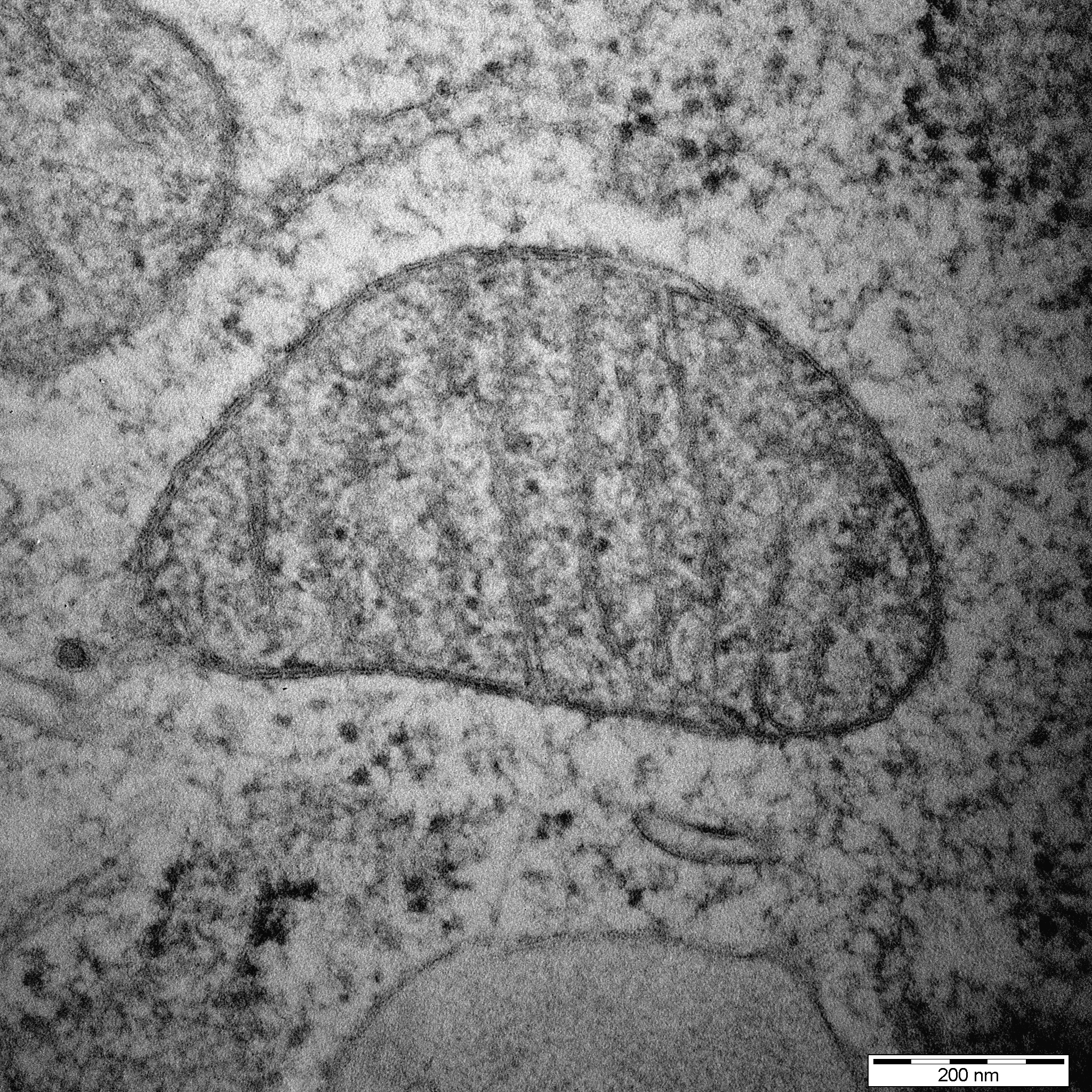

Supplement: Supplementary file 4 — Source data Fig. 2 [file 44319_2025_423_MOESM4_ESM.zip › Figure 2/2A/WT inset.tif]

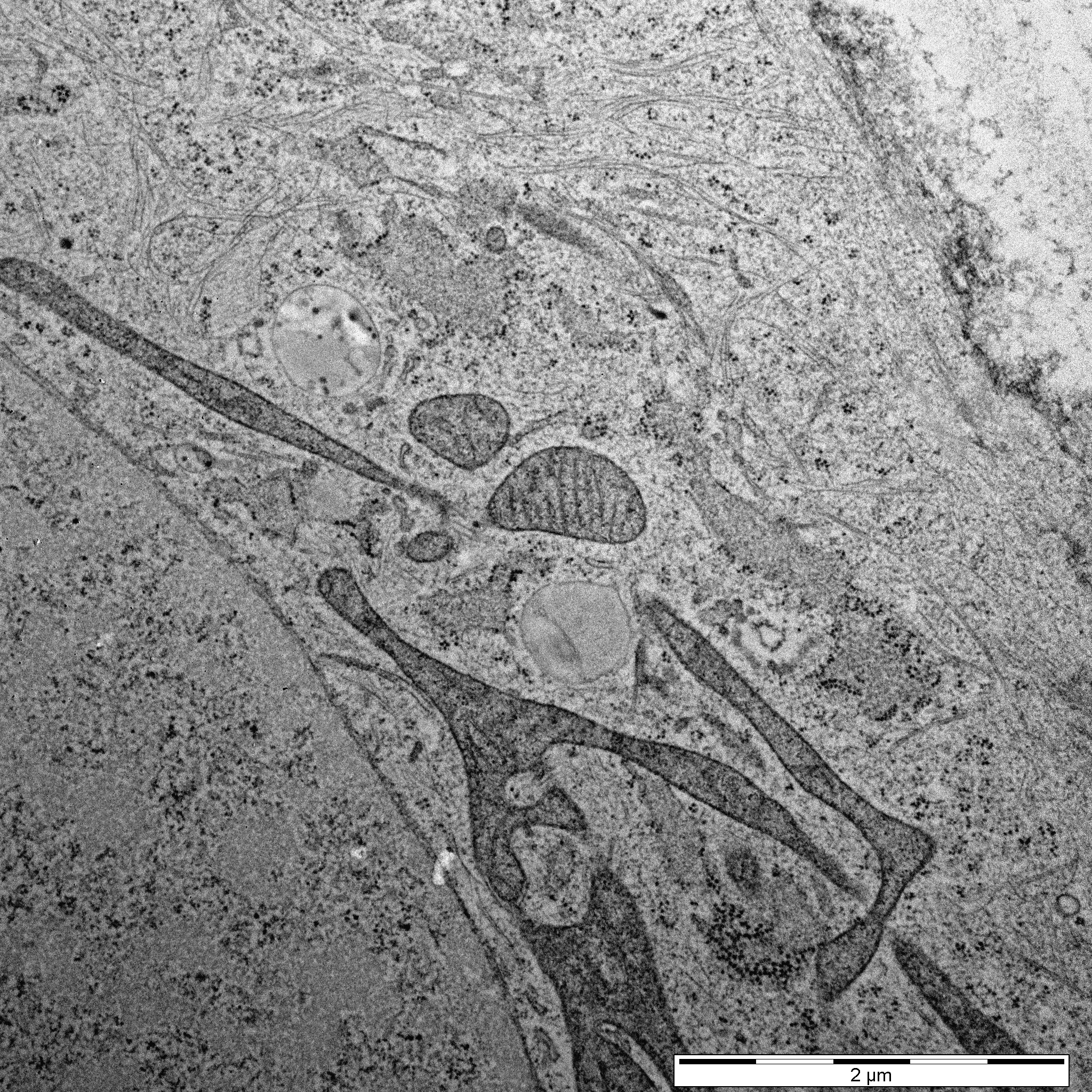

Supplement: Supplementary file 4 — Source data Fig. 2 [file 44319_2025_423_MOESM4_ESM.zip › Figure 2/2A/WT overview.tif]

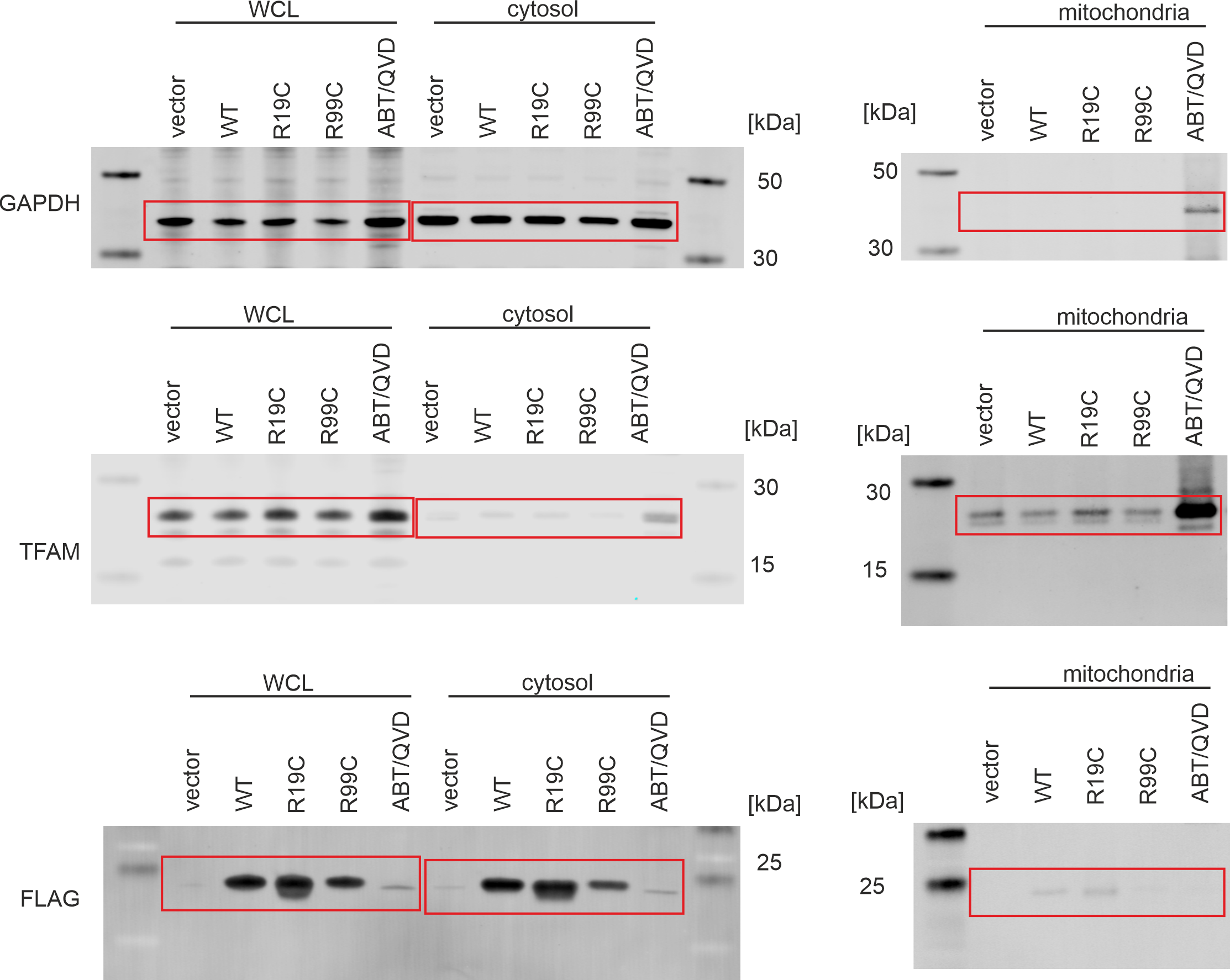

Supplement: Supplementary file 4 — Source data Fig. 2 [file 44319_2025_423_MOESM4_ESM.zip › Figure 2/2B/Western Blot FLAG, TFAM, GAPDH - vector, WT, R19C, R99C, ABT-QVD.png]

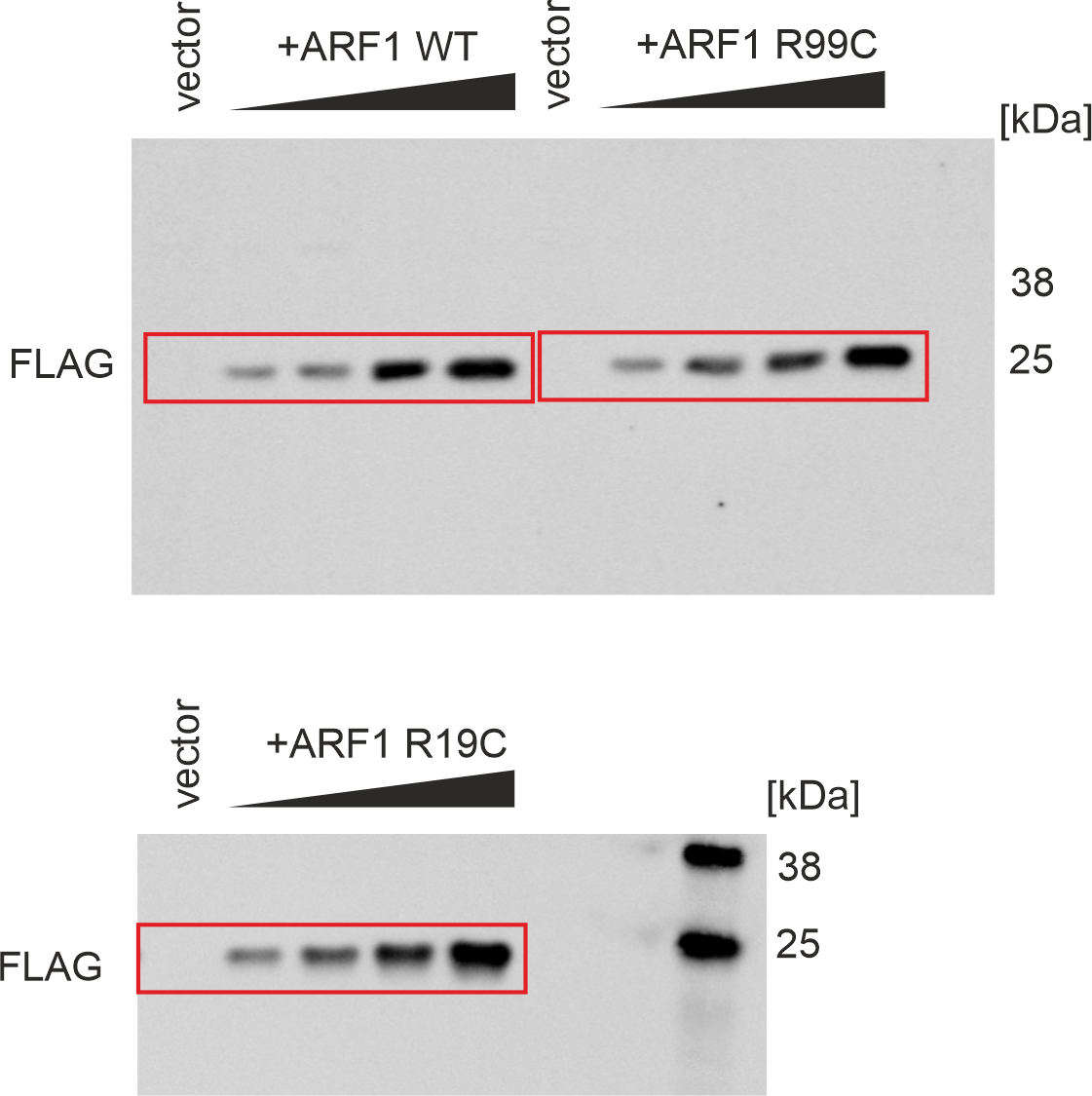

Supplement: Supplementary file 4 — Source data Fig. 2 [file 44319_2025_423_MOESM4_ESM.zip › Figure 2/2D/Western Blot FLAG WT, R19C, R99C.png]

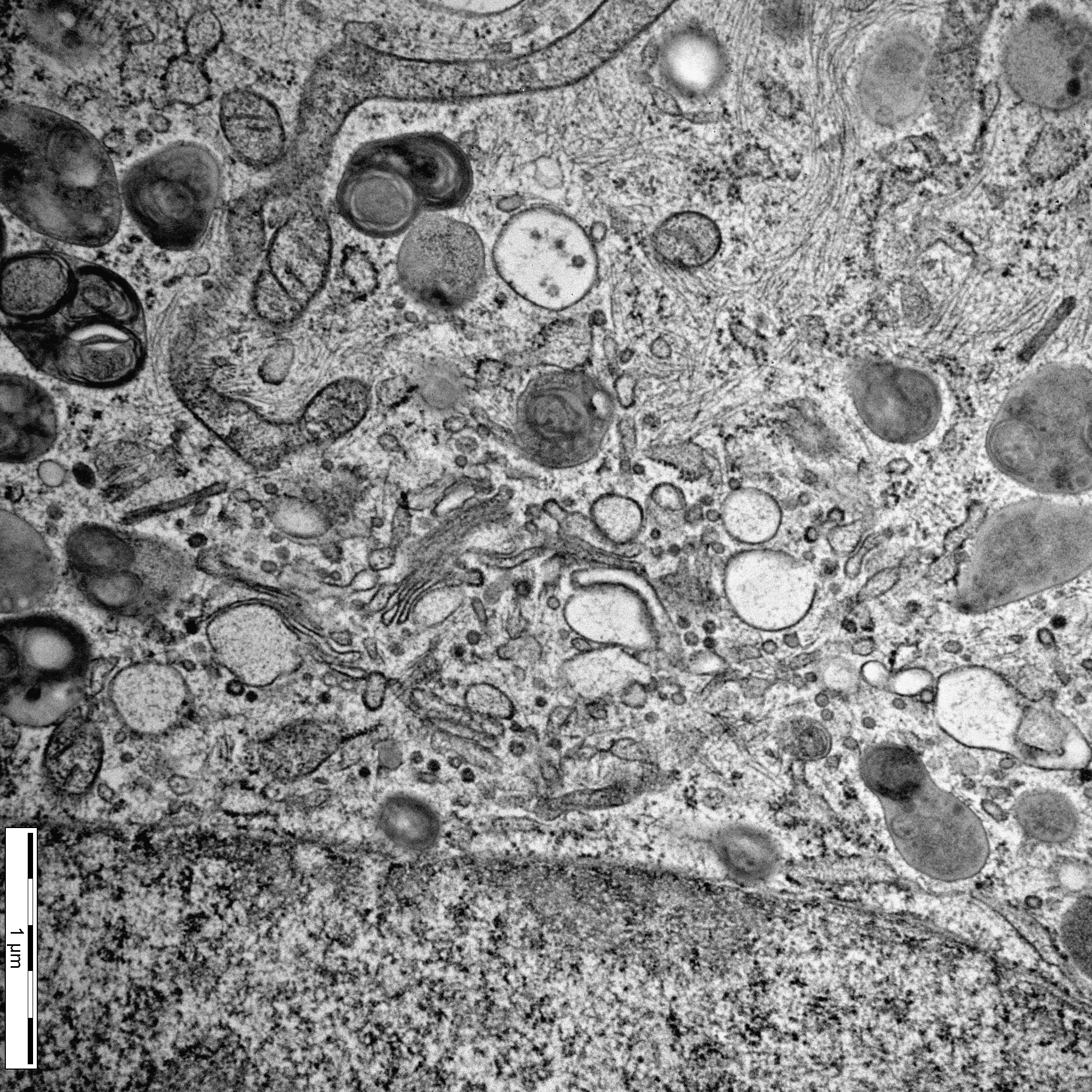

Supplement: Supplementary file 5 — Source data Fig. 3 [file 44319_2025_423_MOESM5_ESM.zip › Figure 3/3A/R19C_overview.tif]

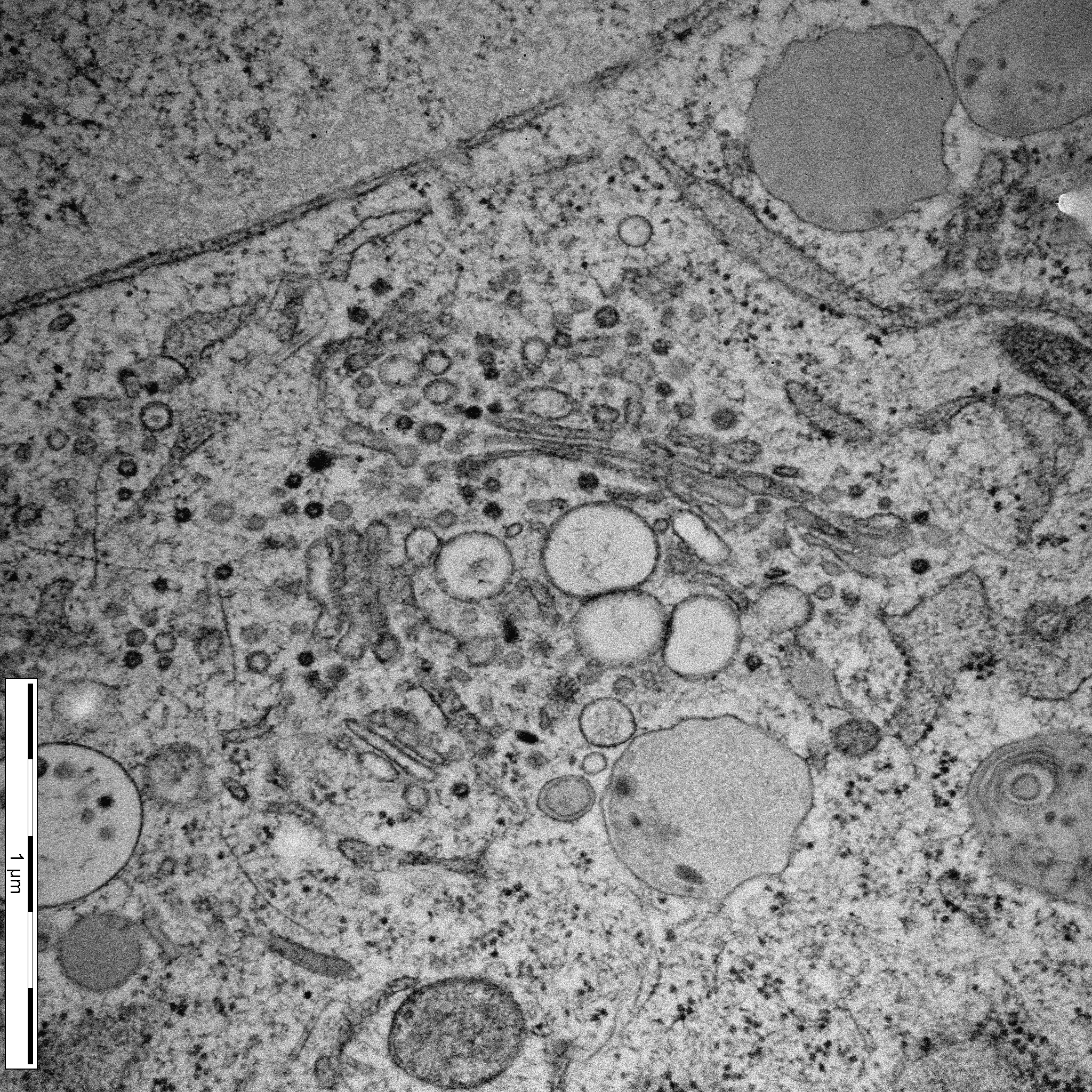

Supplement: Supplementary file 5 — Source data Fig. 3 [file 44319_2025_423_MOESM5_ESM.zip › Figure 3/3A/WT_overview.tif]

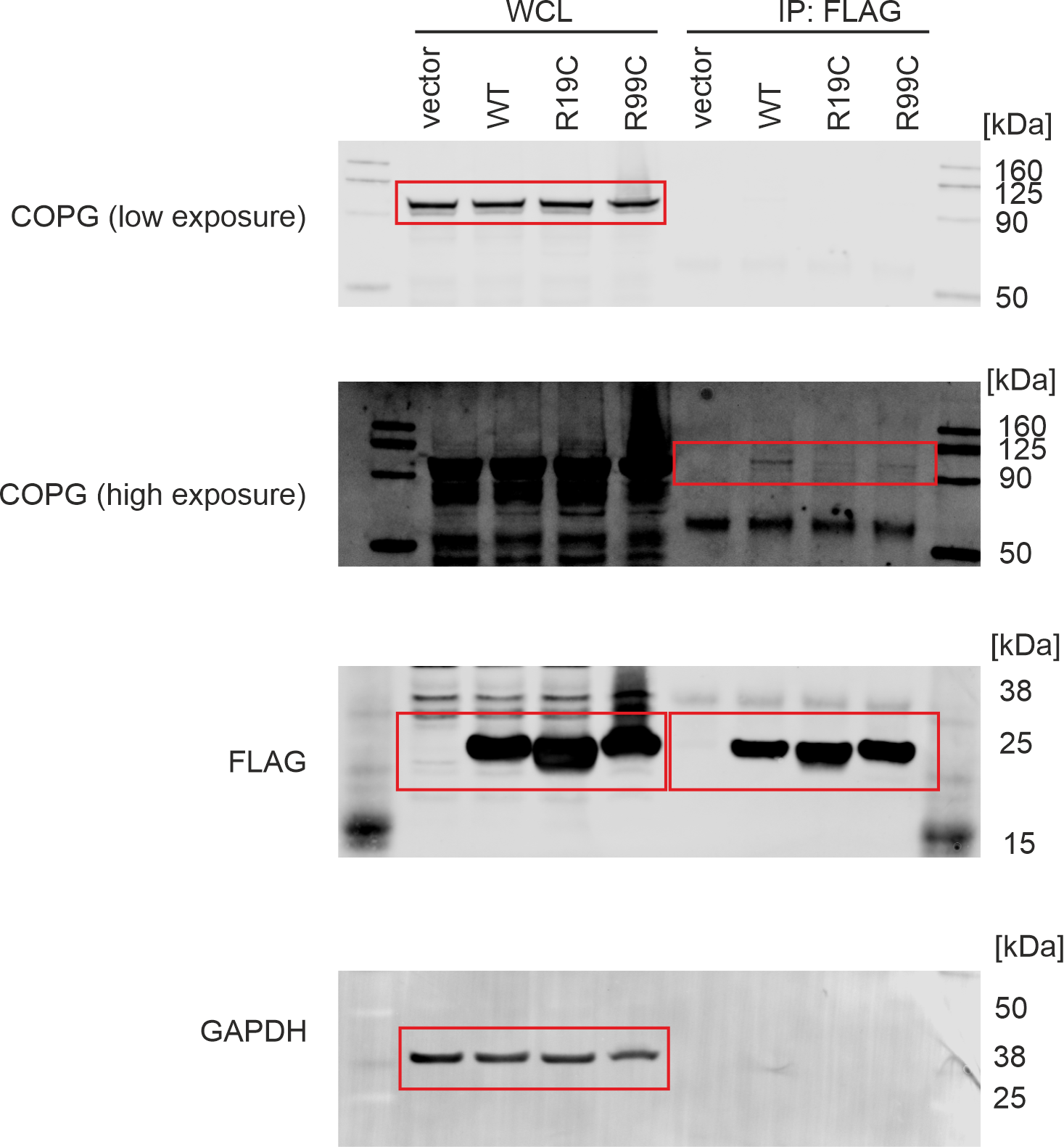

Supplement: Supplementary file 5 — Source data Fig. 3 [file 44319_2025_423_MOESM5_ESM.zip › Figure 3/3C/Western Blot COPG, FLAG, GAPDH - vector, WT, R19C, R99C.png]

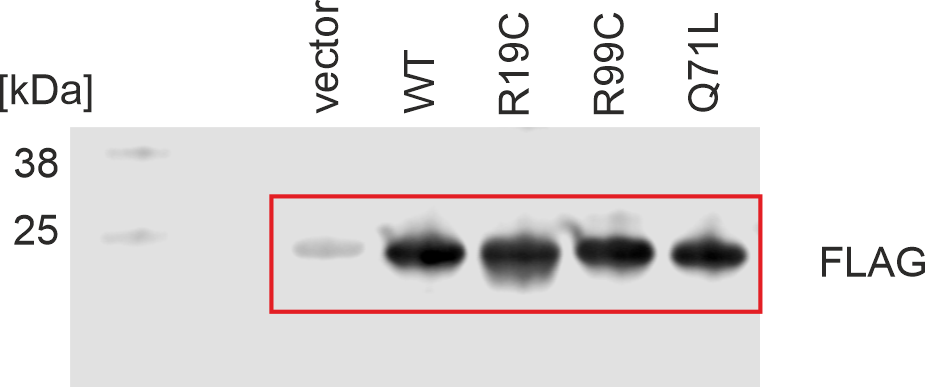

Supplement: Supplementary file 5 — Source data Fig. 3 [file 44319_2025_423_MOESM5_ESM.zip › Figure 3/3D/Western Blot FLAG - vector, WT, R19C, R99C, Q71L.png]

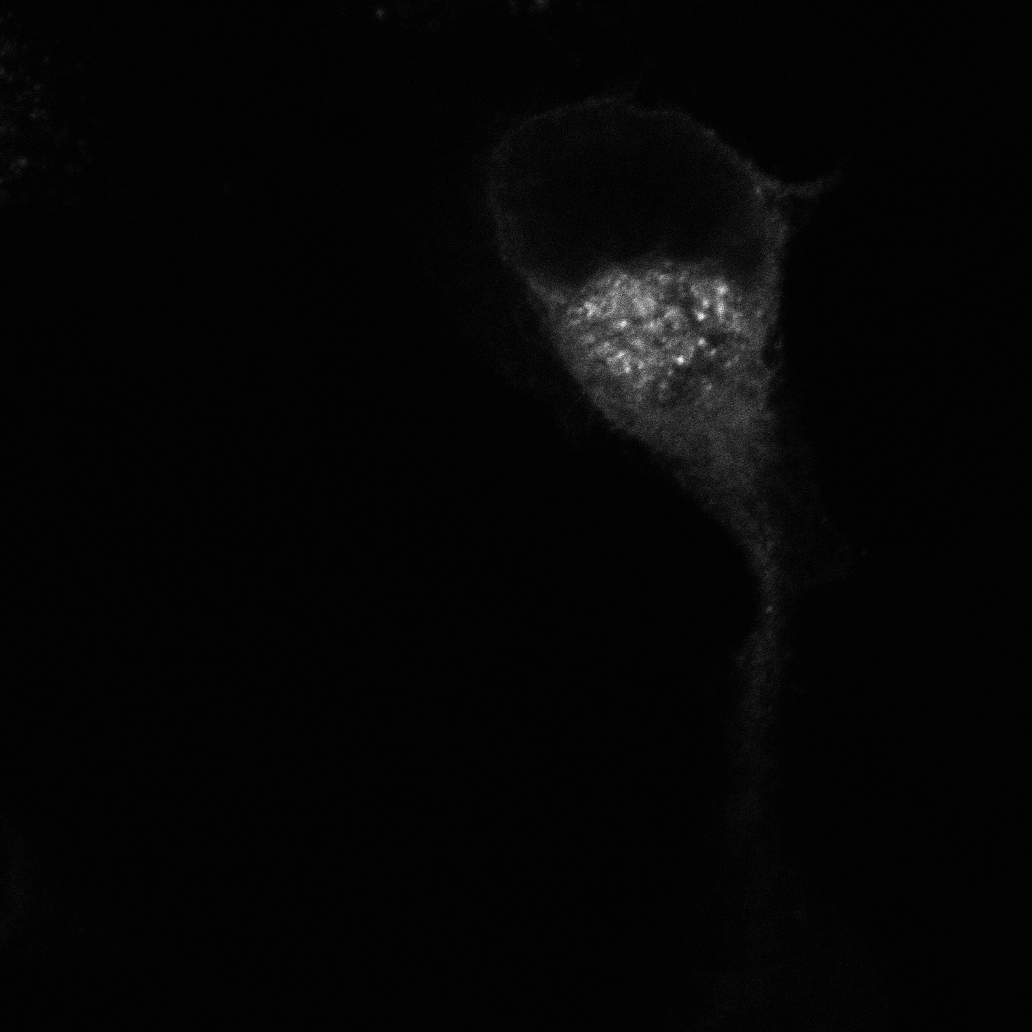

Supplement: Supplementary file 6 — Source data Fig. 4 [file 44319_2025_423_MOESM6_ESM.zip › Figure 4/4A/R19C.tif]

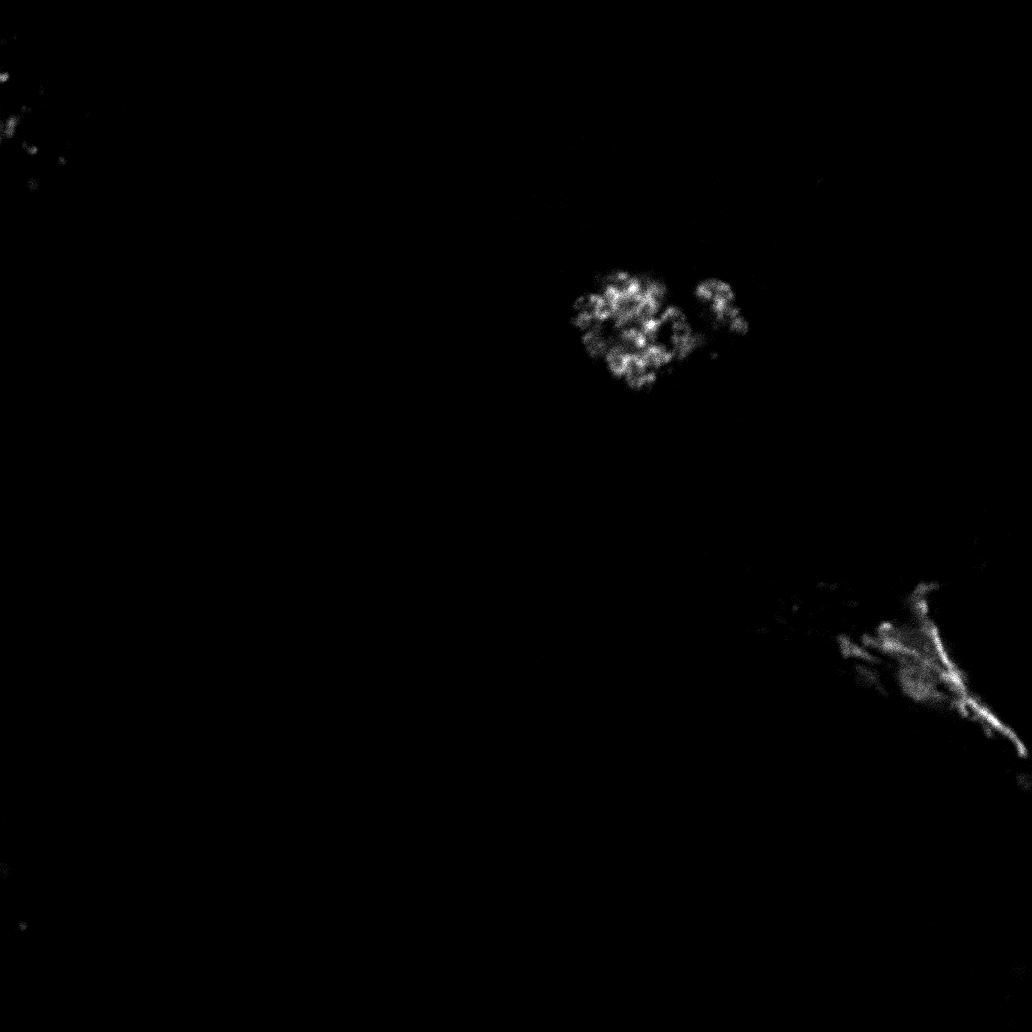

Supplement: Supplementary file 6 — Source data Fig. 4 [file 44319_2025_423_MOESM6_ESM.zip › Figure 4/4A/R19C_GM130.tif]

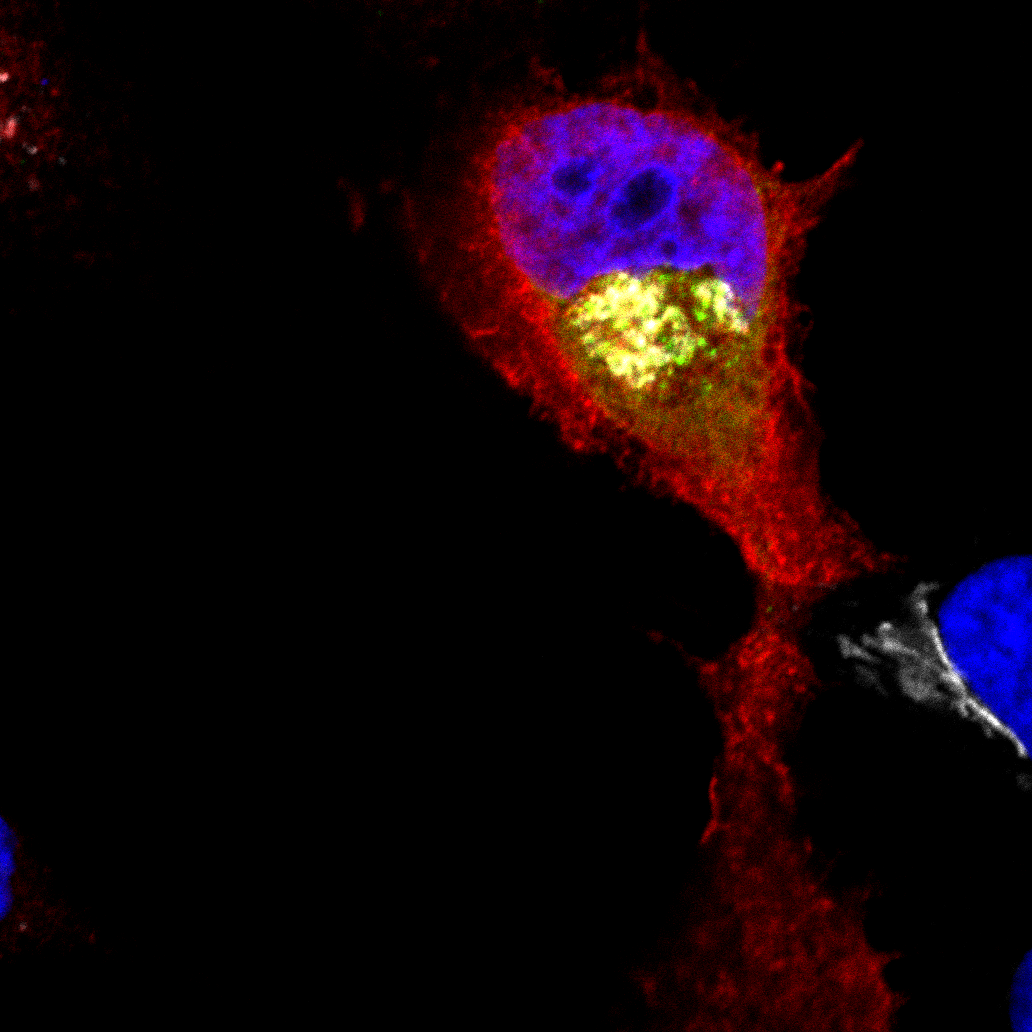

Supplement: Supplementary file 6 — Source data Fig. 4 [file 44319_2025_423_MOESM6_ESM.zip › Figure 4/4A/R19C_merge.tif]

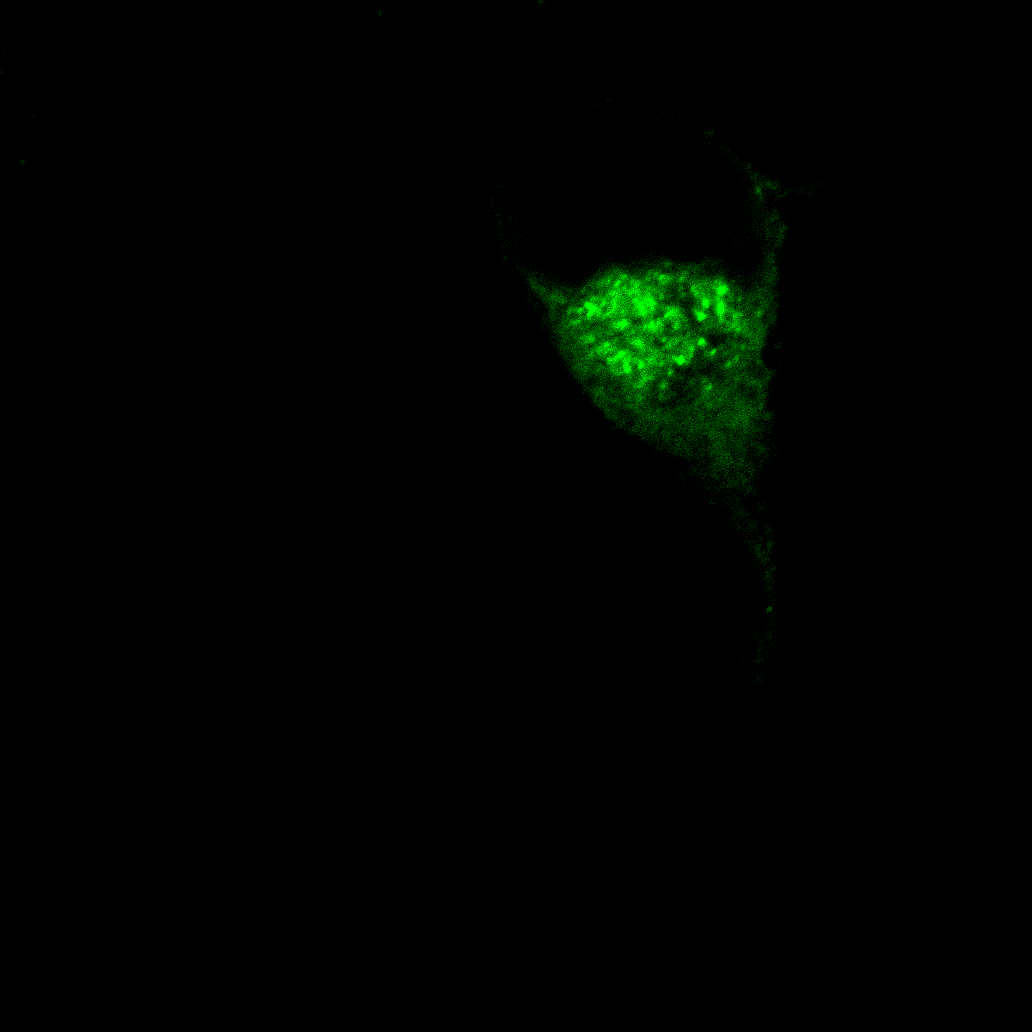

Supplement: Supplementary file 6 — Source data Fig. 4 [file 44319_2025_423_MOESM6_ESM.zip › Figure 4/4A/R19C_STING.tif]

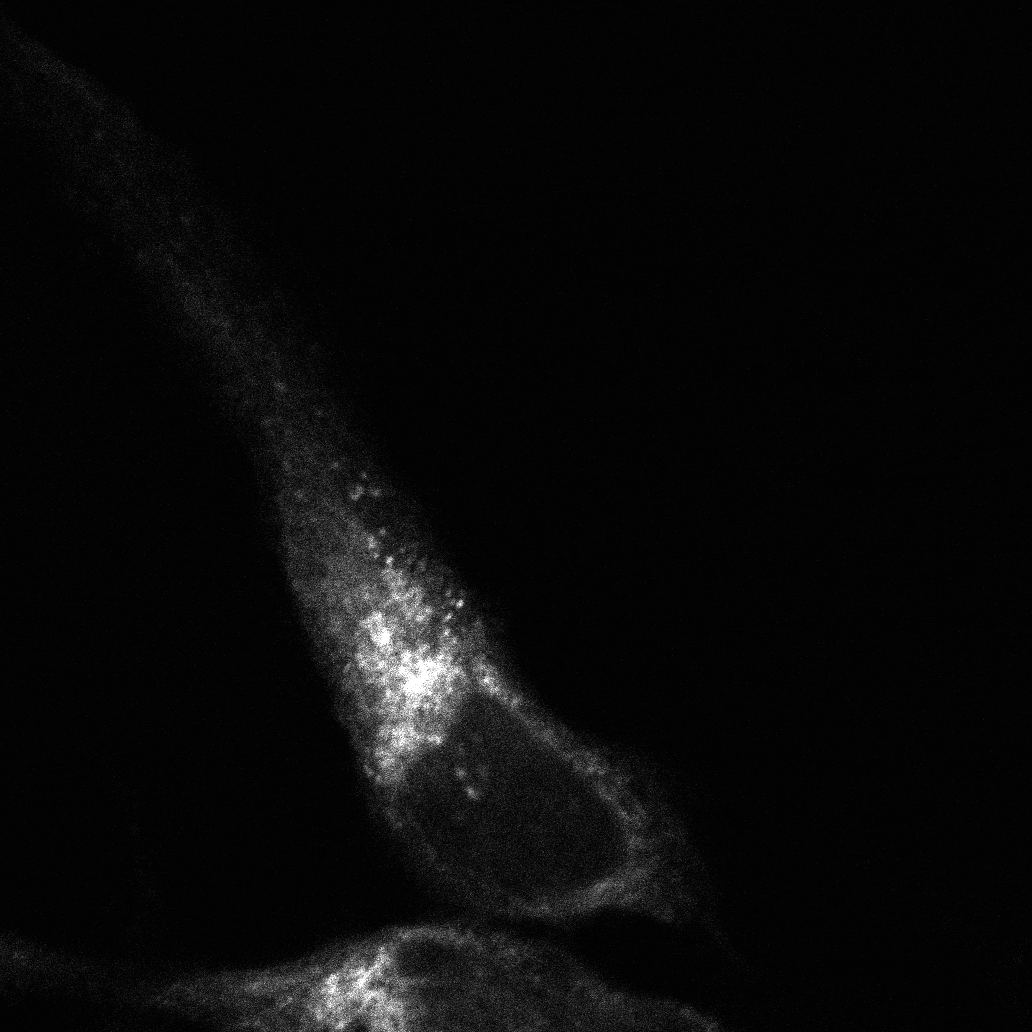

Supplement: Supplementary file 6 — Source data Fig. 4 [file 44319_2025_423_MOESM6_ESM.zip › Figure 4/4A/R99C.tif]

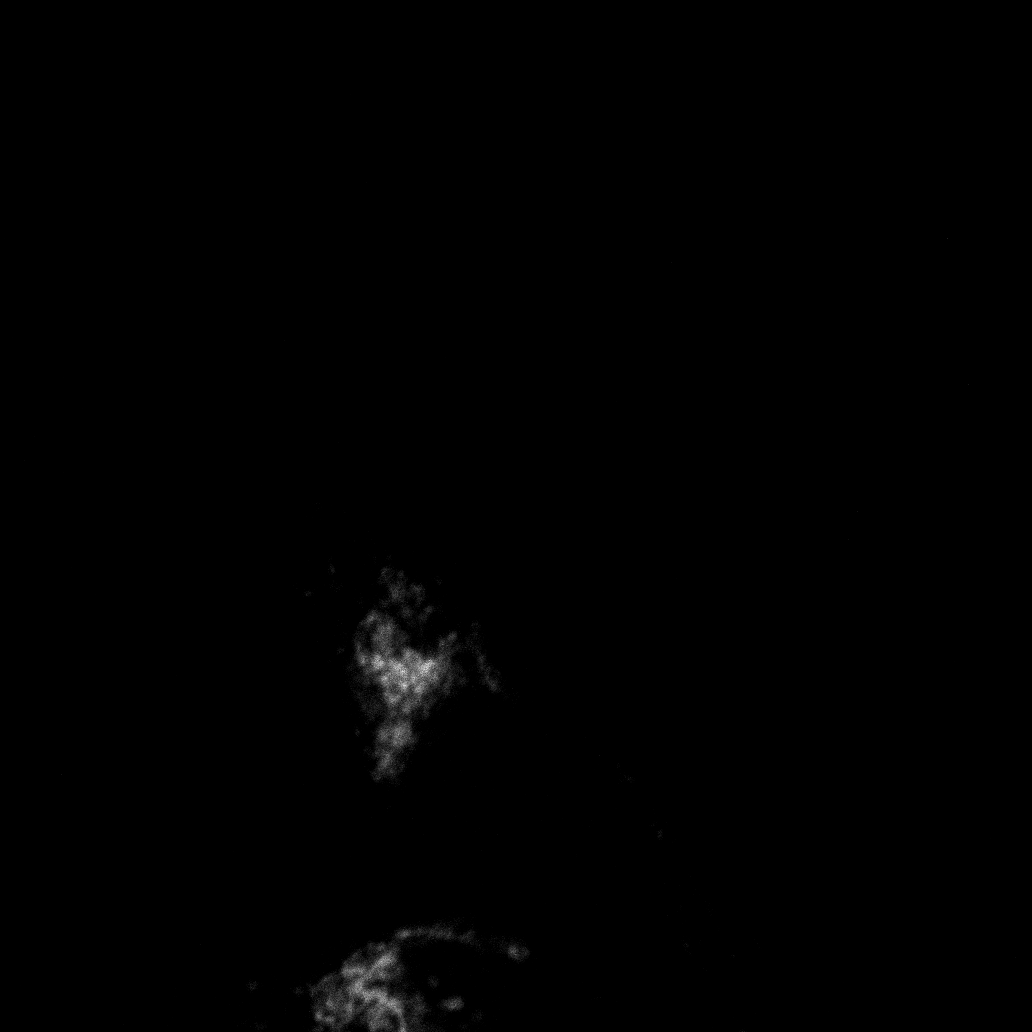

Supplement: Supplementary file 6 — Source data Fig. 4 [file 44319_2025_423_MOESM6_ESM.zip › Figure 4/4A/R99C_GM130.tif]

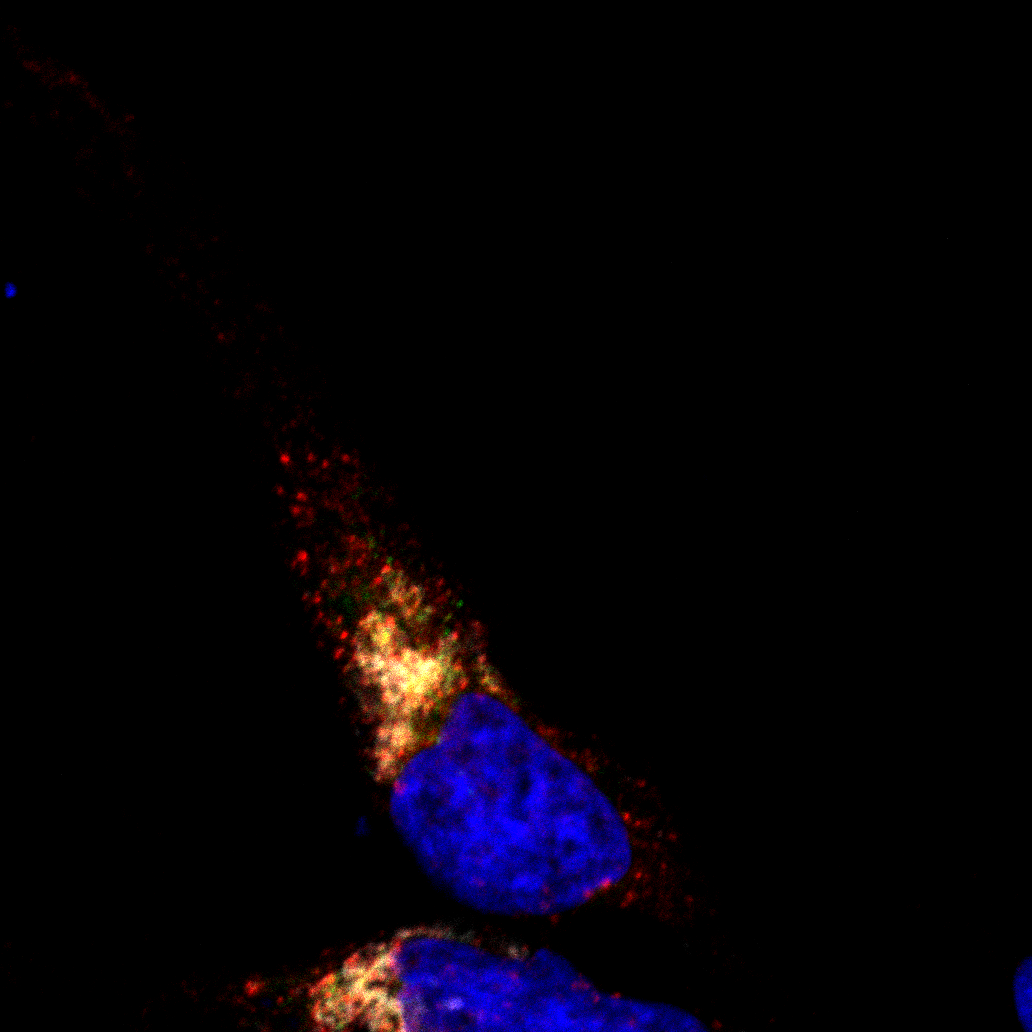

Supplement: Supplementary file 6 — Source data Fig. 4 [file 44319_2025_423_MOESM6_ESM.zip › Figure 4/4A/R99C_merge.tif]

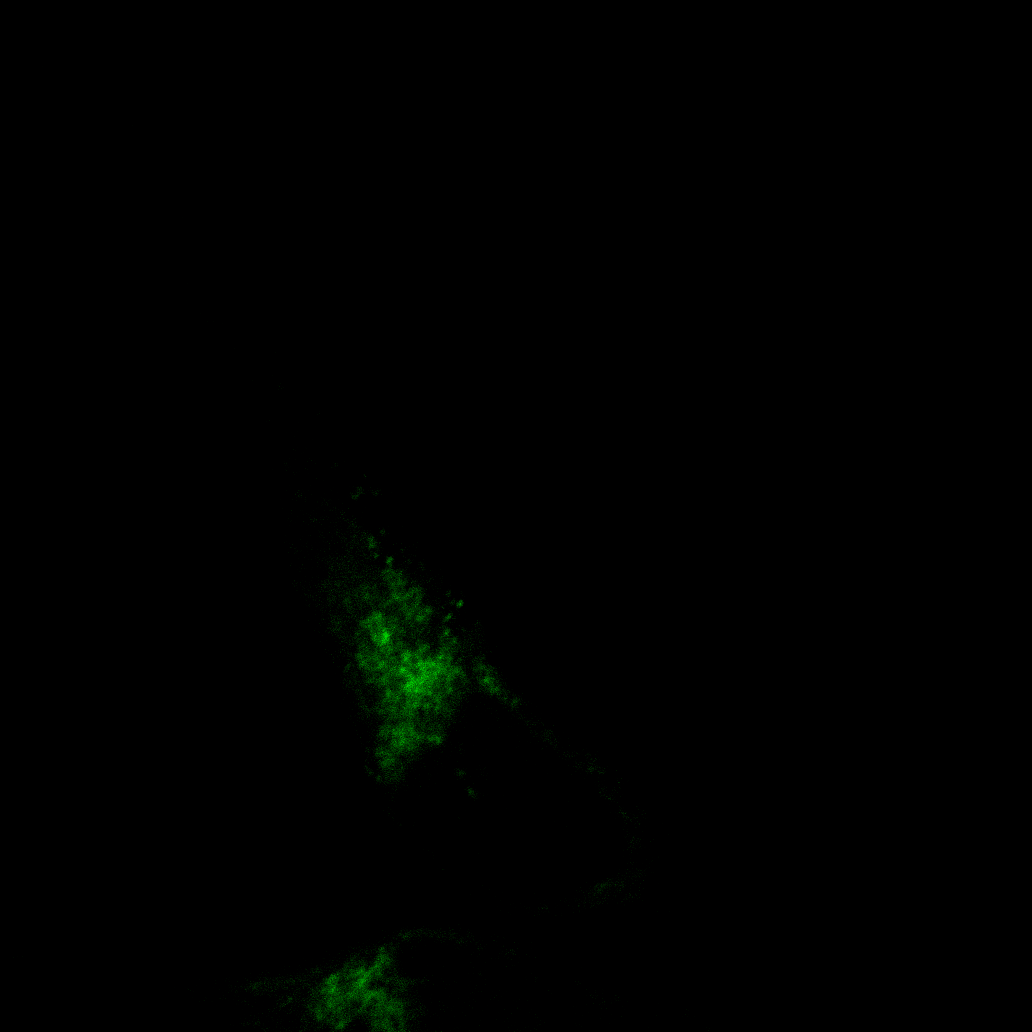

Supplement: Supplementary file 6 — Source data Fig. 4 [file 44319_2025_423_MOESM6_ESM.zip › Figure 4/4A/R99C_STING.tif]

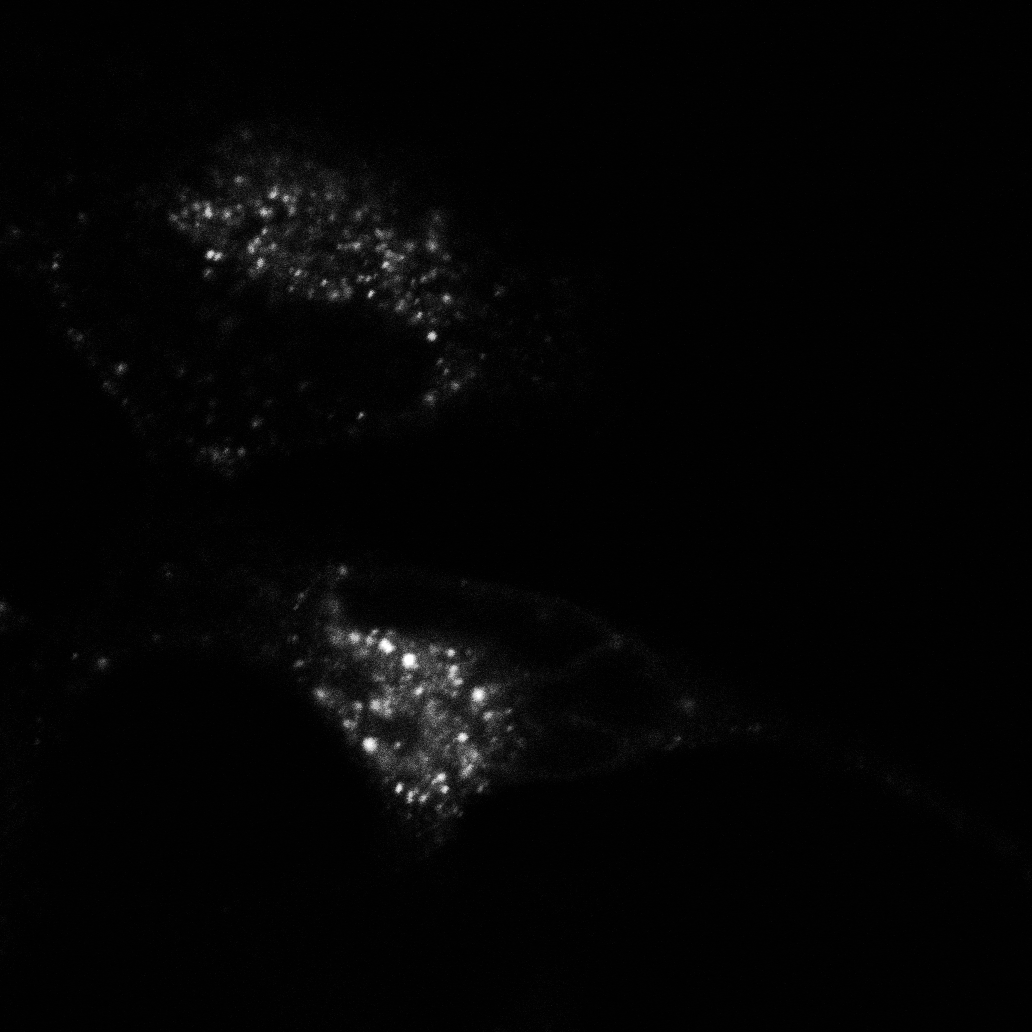

Supplement: Supplementary file 6 — Source data Fig. 4 [file 44319_2025_423_MOESM6_ESM.zip › Figure 4/4A/vector.tif]

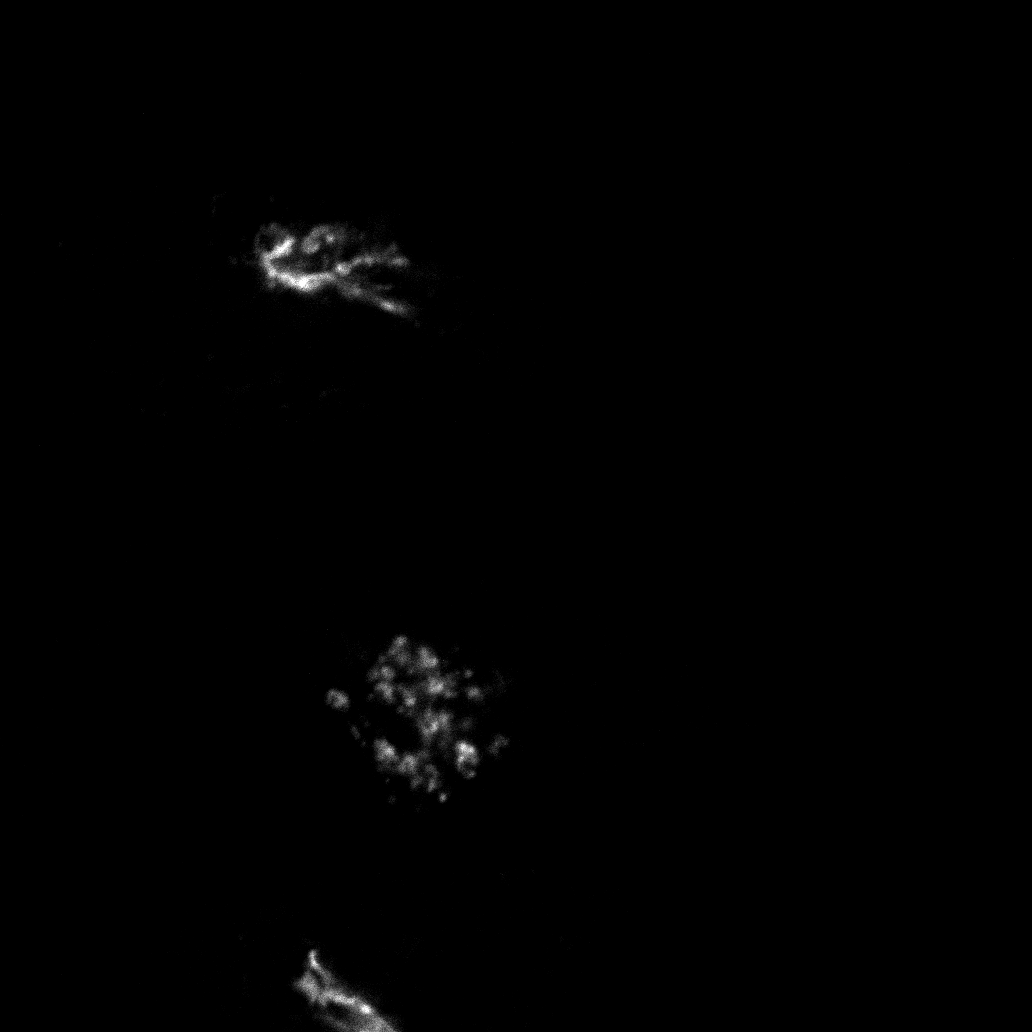

Supplement: Supplementary file 6 — Source data Fig. 4 [file 44319_2025_423_MOESM6_ESM.zip › Figure 4/4A/vector_GM130.tif]

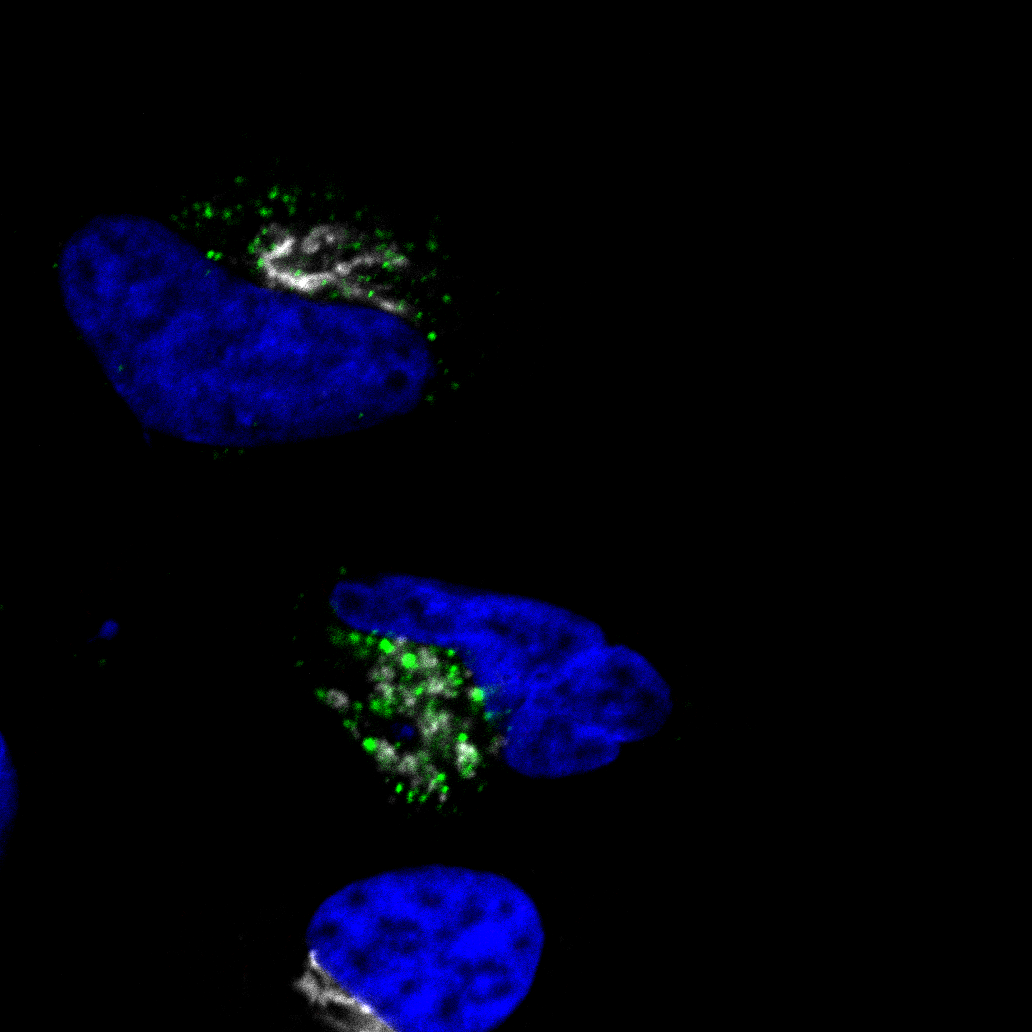

Supplement: Supplementary file 6 — Source data Fig. 4 [file 44319_2025_423_MOESM6_ESM.zip › Figure 4/4A/vector_merge.tif]

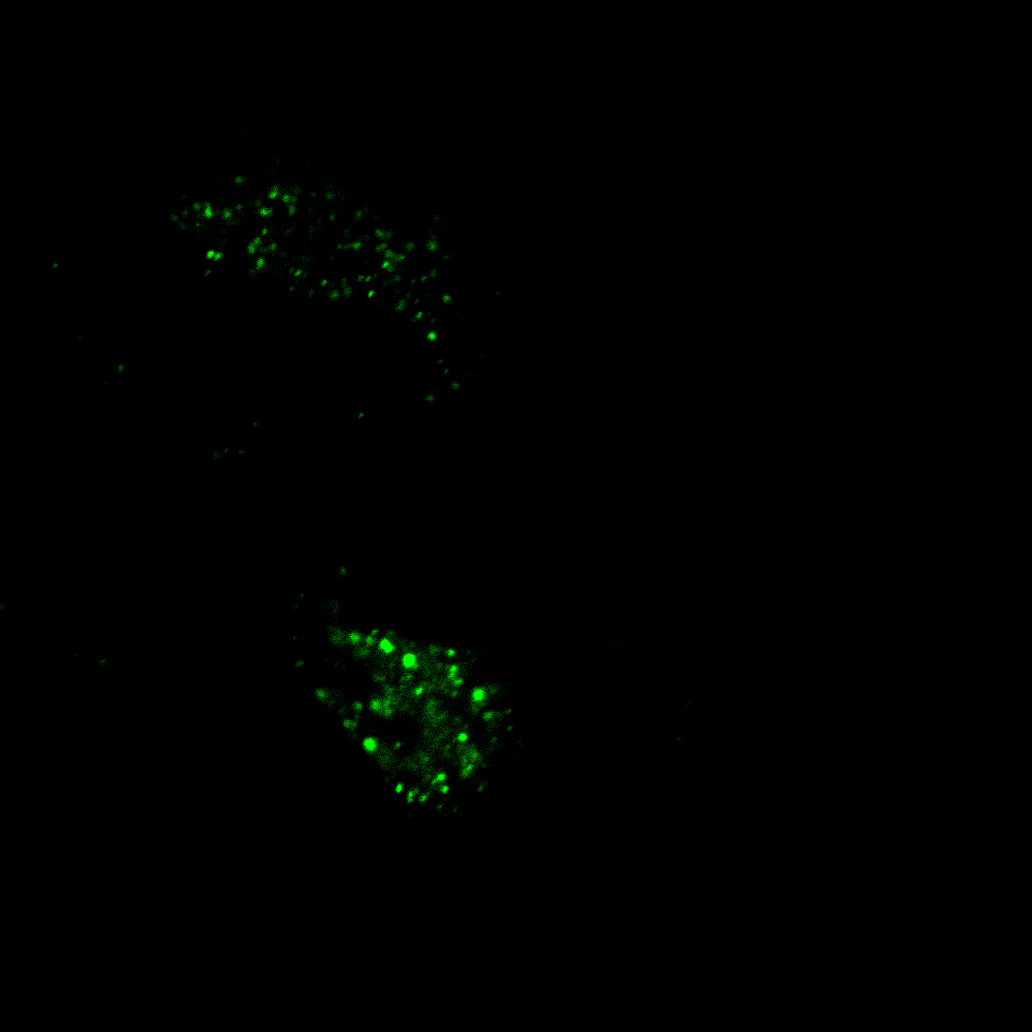

Supplement: Supplementary file 6 — Source data Fig. 4 [file 44319_2025_423_MOESM6_ESM.zip › Figure 4/4A/vector_STING.tif]

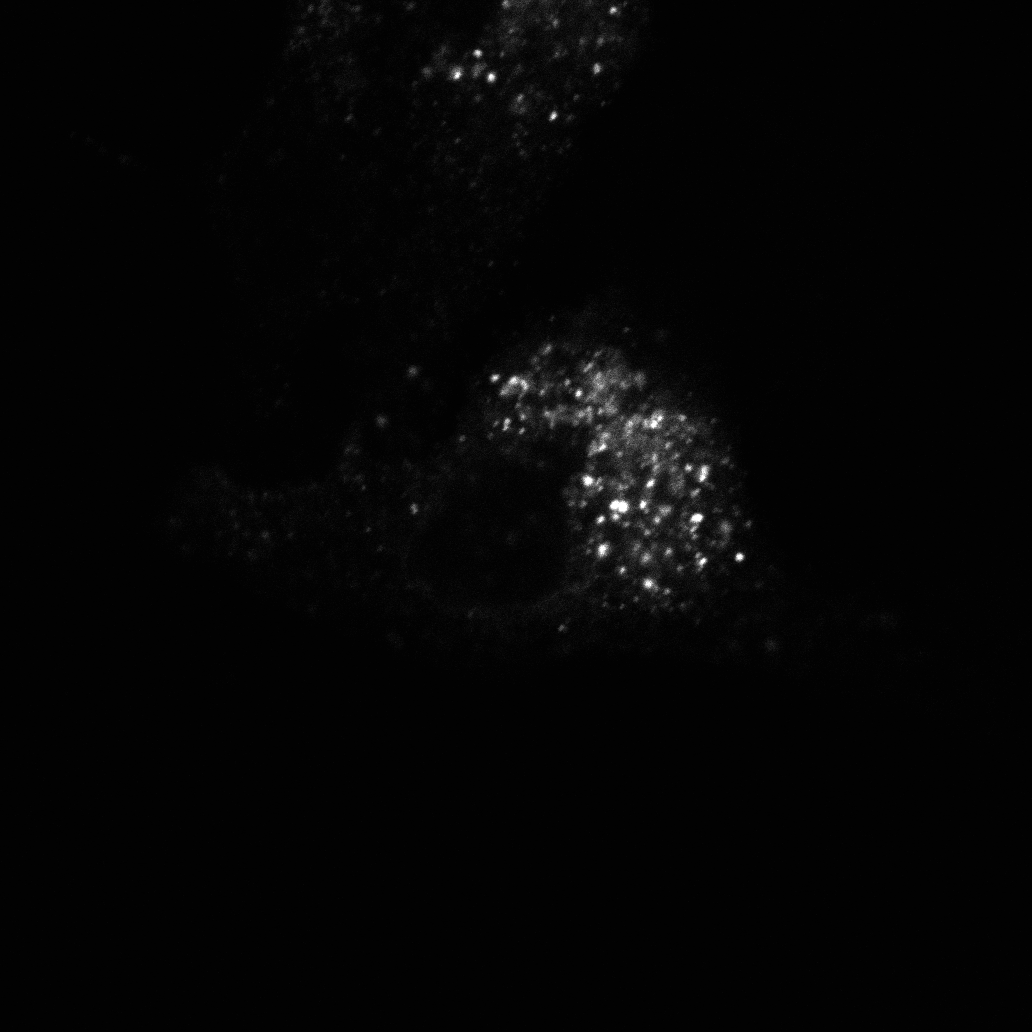

Supplement: Supplementary file 6 — Source data Fig. 4 [file 44319_2025_423_MOESM6_ESM.zip › Figure 4/4A/WT.tif]

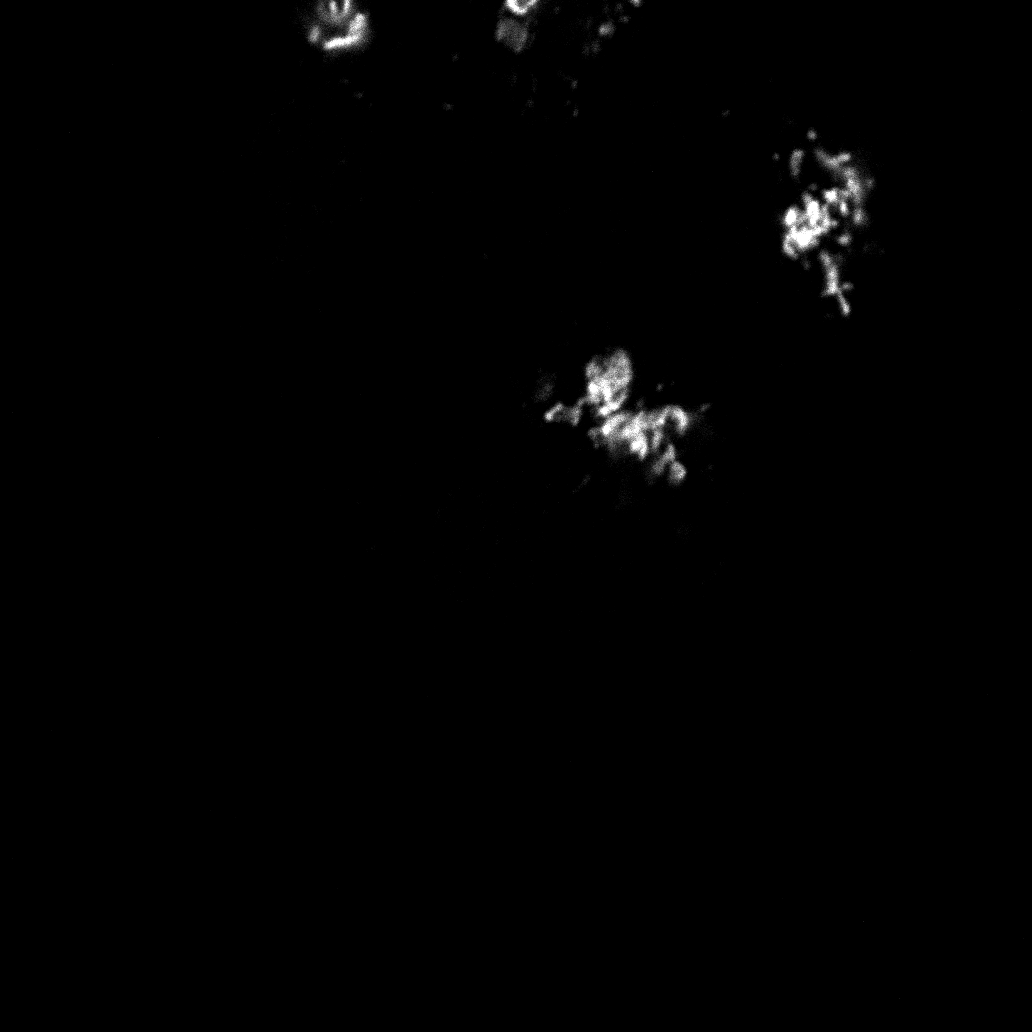

Supplement: Supplementary file 6 — Source data Fig. 4 [file 44319_2025_423_MOESM6_ESM.zip › Figure 4/4A/WT_GM130.tif]

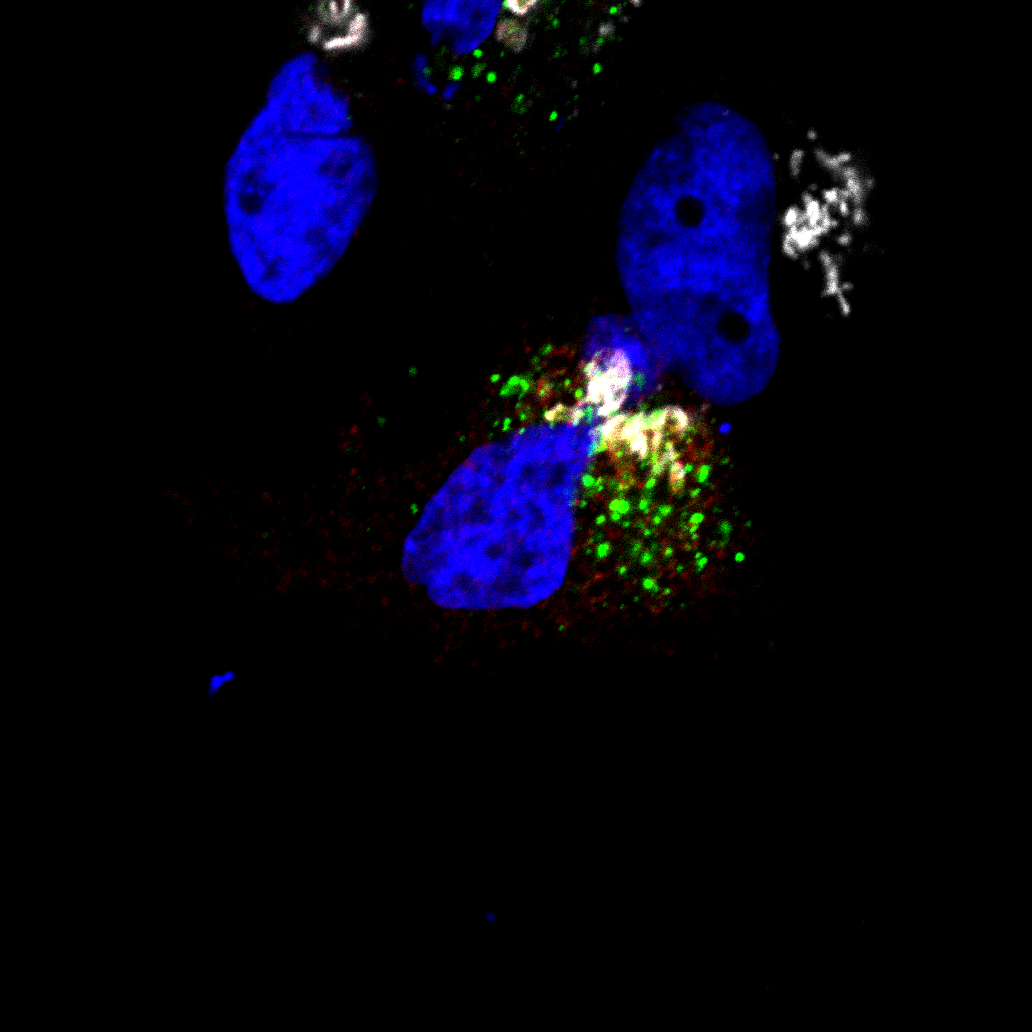

Supplement: Supplementary file 6 — Source data Fig. 4 [file 44319_2025_423_MOESM6_ESM.zip › Figure 4/4A/WT_merge.tif]

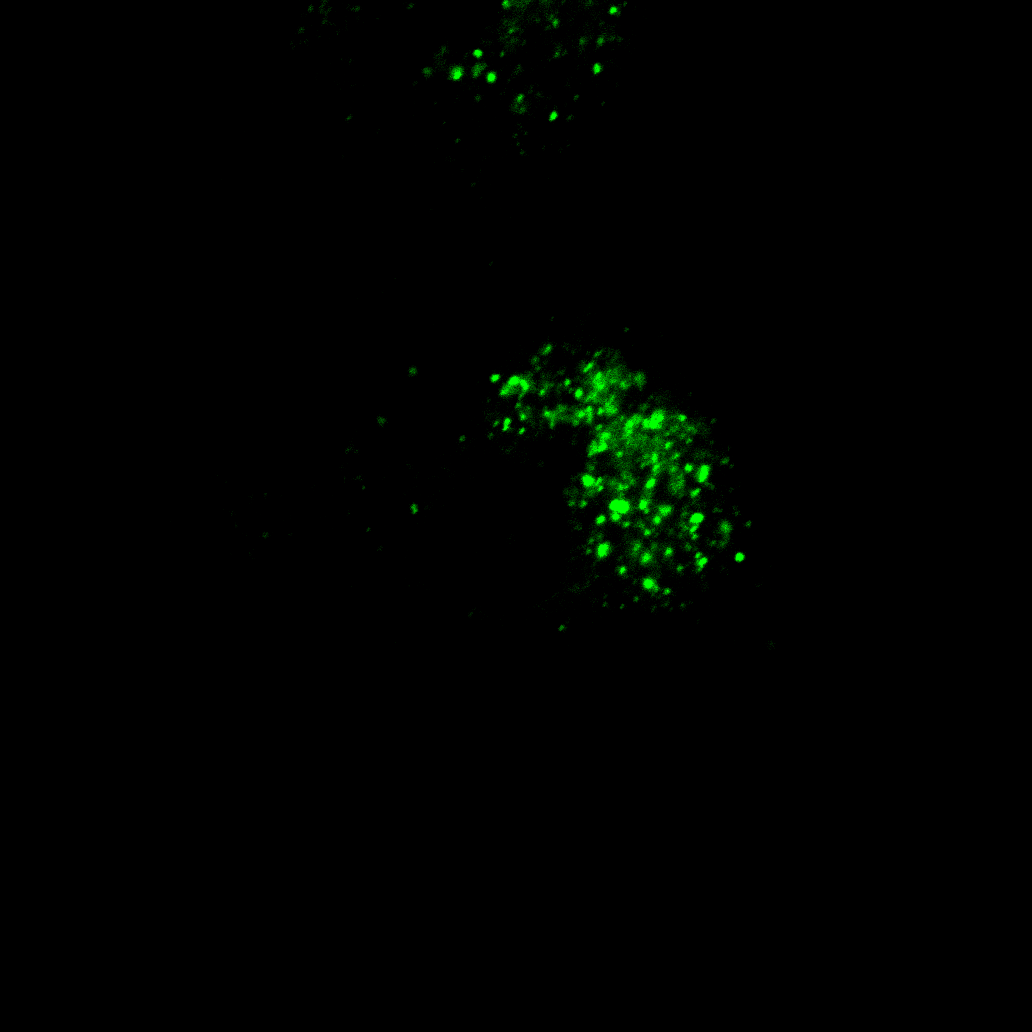

Supplement: Supplementary file 6 — Source data Fig. 4 [file 44319_2025_423_MOESM6_ESM.zip › Figure 4/4A/WT_STING.tif]

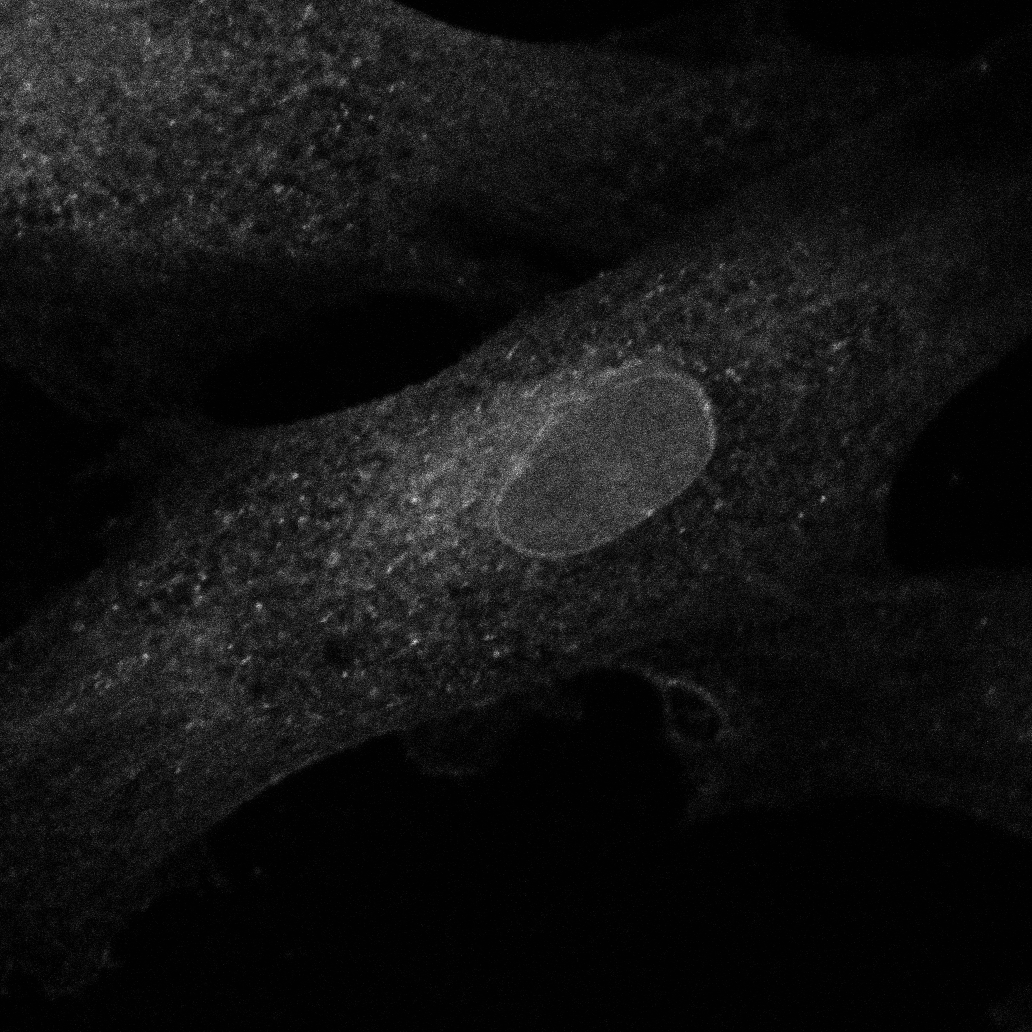

Supplement: Supplementary file 6 — Source data Fig. 4 [file 44319_2025_423_MOESM6_ESM.zip › Figure 4/4C/R19C.tif]

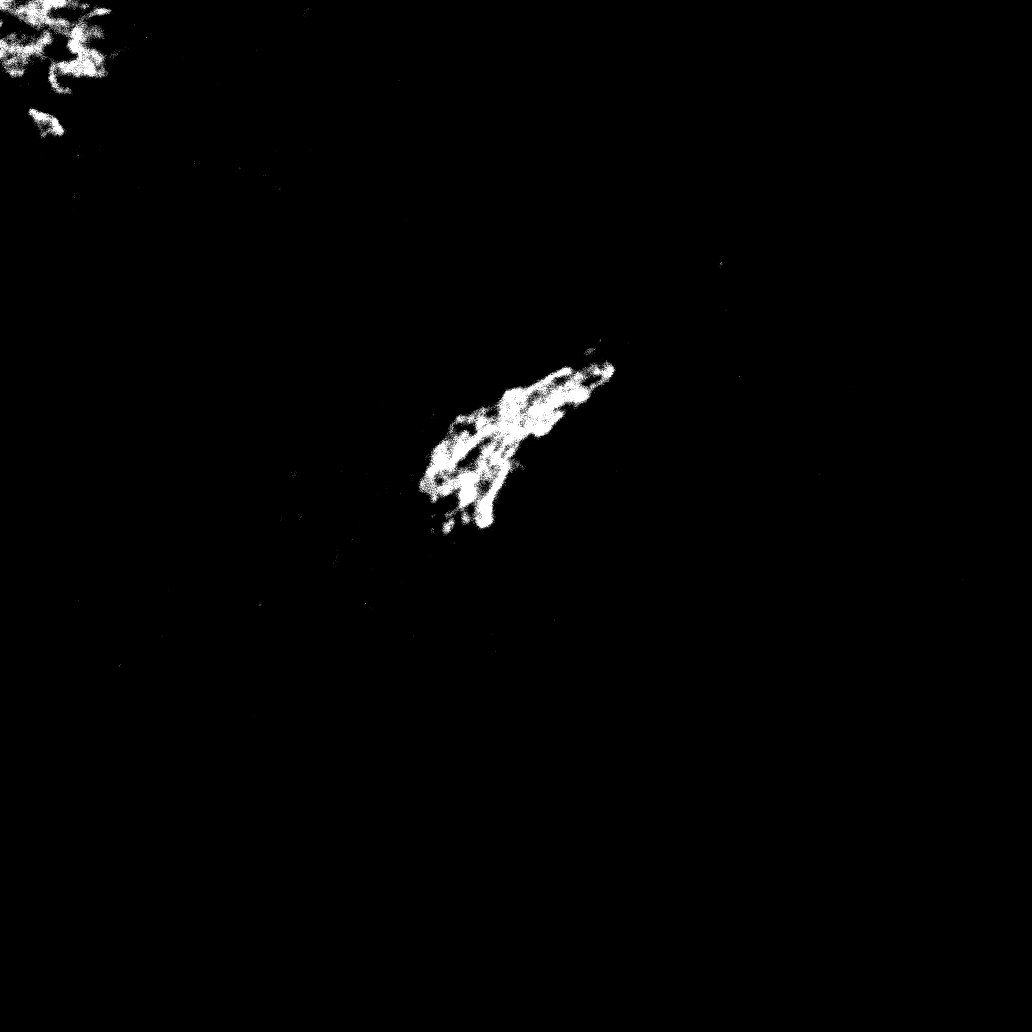

Supplement: Supplementary file 6 — Source data Fig. 4 [file 44319_2025_423_MOESM6_ESM.zip › Figure 4/4C/R19C_GM130.tif]

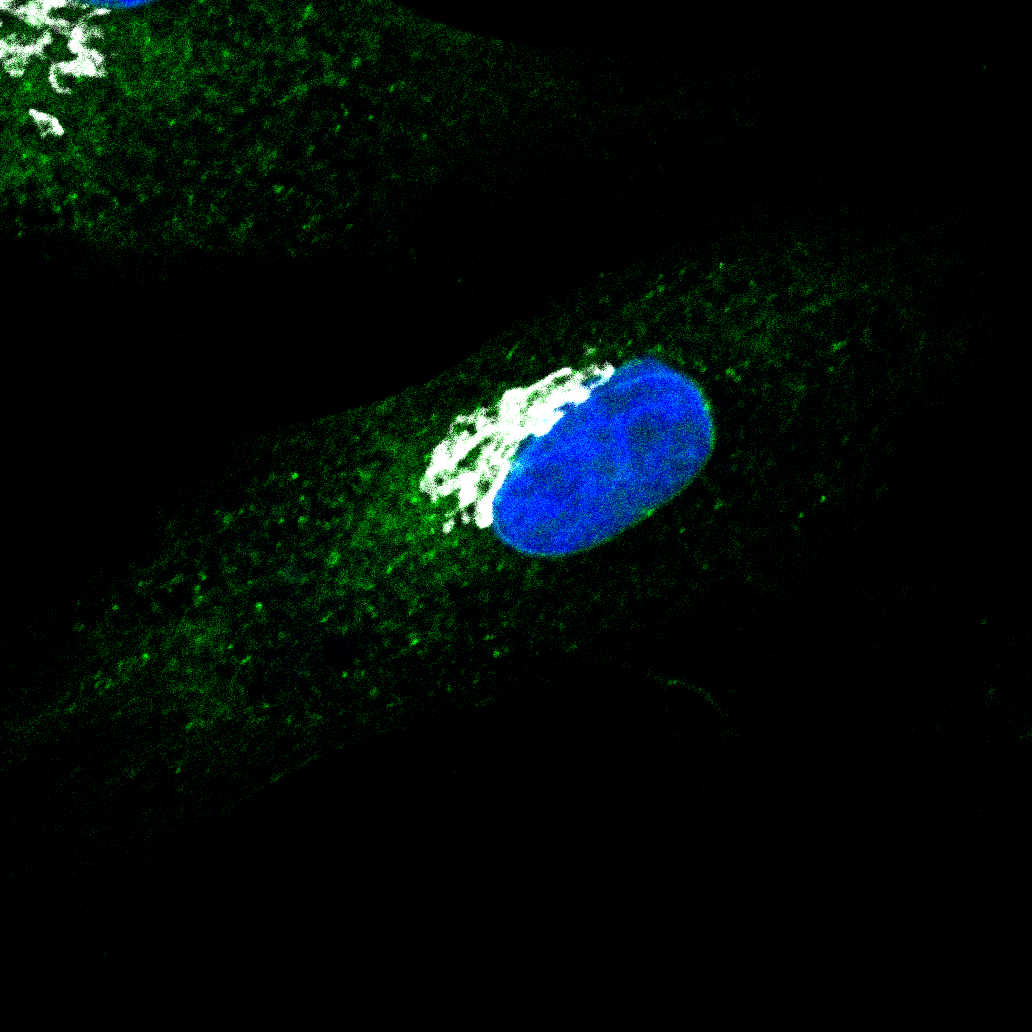

Supplement: Supplementary file 6 — Source data Fig. 4 [file 44319_2025_423_MOESM6_ESM.zip › Figure 4/4C/R19C_merge.tif]

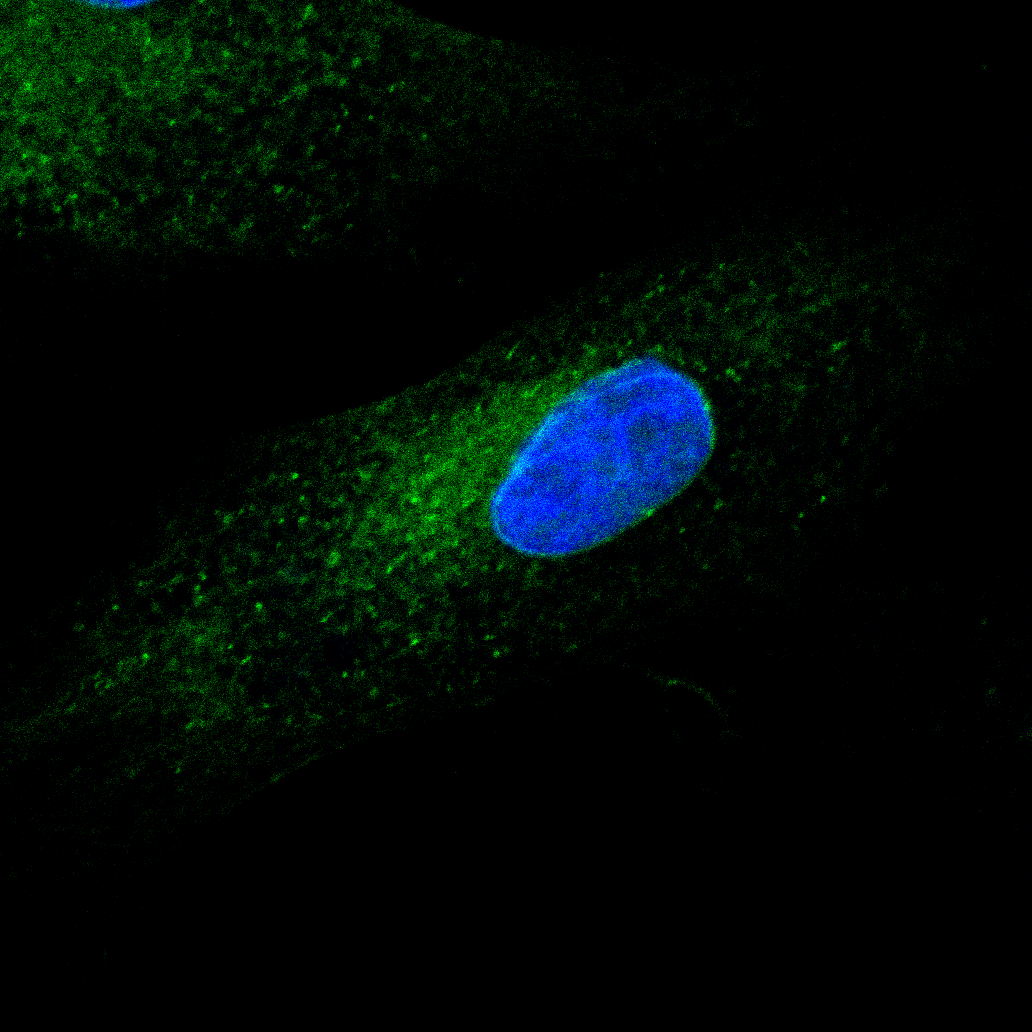

Supplement: Supplementary file 6 — Source data Fig. 4 [file 44319_2025_423_MOESM6_ESM.zip › Figure 4/4C/R19C_STING+DAPI.tif]

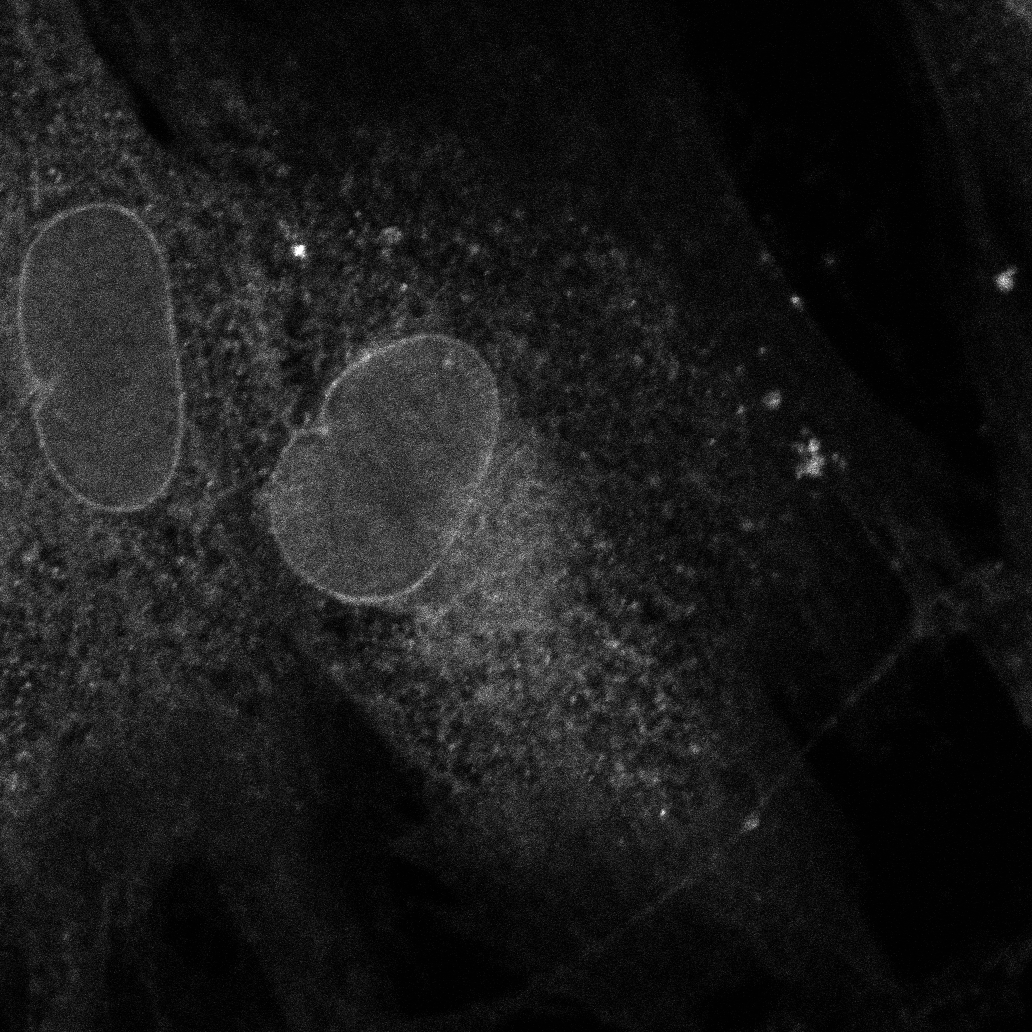

Supplement: Supplementary file 6 — Source data Fig. 4 [file 44319_2025_423_MOESM6_ESM.zip › Figure 4/4C/R99C.tif]

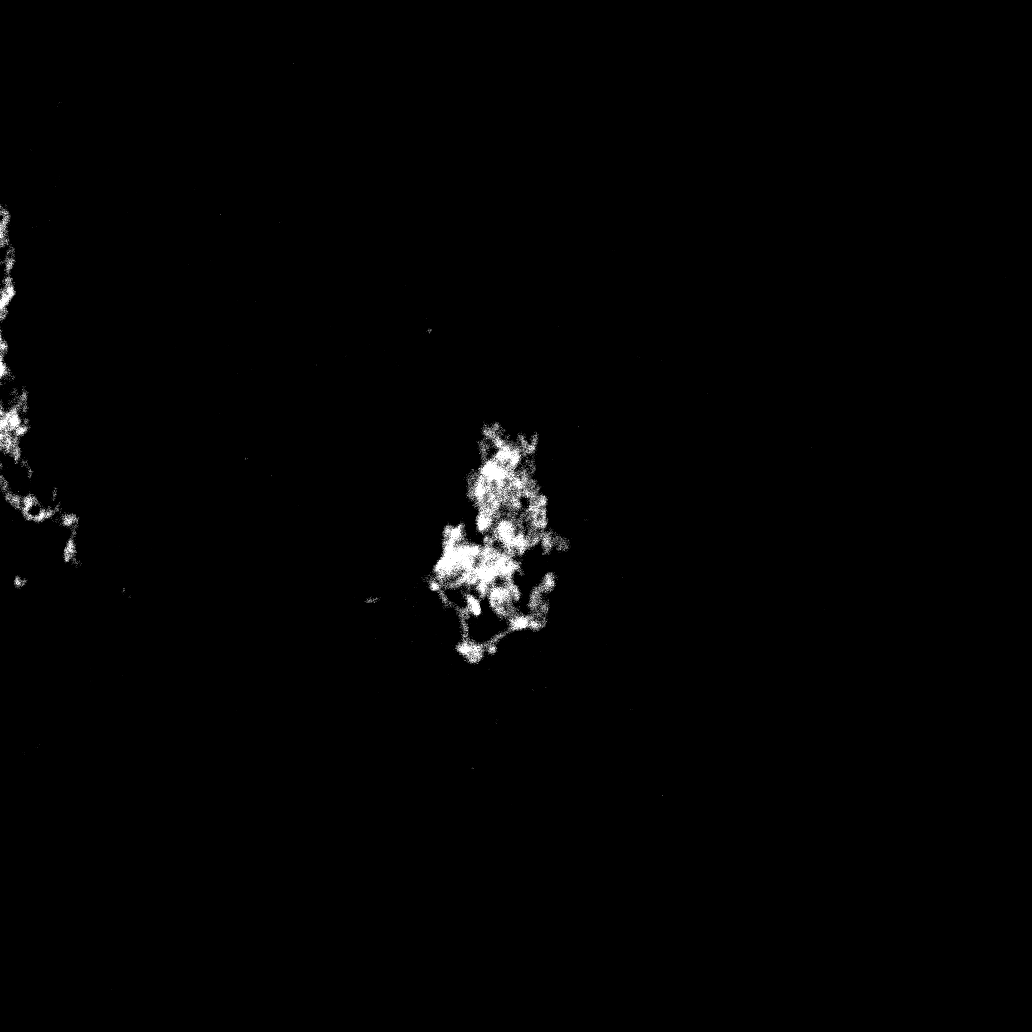

Supplement: Supplementary file 6 — Source data Fig. 4 [file 44319_2025_423_MOESM6_ESM.zip › Figure 4/4C/R99C_GM130.tif]

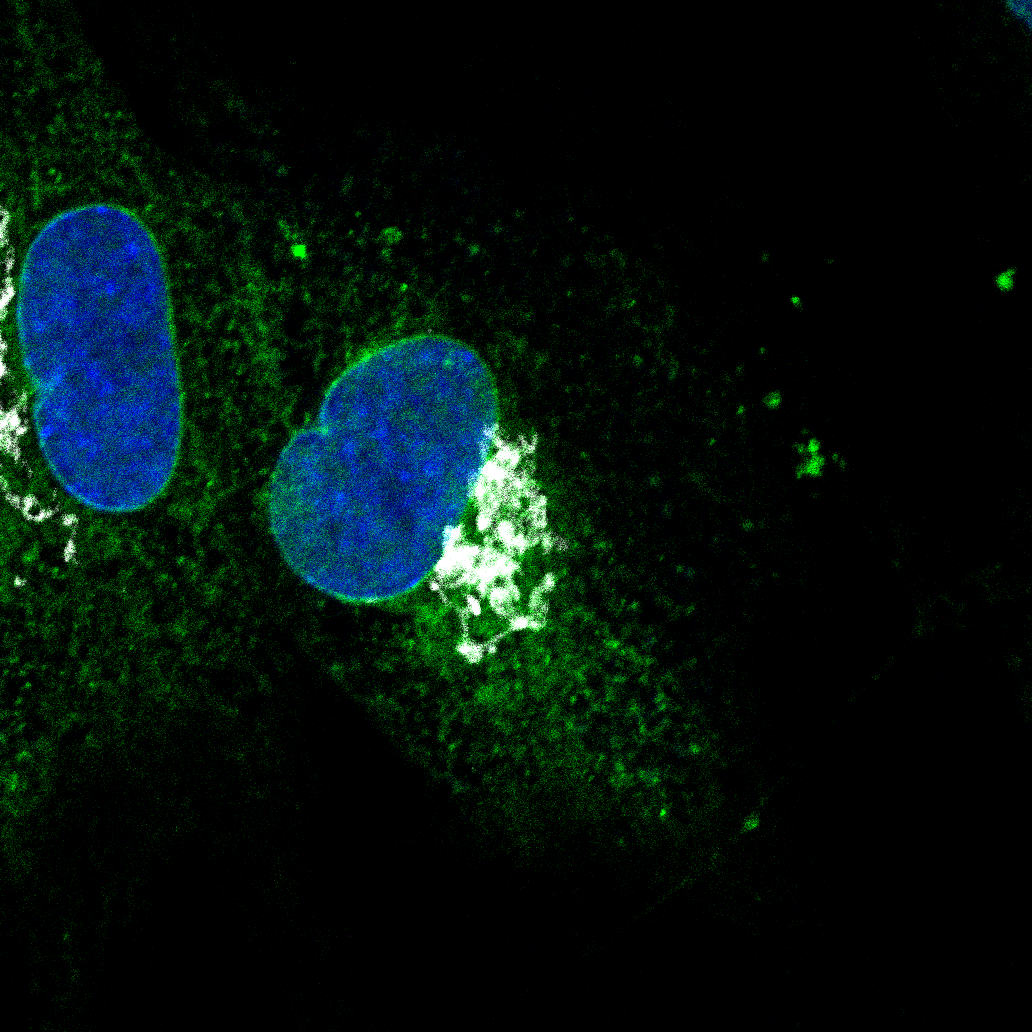

Supplement: Supplementary file 6 — Source data Fig. 4 [file 44319_2025_423_MOESM6_ESM.zip › Figure 4/4C/R99C_merge.tif]

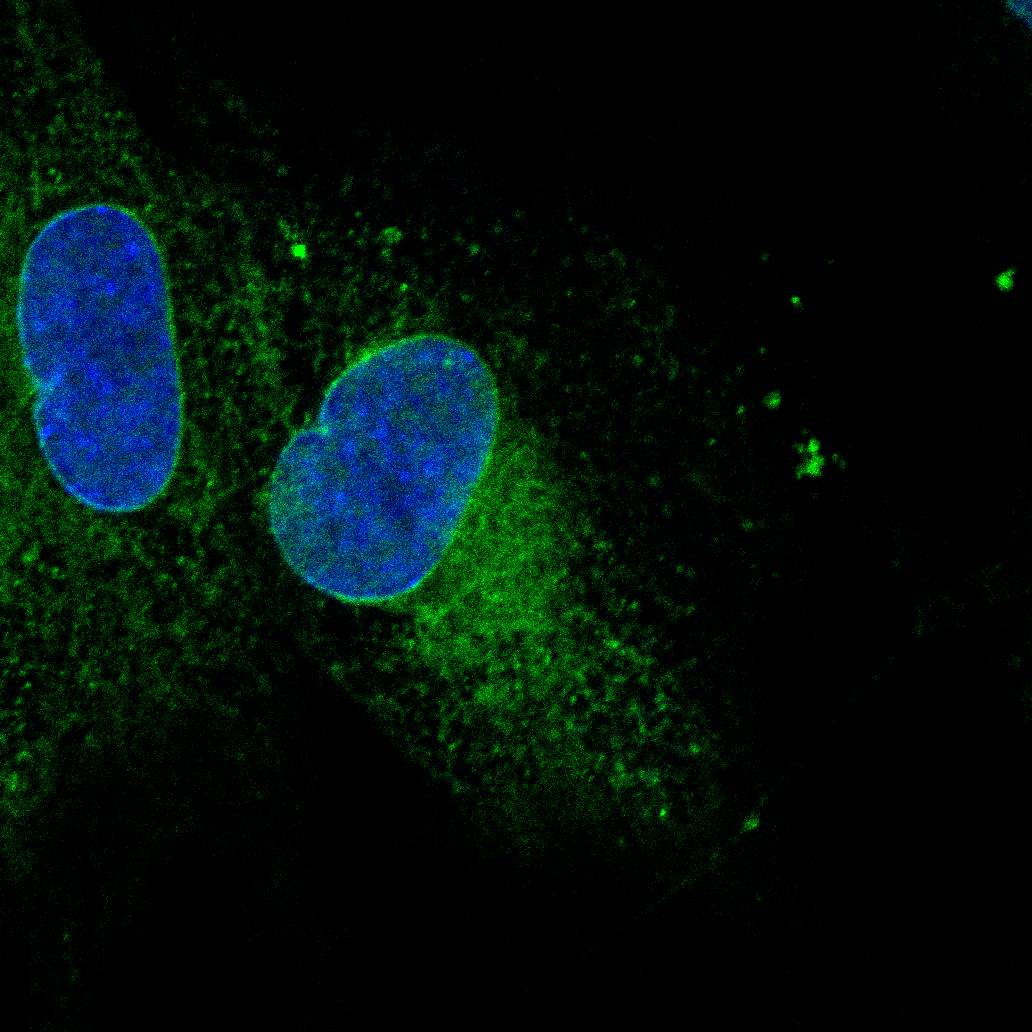

Supplement: Supplementary file 6 — Source data Fig. 4 [file 44319_2025_423_MOESM6_ESM.zip › Figure 4/4C/R99C_STING+DAPI.tif]

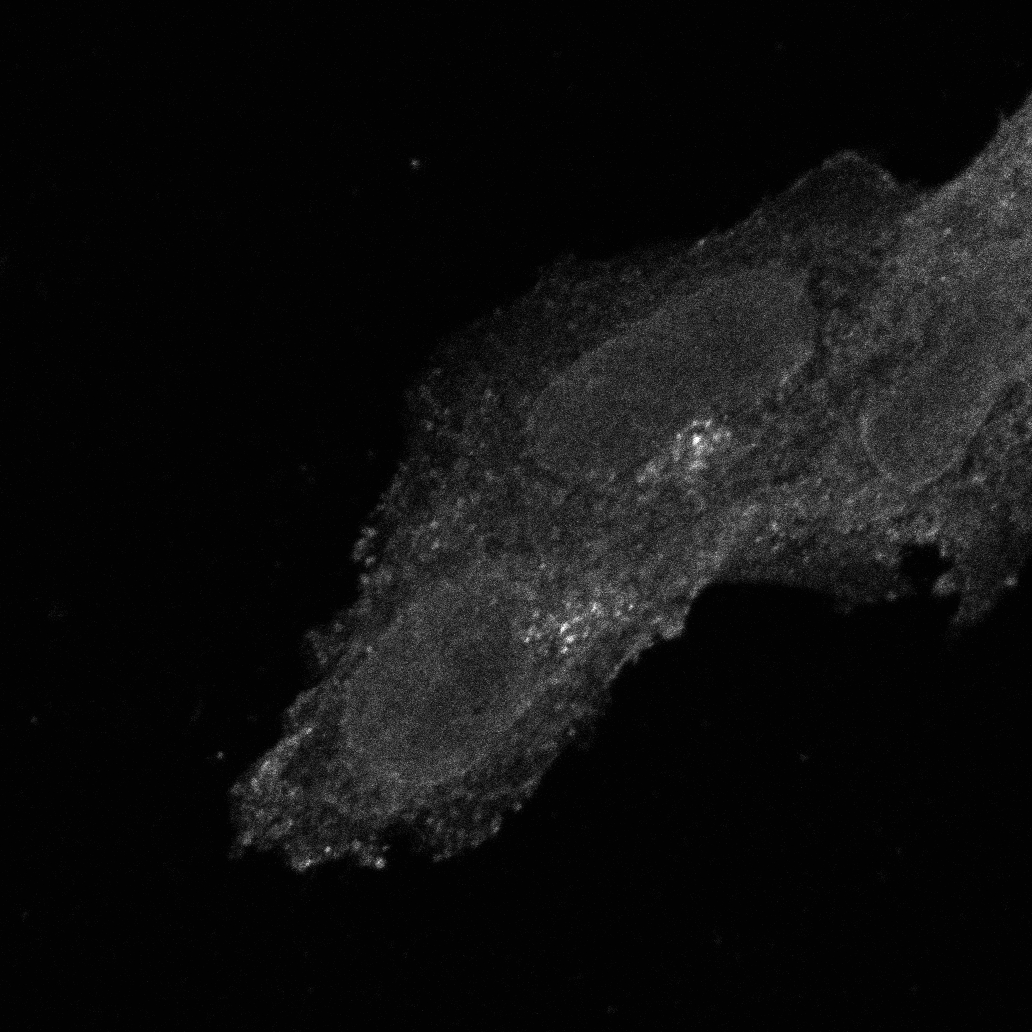

Supplement: Supplementary file 6 — Source data Fig. 4 [file 44319_2025_423_MOESM6_ESM.zip › Figure 4/4C/WT+GAMP.tif]

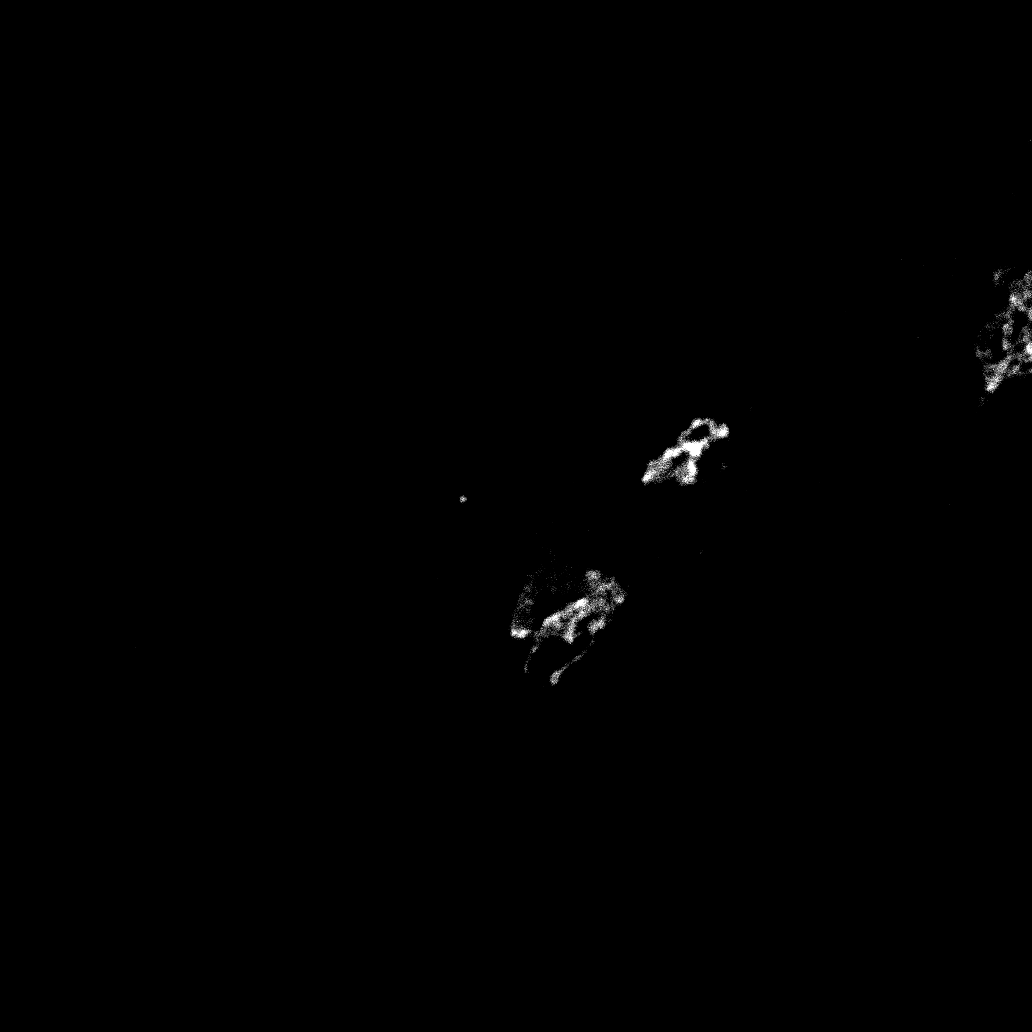

Supplement: Supplementary file 6 — Source data Fig. 4 [file 44319_2025_423_MOESM6_ESM.zip › Figure 4/4C/WT+GAMP_GM130.tif]

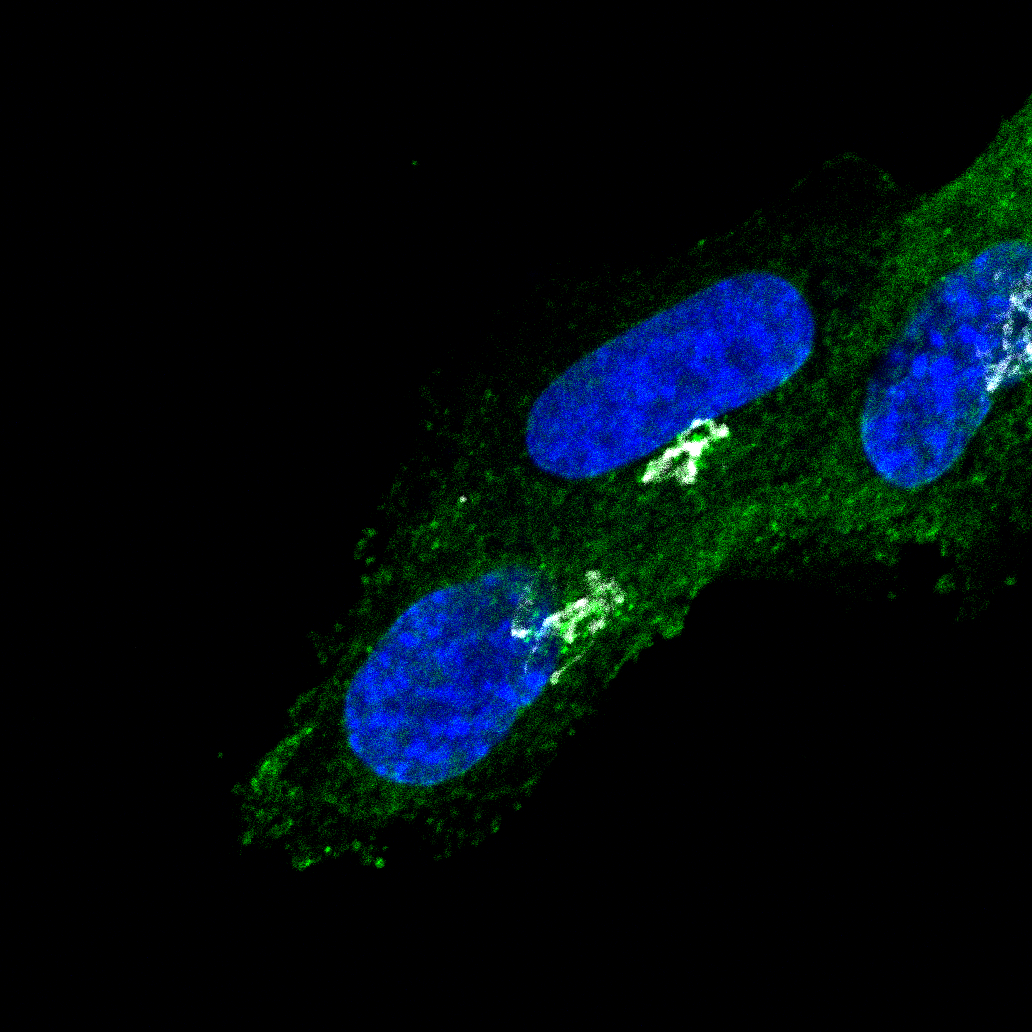

Supplement: Supplementary file 6 — Source data Fig. 4 [file 44319_2025_423_MOESM6_ESM.zip › Figure 4/4C/WT+GAMP_merge.tif]

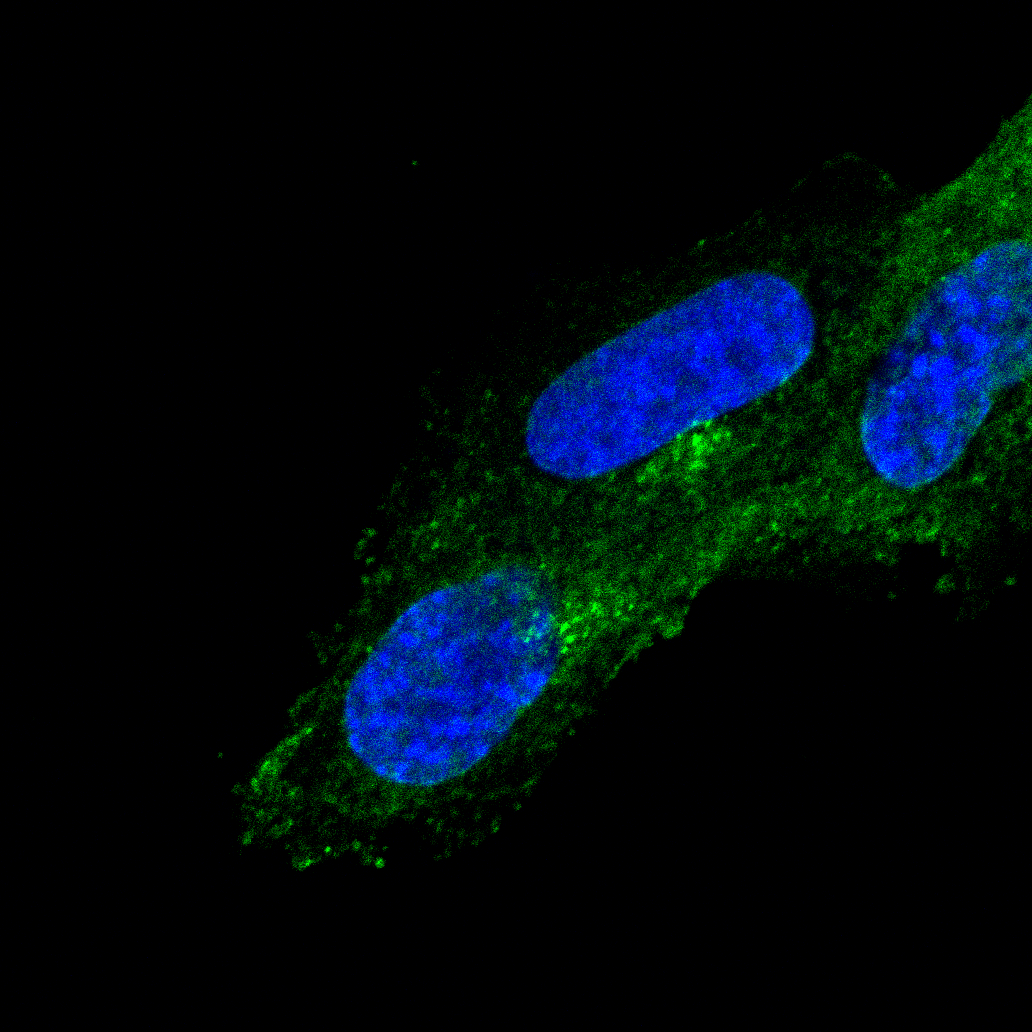

Supplement: Supplementary file 6 — Source data Fig. 4 [file 44319_2025_423_MOESM6_ESM.zip › Figure 4/4C/WT+GAMP_STING+DAPI.tif]

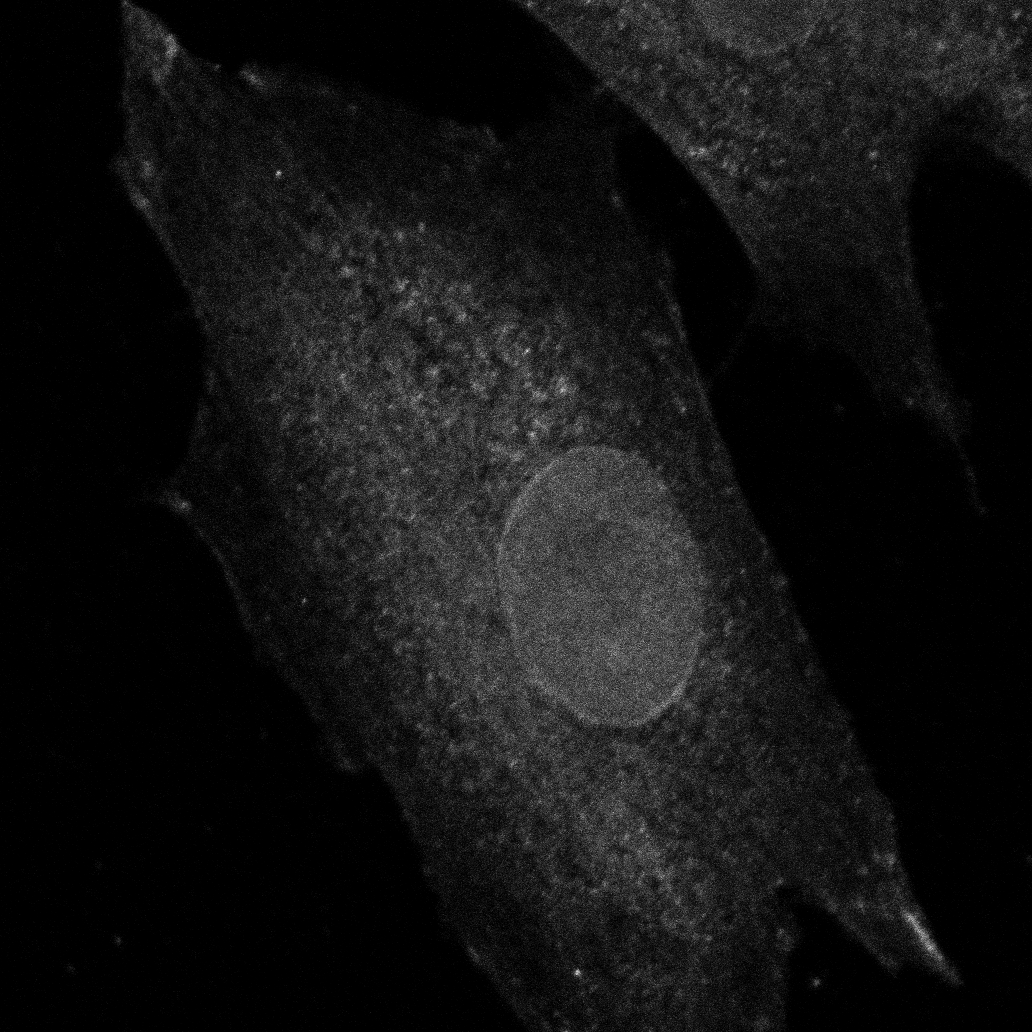

Supplement: Supplementary file 6 — Source data Fig. 4 [file 44319_2025_423_MOESM6_ESM.zip › Figure 4/4C/WT.tif]

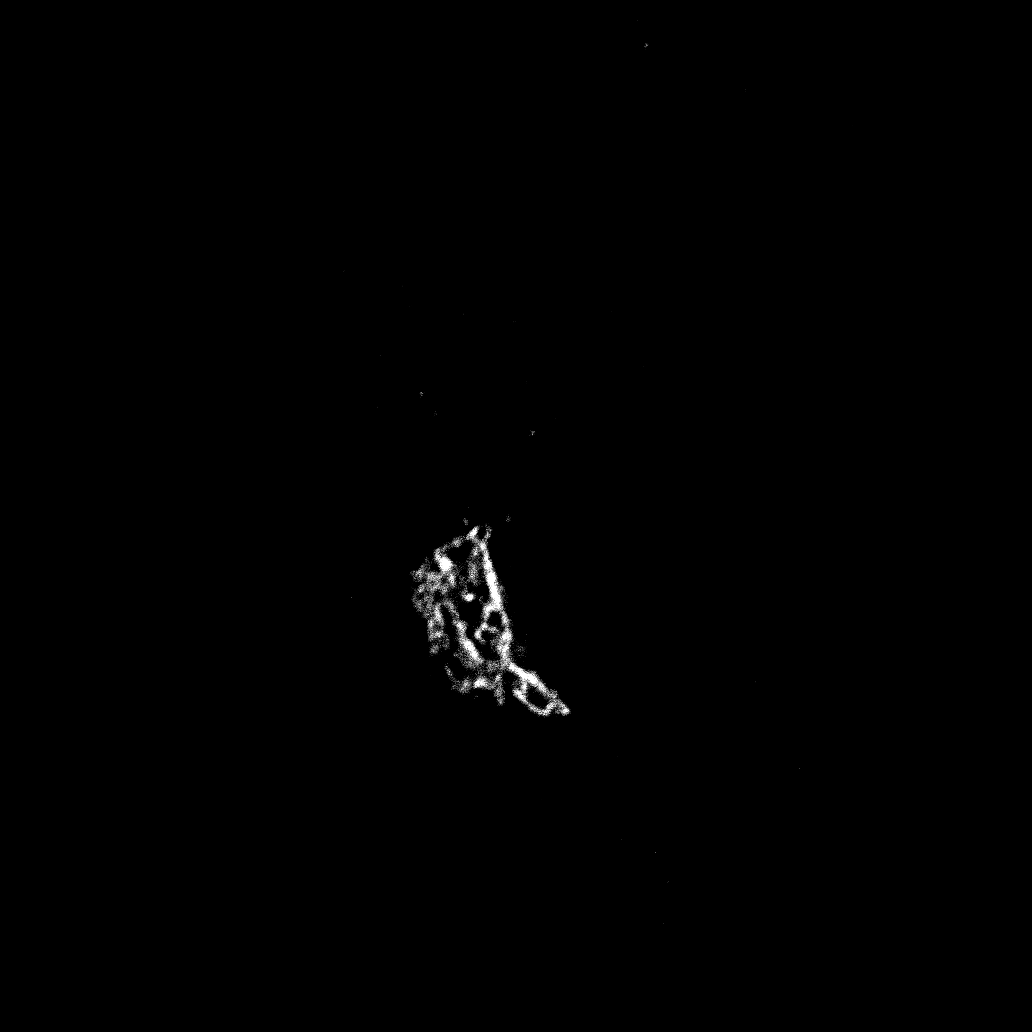

Supplement: Supplementary file 6 — Source data Fig. 4 [file 44319_2025_423_MOESM6_ESM.zip › Figure 4/4C/WT_GM130.tif]

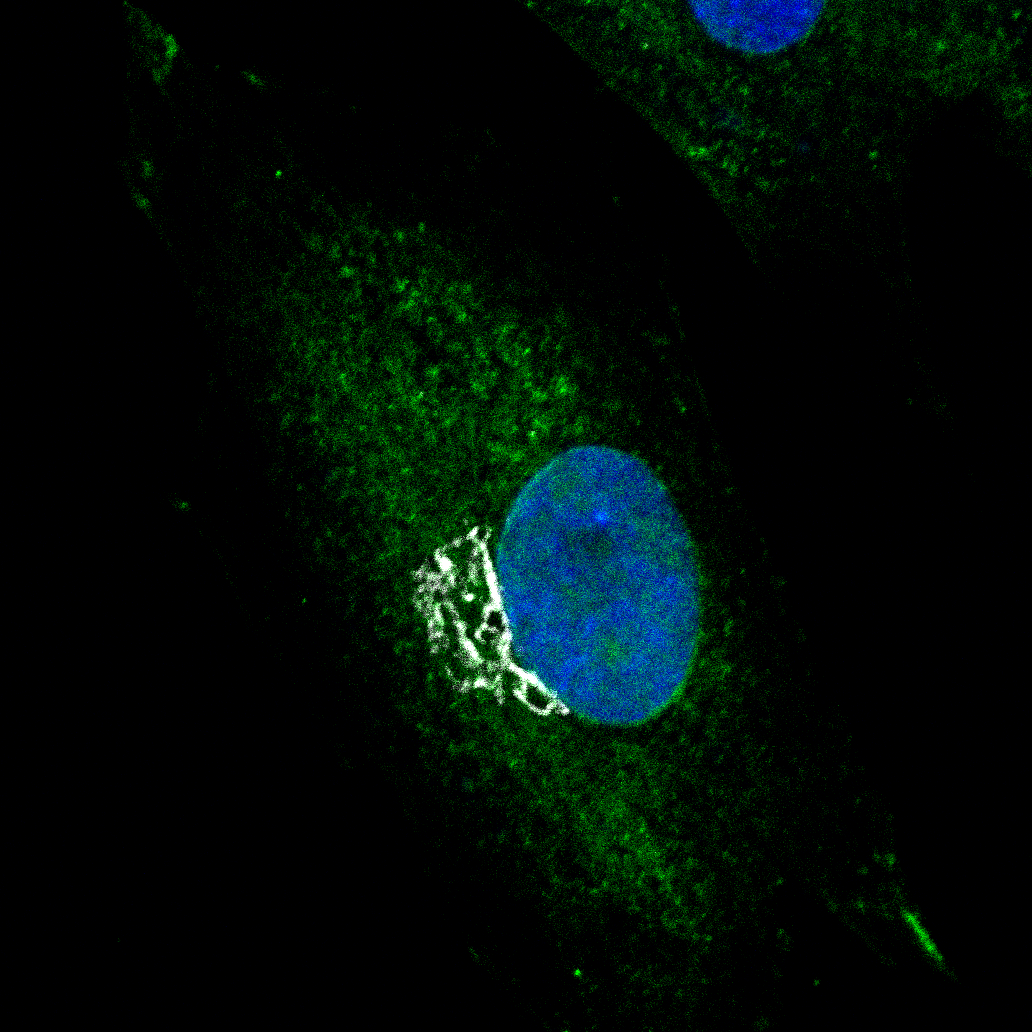

Supplement: Supplementary file 6 — Source data Fig. 4 [file 44319_2025_423_MOESM6_ESM.zip › Figure 4/4C/WT_merge.tif]

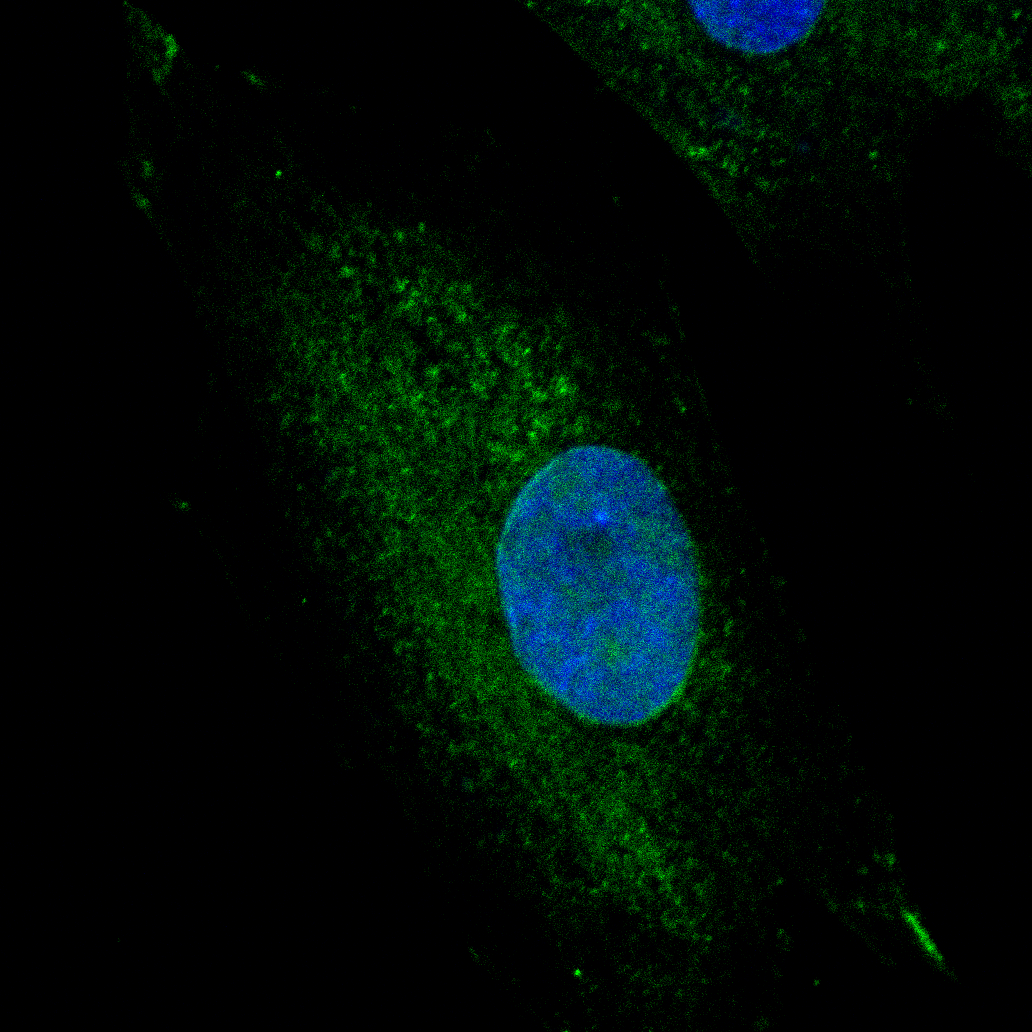

Supplement: Supplementary file 6 — Source data Fig. 4 [file 44319_2025_423_MOESM6_ESM.zip › Figure 4/4C/WT_STING+DAPI.tif]

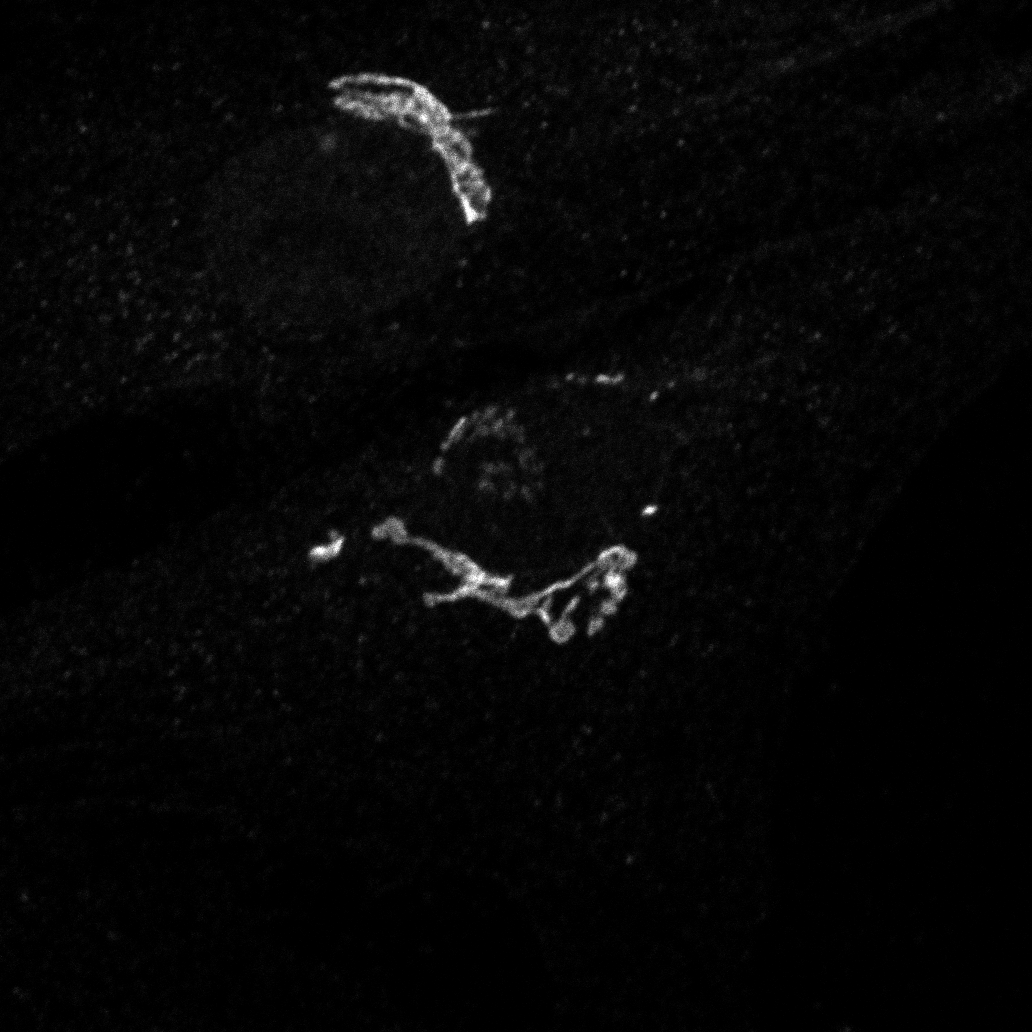

Supplement: Supplementary file 6 — Source data Fig. 4 [file 44319_2025_423_MOESM6_ESM.zip › Figure 4/4E/R19C.tif]

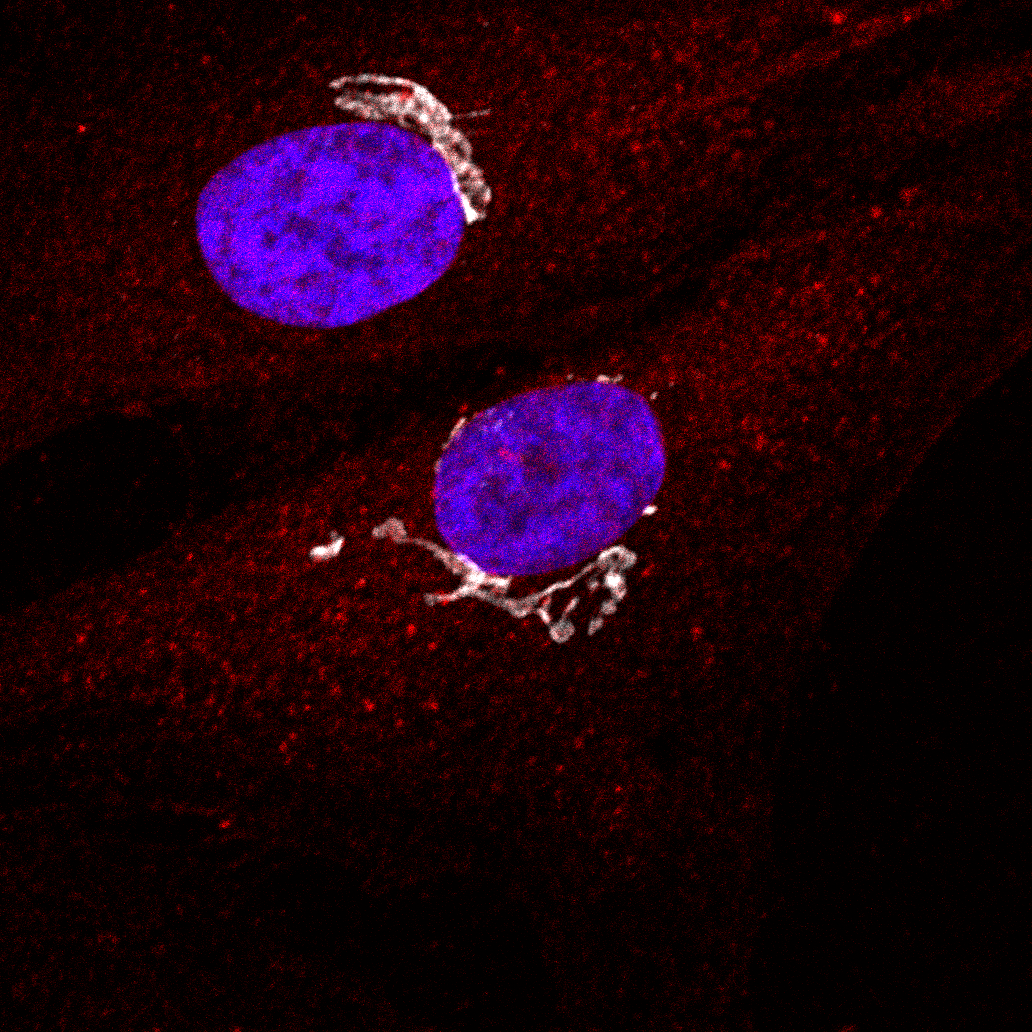

Supplement: Supplementary file 6 — Source data Fig. 4 [file 44319_2025_423_MOESM6_ESM.zip › Figure 4/4E/R19C_pTBK1+DAPI+GM130.tif]

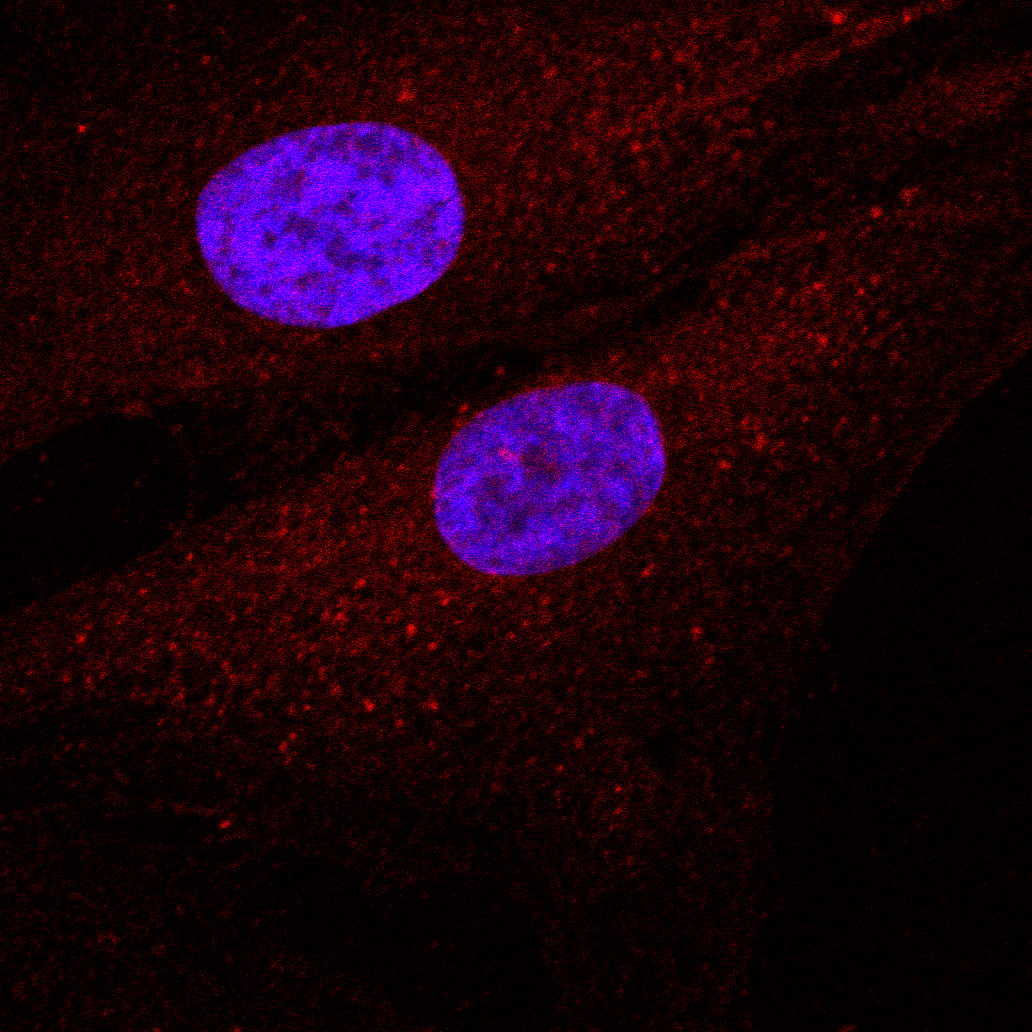

Supplement: Supplementary file 6 — Source data Fig. 4 [file 44319_2025_423_MOESM6_ESM.zip › Figure 4/4E/R19C_pTBK1+DAPI.tif]

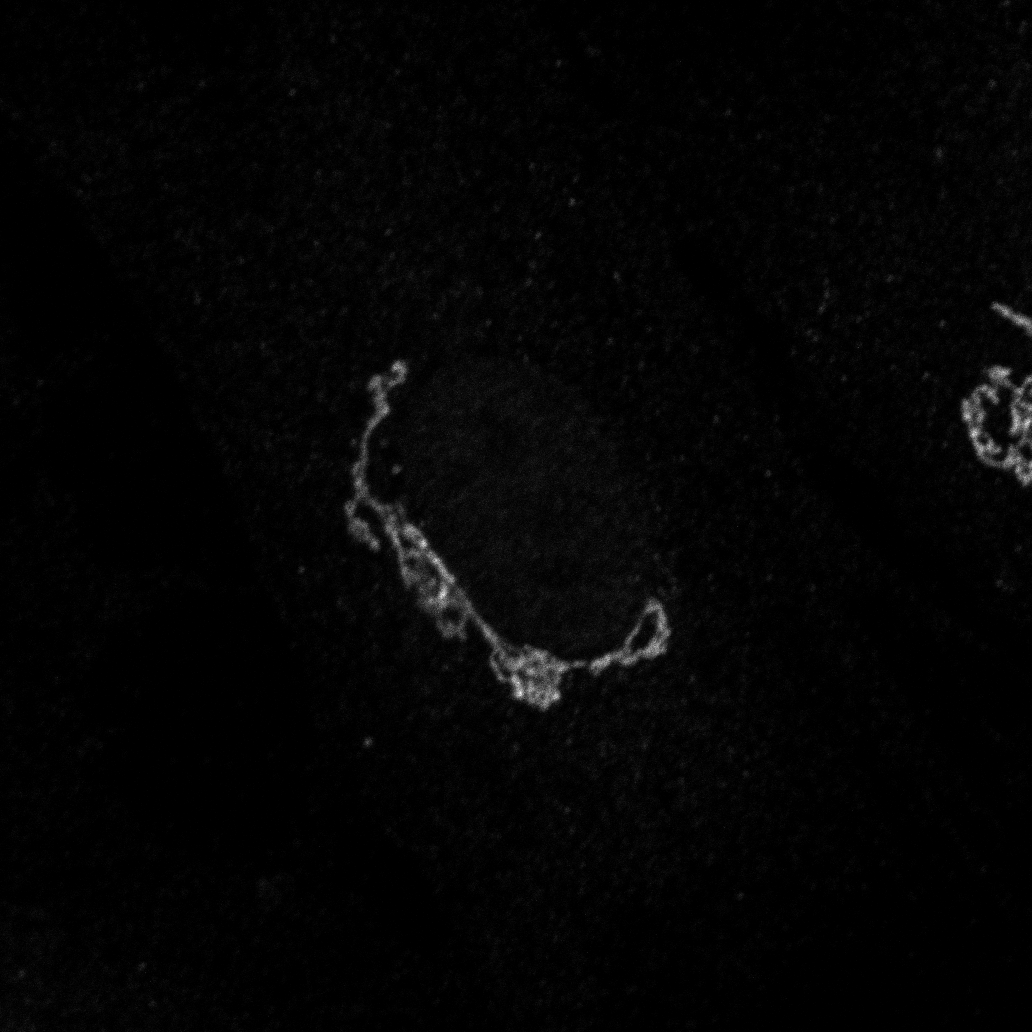

Supplement: Supplementary file 6 — Source data Fig. 4 [file 44319_2025_423_MOESM6_ESM.zip › Figure 4/4E/WT.tif]

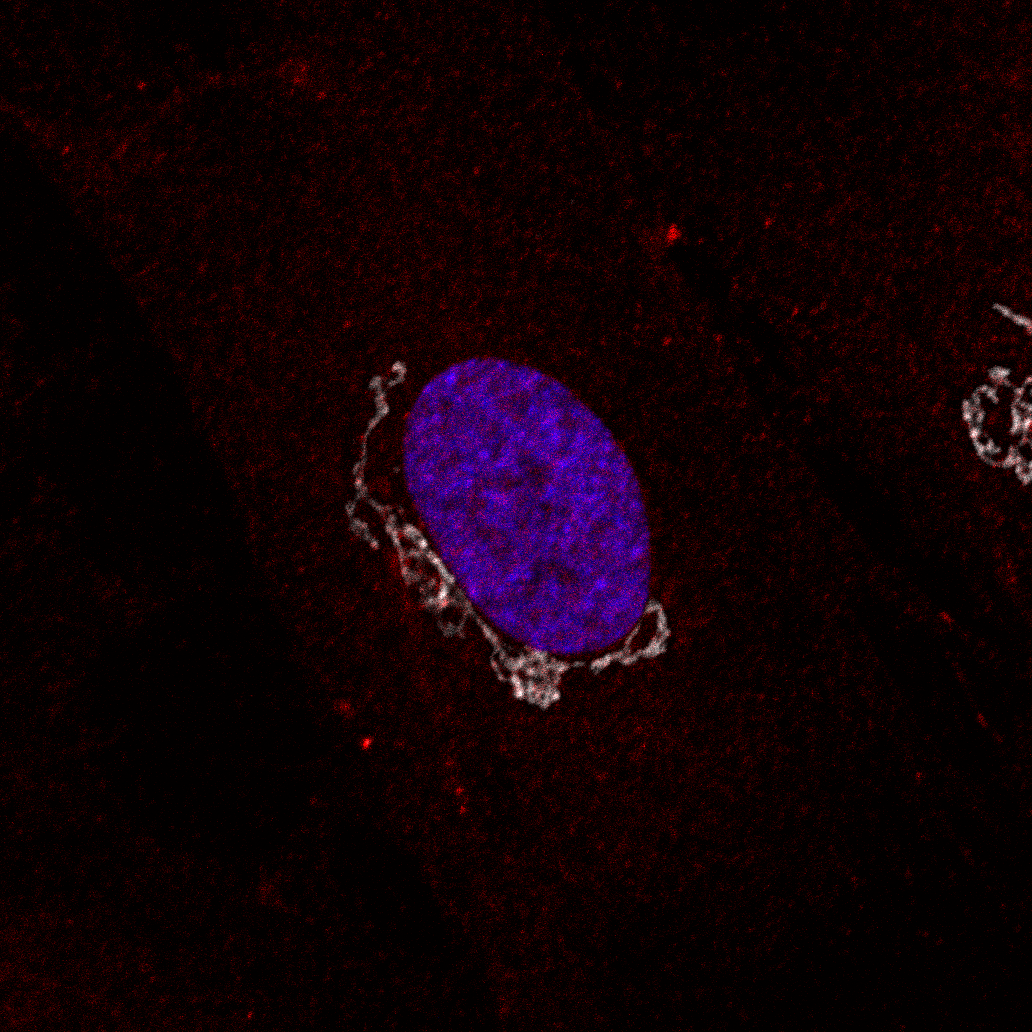

Supplement: Supplementary file 6 — Source data Fig. 4 [file 44319_2025_423_MOESM6_ESM.zip › Figure 4/4E/WT_pTBK1+DAPI+GM130.tif]

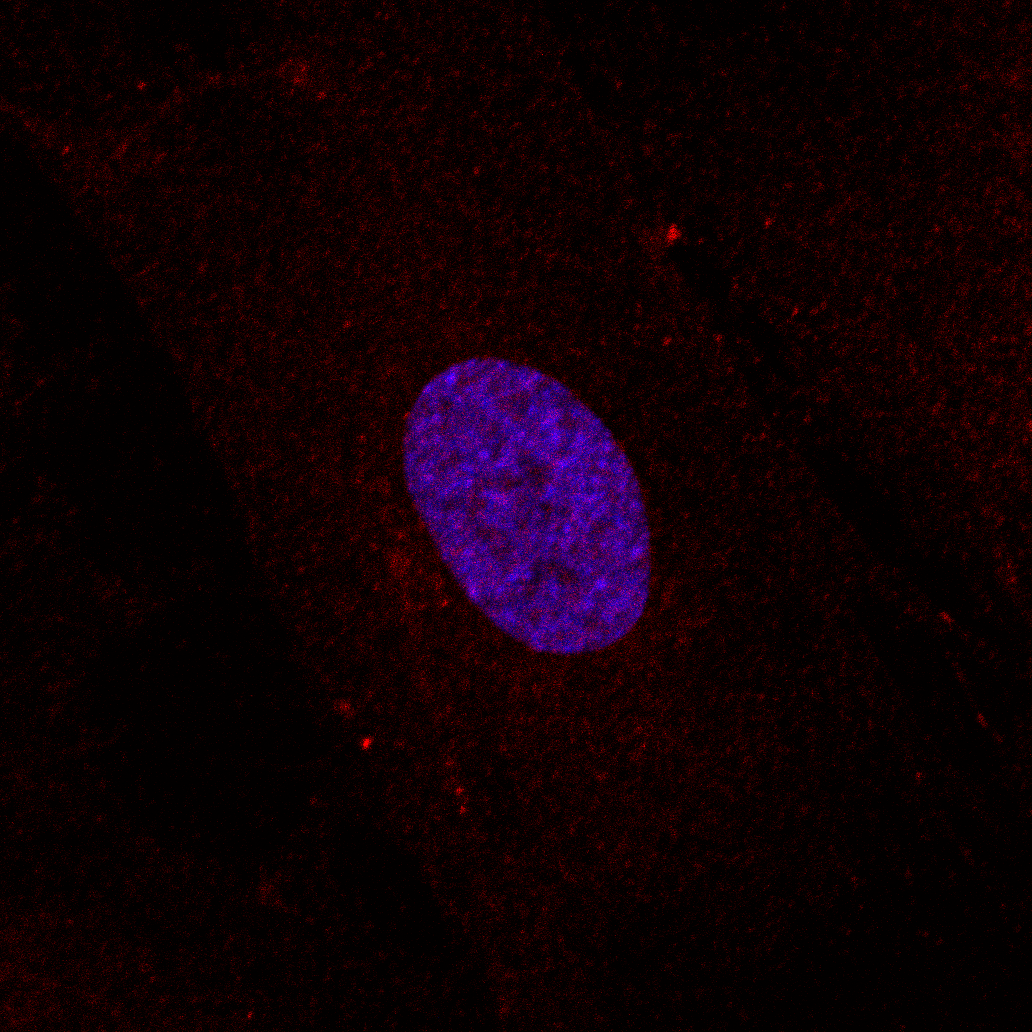

Supplement: Supplementary file 6 — Source data Fig. 4 [file 44319_2025_423_MOESM6_ESM.zip › Figure 4/4E/WT_pTBK1+DAPI.tif]

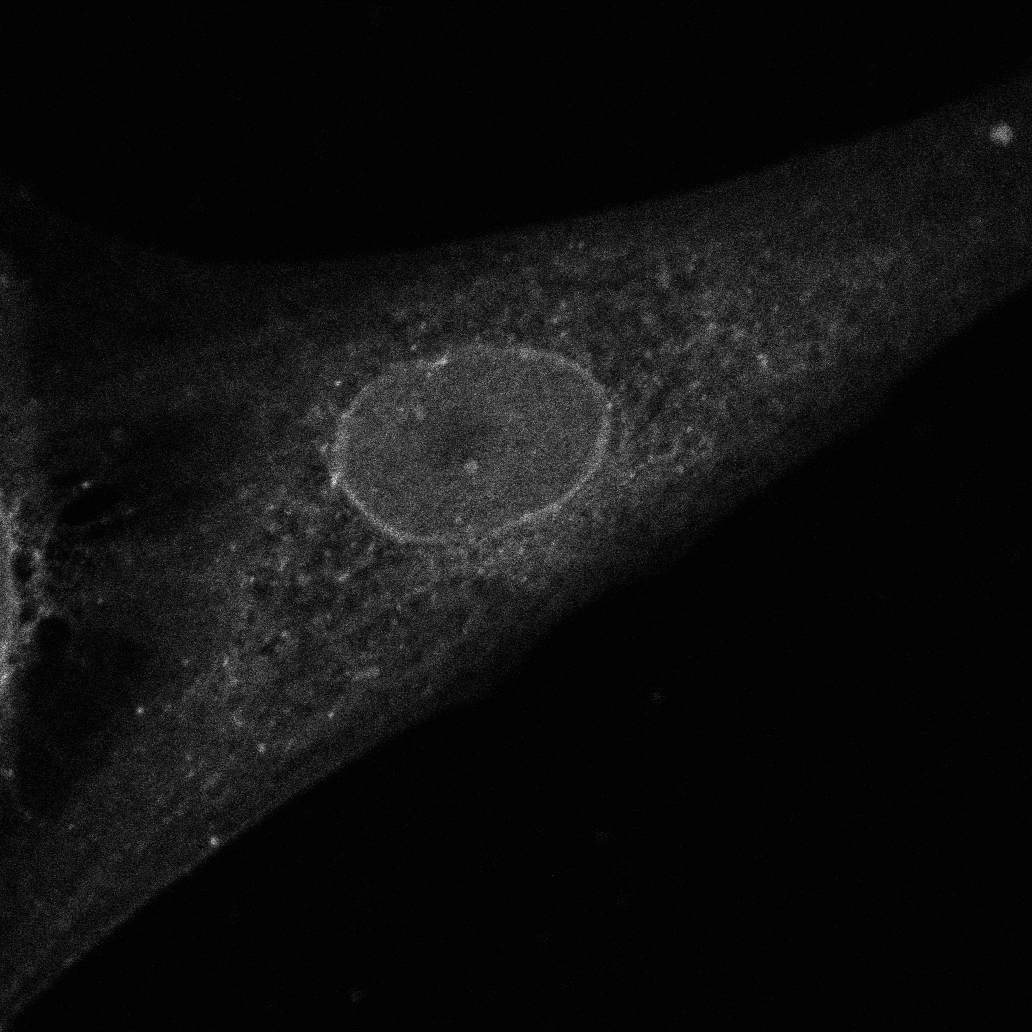

Supplement: Supplementary file 7 — Source data Fig. 5 [file 44319_2025_423_MOESM7_ESM.zip › Figure 5/5A/ARF1 R19C_24h cGAMP.tif]

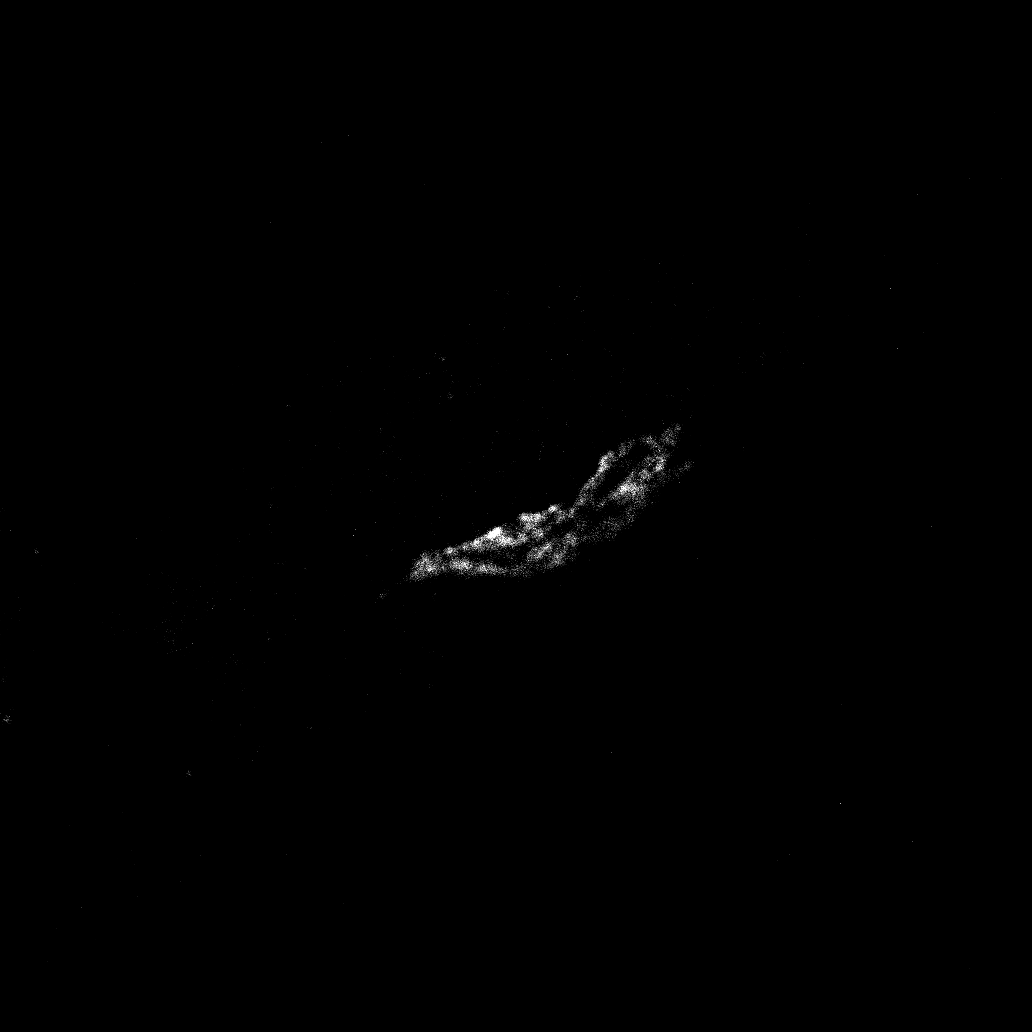

Supplement: Supplementary file 7 — Source data Fig. 5 [file 44319_2025_423_MOESM7_ESM.zip › Figure 5/5A/ARF1 R19C_24h cGAMP_GM130.tif]

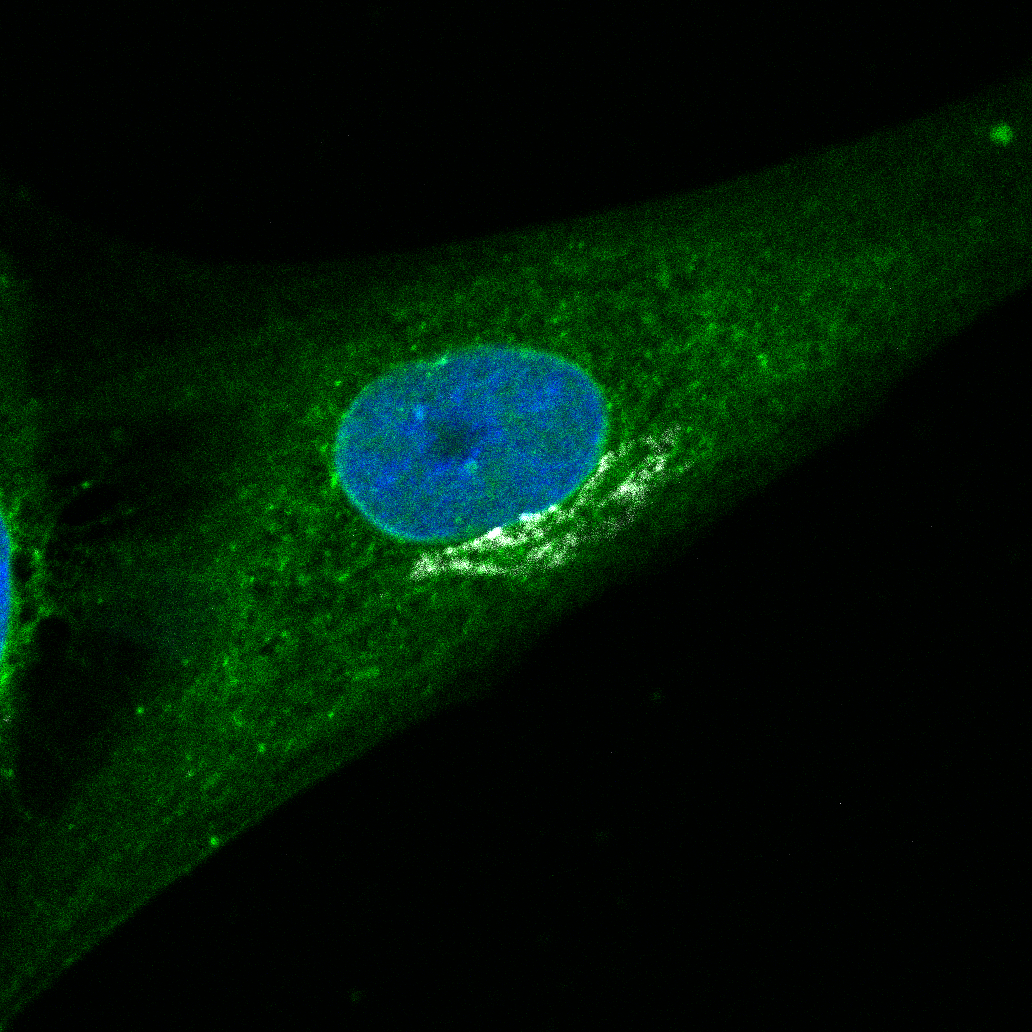

Supplement: Supplementary file 7 — Source data Fig. 5 [file 44319_2025_423_MOESM7_ESM.zip › Figure 5/5A/ARF1 R19C_24h cGAMP_merge.tif]

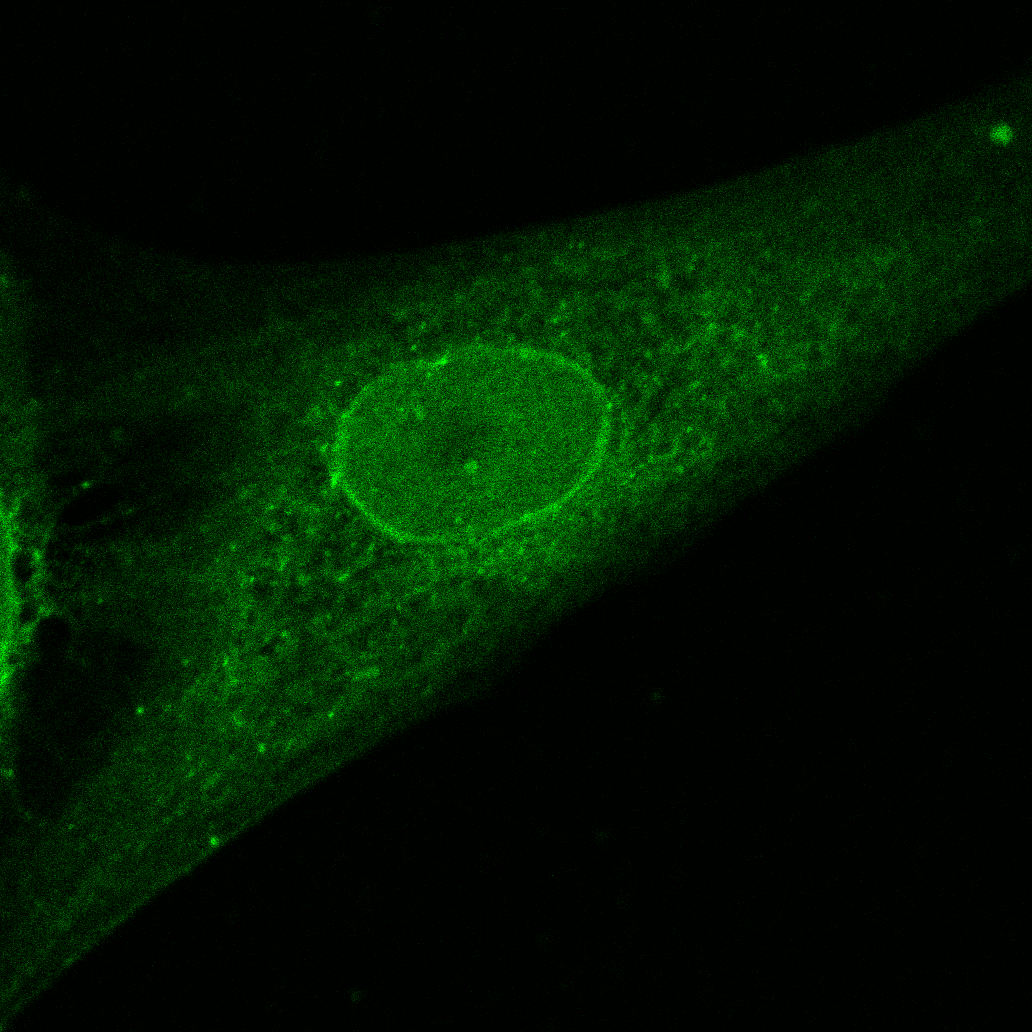

Supplement: Supplementary file 7 — Source data Fig. 5 [file 44319_2025_423_MOESM7_ESM.zip › Figure 5/5A/ARF1 R19C_24h cGAMP_STING.tif]

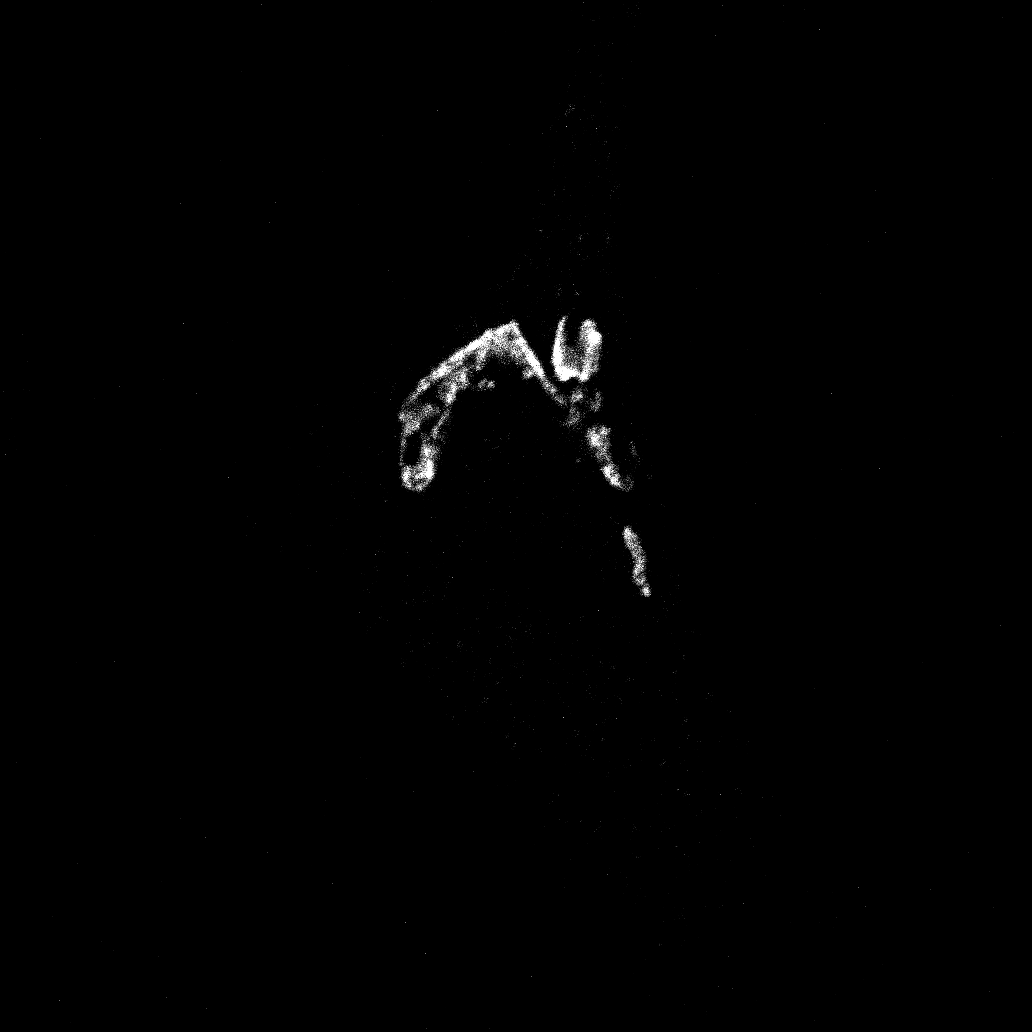

Supplement: Supplementary file 7 — Source data Fig. 5 [file 44319_2025_423_MOESM7_ESM.zip › Figure 5/5A/ARF1 R19C_4h cGAMP_GM130.tif]

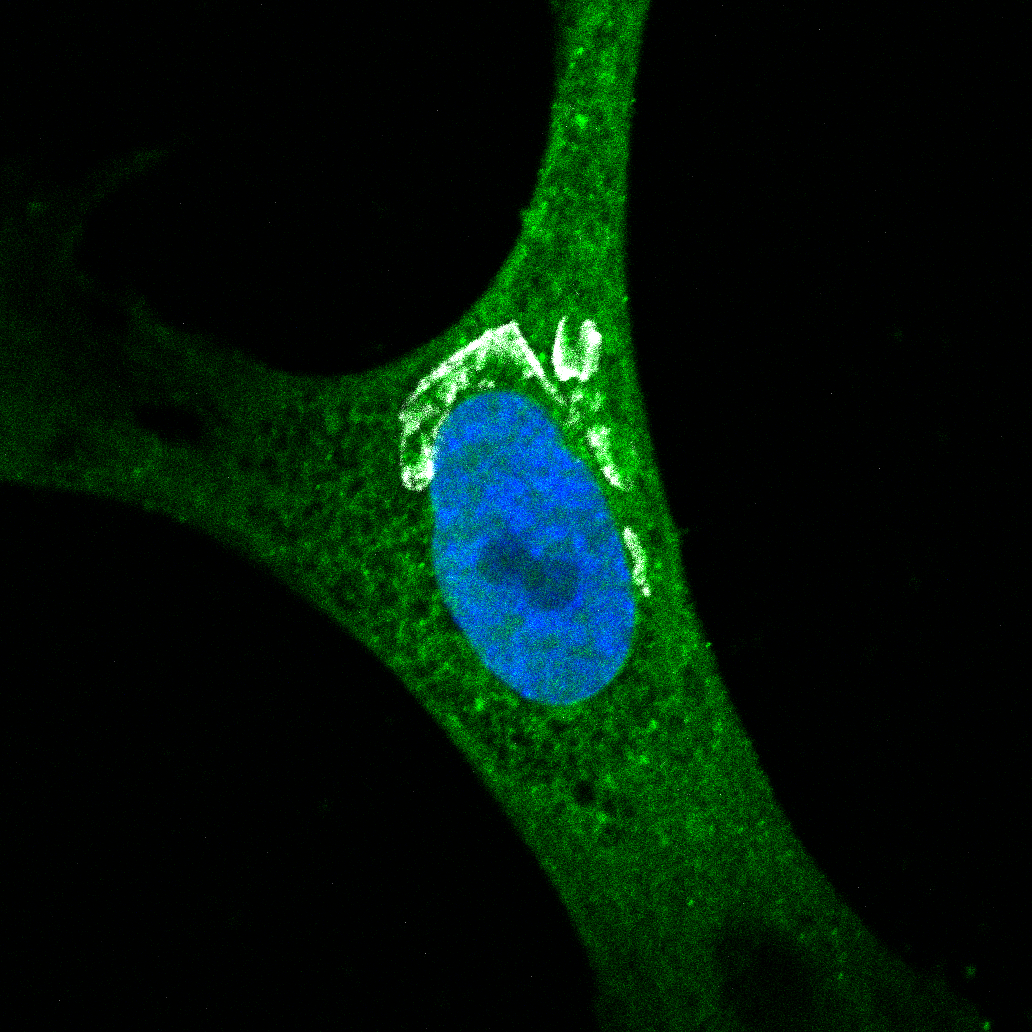

Supplement: Supplementary file 7 — Source data Fig. 5 [file 44319_2025_423_MOESM7_ESM.zip › Figure 5/5A/ARF1 R19C_4h cGAMP_merge.tif]

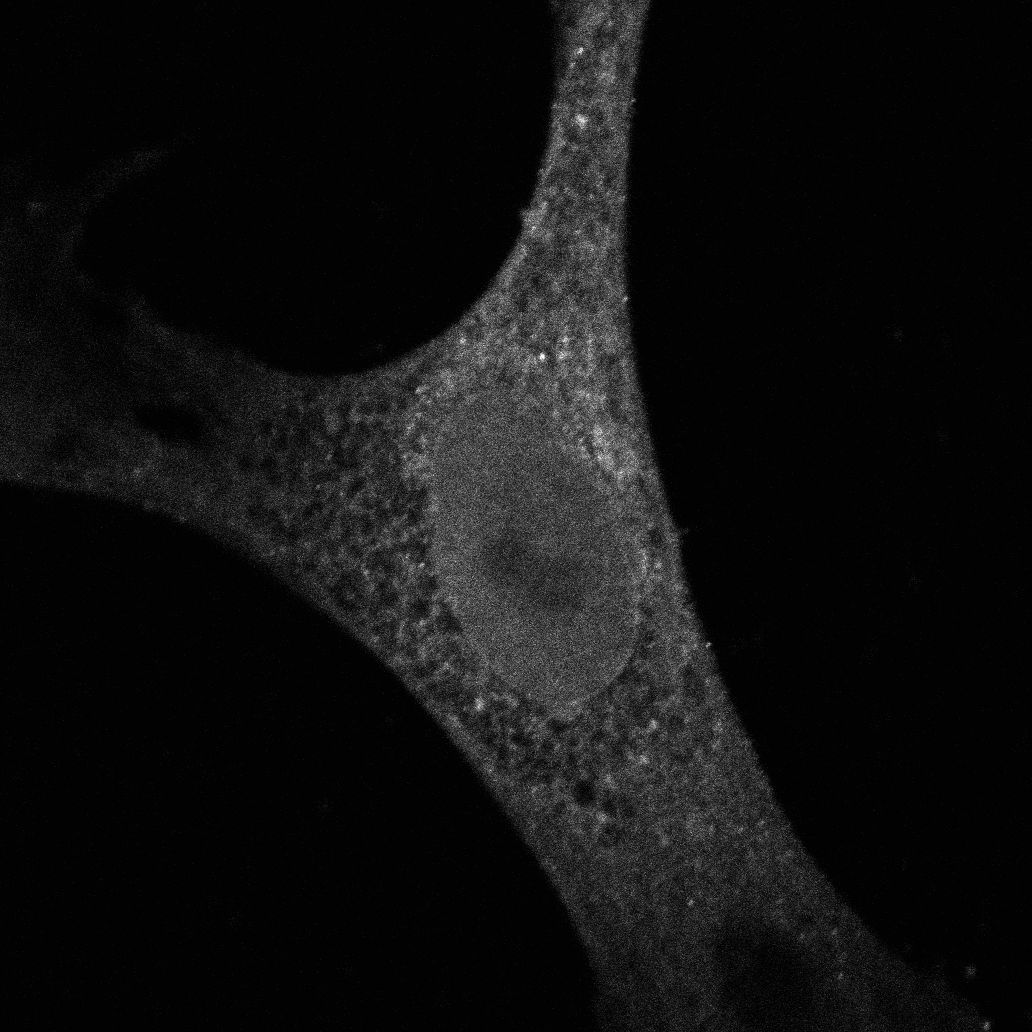

Supplement: Supplementary file 7 — Source data Fig. 5 [file 44319_2025_423_MOESM7_ESM.zip › Figure 5/5A/ARF1 R19C_4h cGAMP_original.tif]

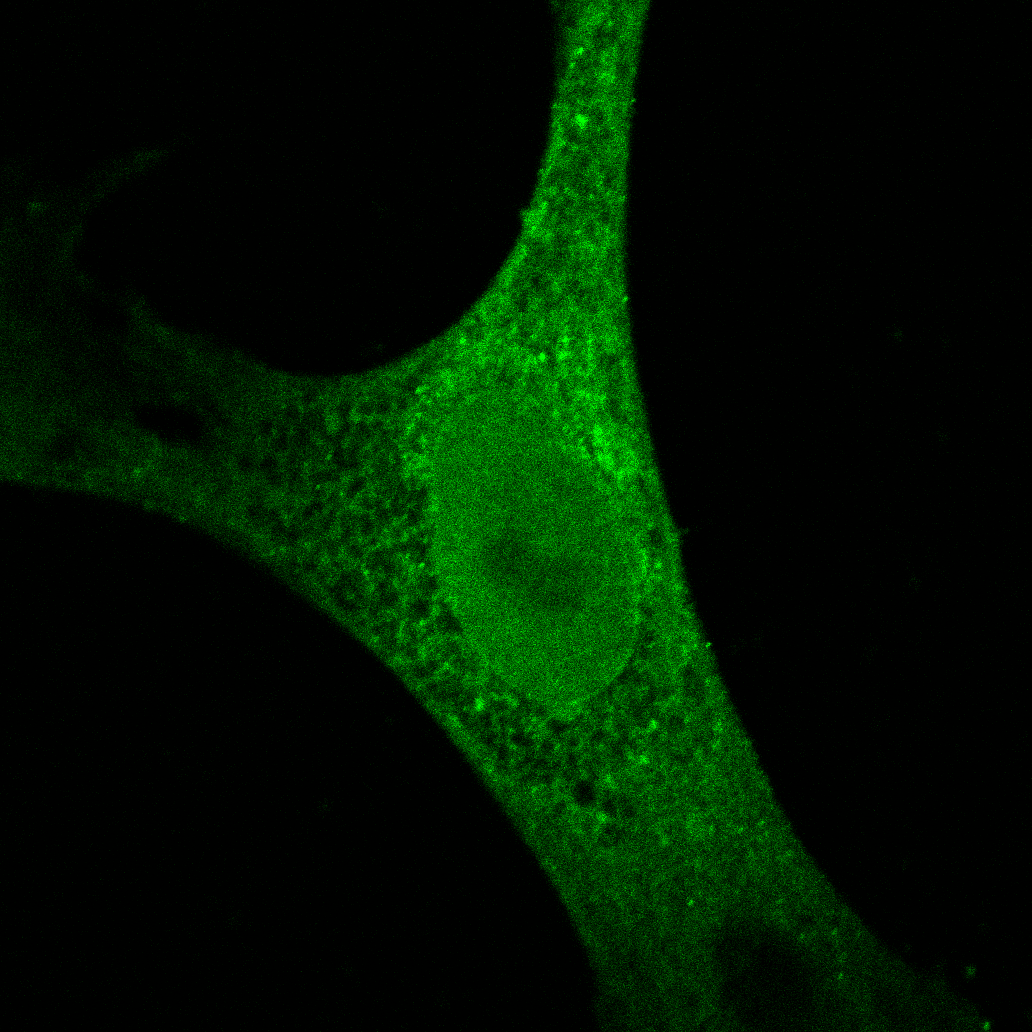

Supplement: Supplementary file 7 — Source data Fig. 5 [file 44319_2025_423_MOESM7_ESM.zip › Figure 5/5A/ARF1 R19C_4h cGAMP_STING.tif]

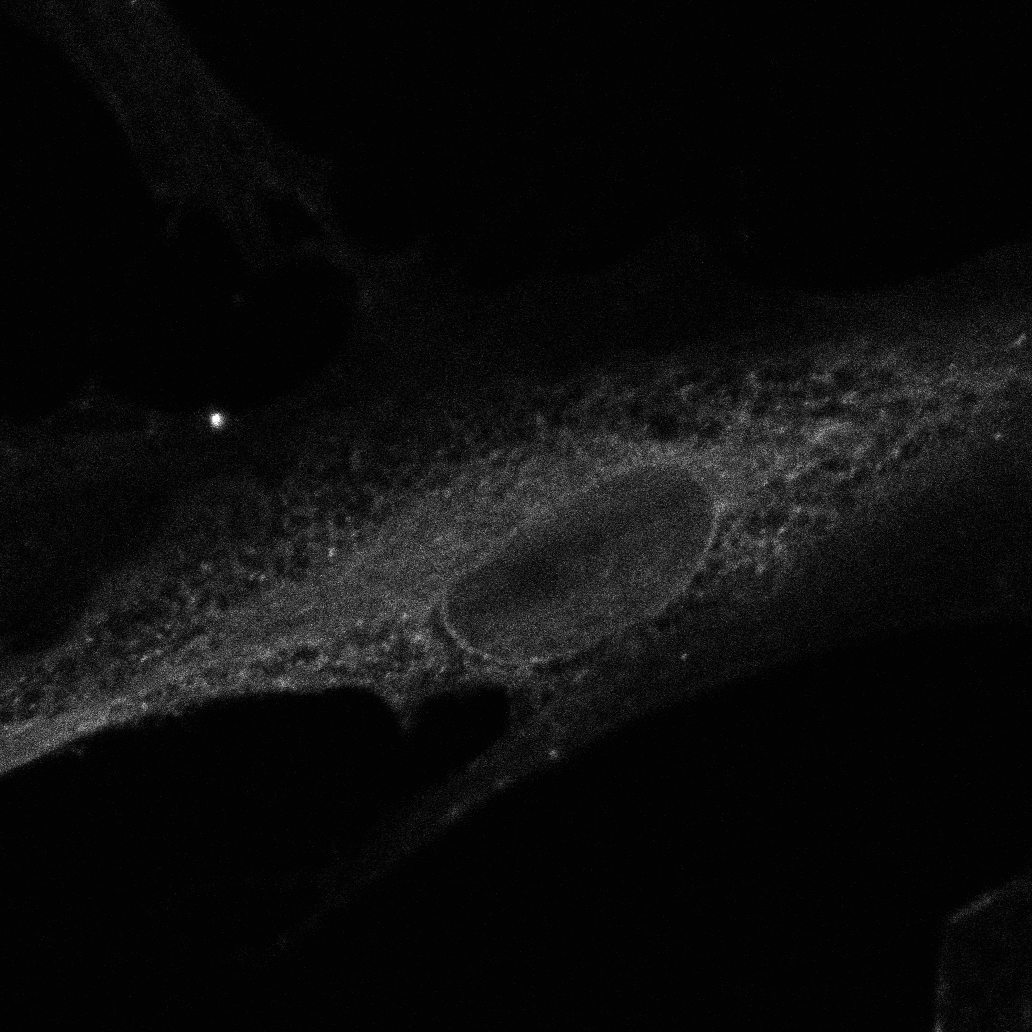

Supplement: Supplementary file 7 — Source data Fig. 5 [file 44319_2025_423_MOESM7_ESM.zip › Figure 5/5A/ARF1 R19C_8h cGAMP.tif]

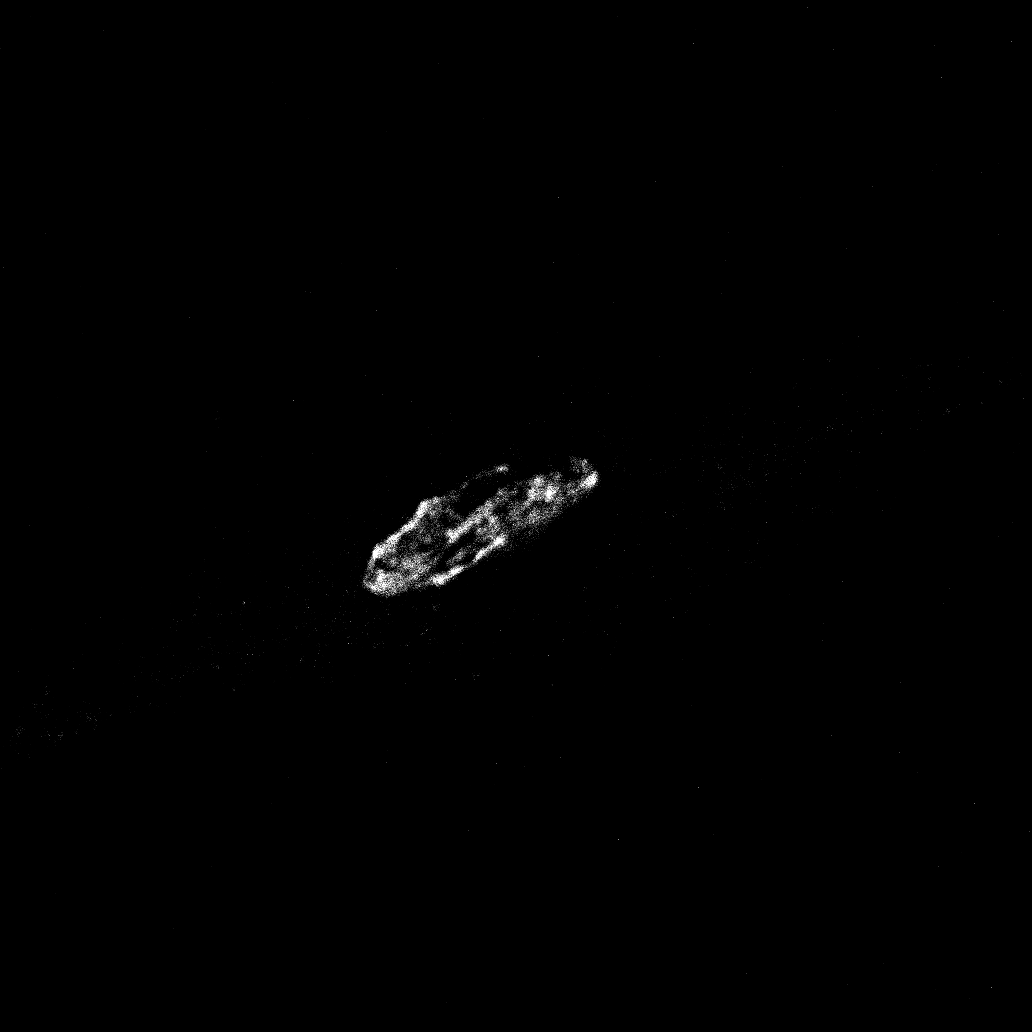

Supplement: Supplementary file 7 — Source data Fig. 5 [file 44319_2025_423_MOESM7_ESM.zip › Figure 5/5A/ARF1 R19C_8h cGAMP_GM130.tif]

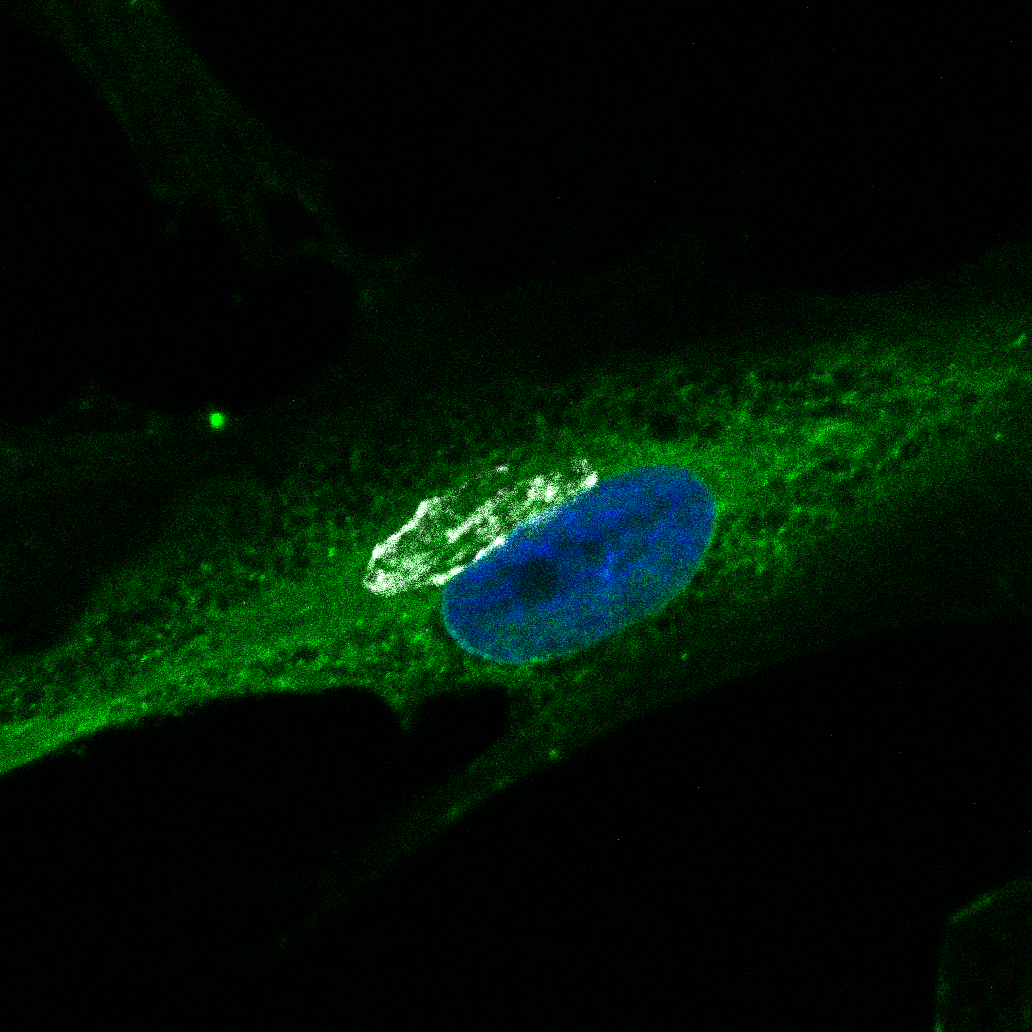

Supplement: Supplementary file 7 — Source data Fig. 5 [file 44319_2025_423_MOESM7_ESM.zip › Figure 5/5A/ARF1 R19C_8h cGAMP_merge.tif]

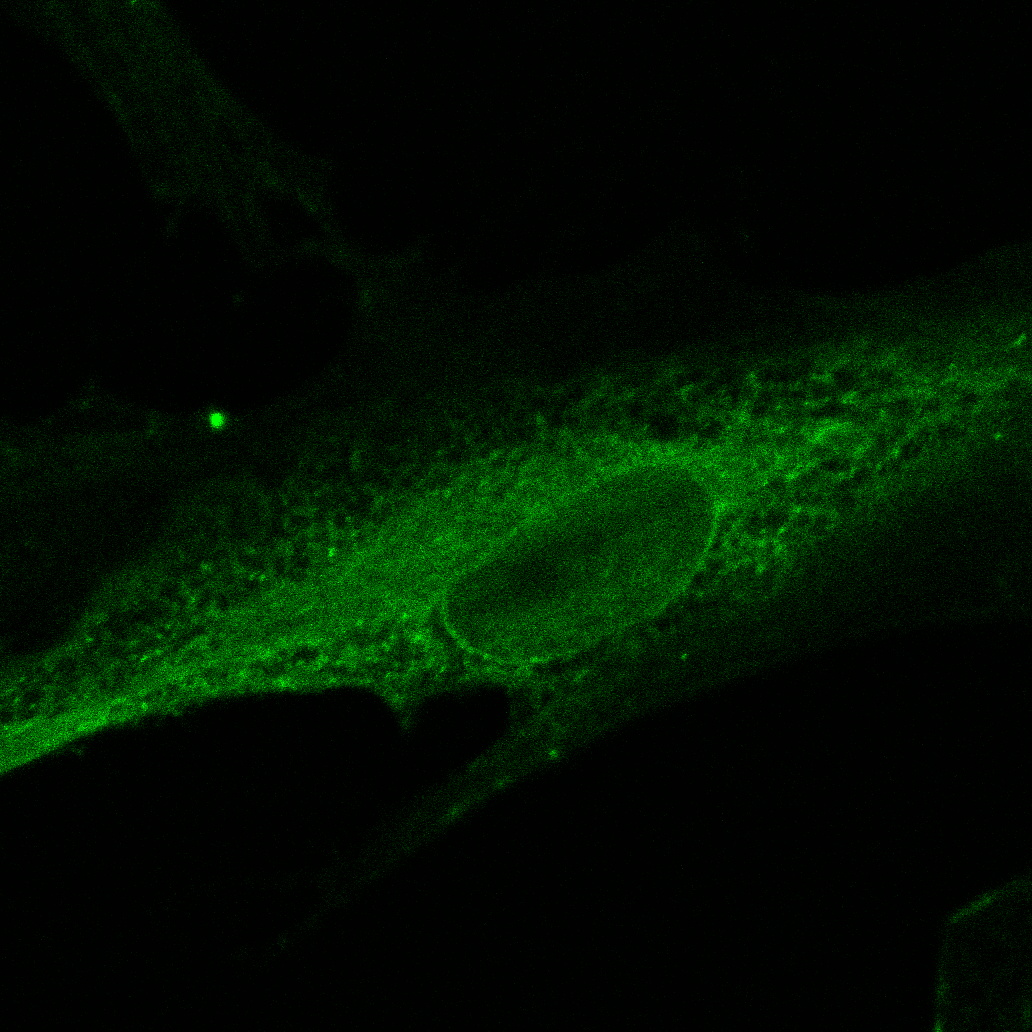

Supplement: Supplementary file 7 — Source data Fig. 5 [file 44319_2025_423_MOESM7_ESM.zip › Figure 5/5A/ARF1 R19C_8h cGAMP_STING.tif]

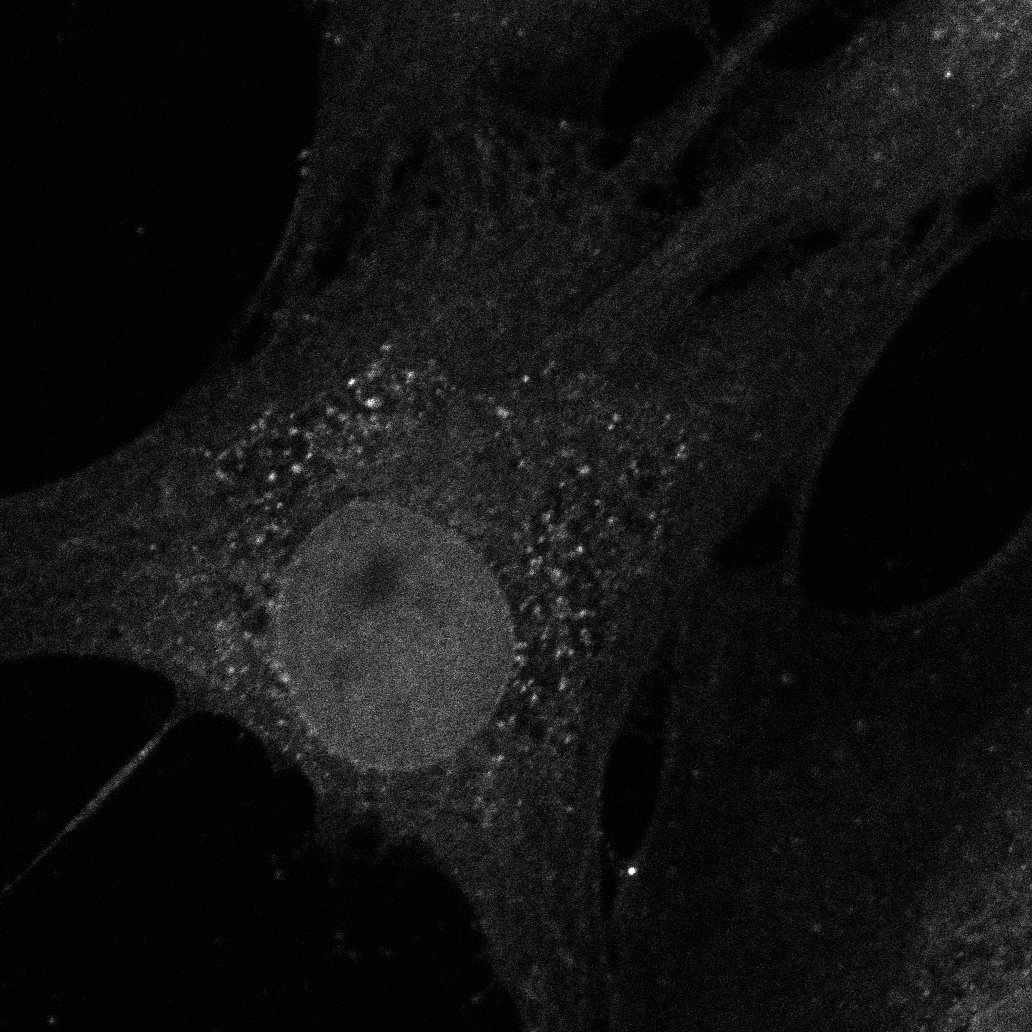

Supplement: Supplementary file 7 — Source data Fig. 5 [file 44319_2025_423_MOESM7_ESM.zip › Figure 5/5A/ARF1 WT_24h cGAMP.tif]

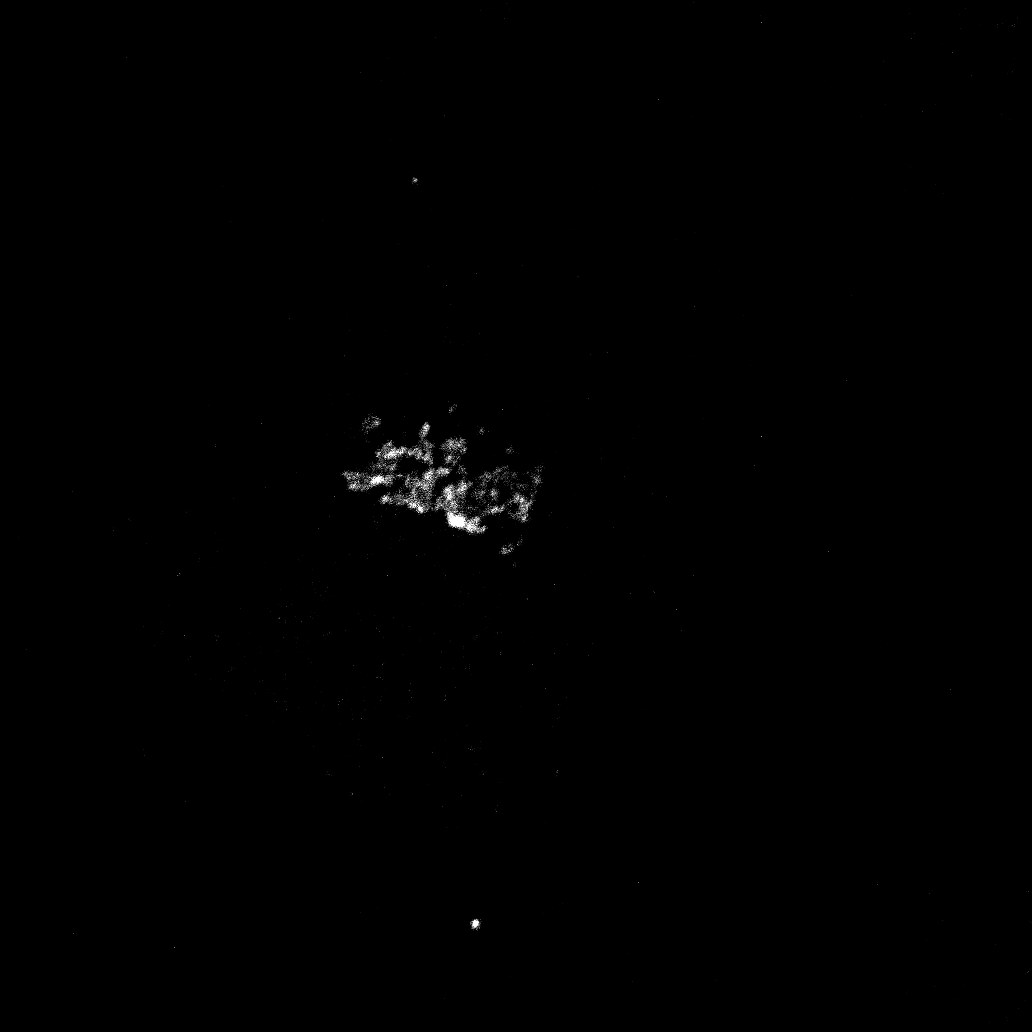

Supplement: Supplementary file 7 — Source data Fig. 5 [file 44319_2025_423_MOESM7_ESM.zip › Figure 5/5A/ARF1 WT_24h cGAMP_GM130.tif]

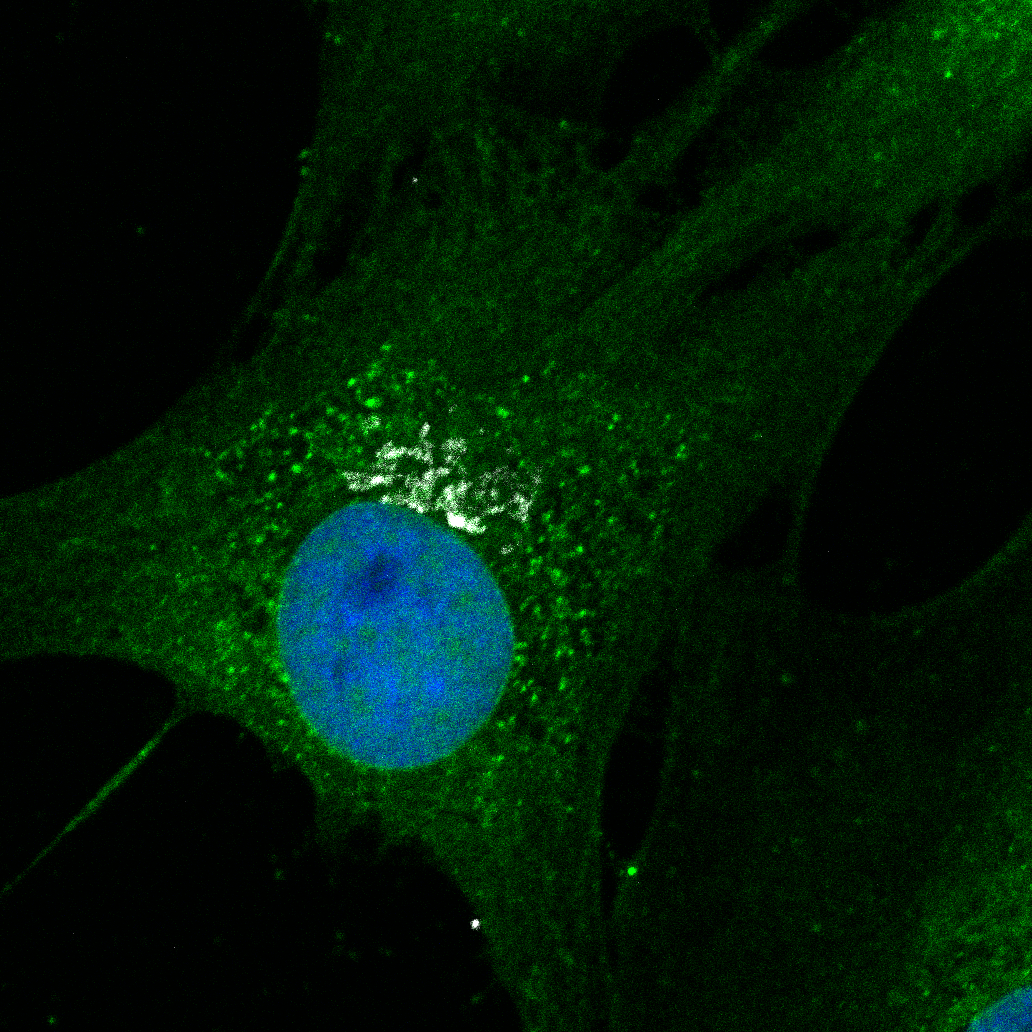

Supplement: Supplementary file 7 — Source data Fig. 5 [file 44319_2025_423_MOESM7_ESM.zip › Figure 5/5A/ARF1 WT_24h cGAMP_merge.tif]

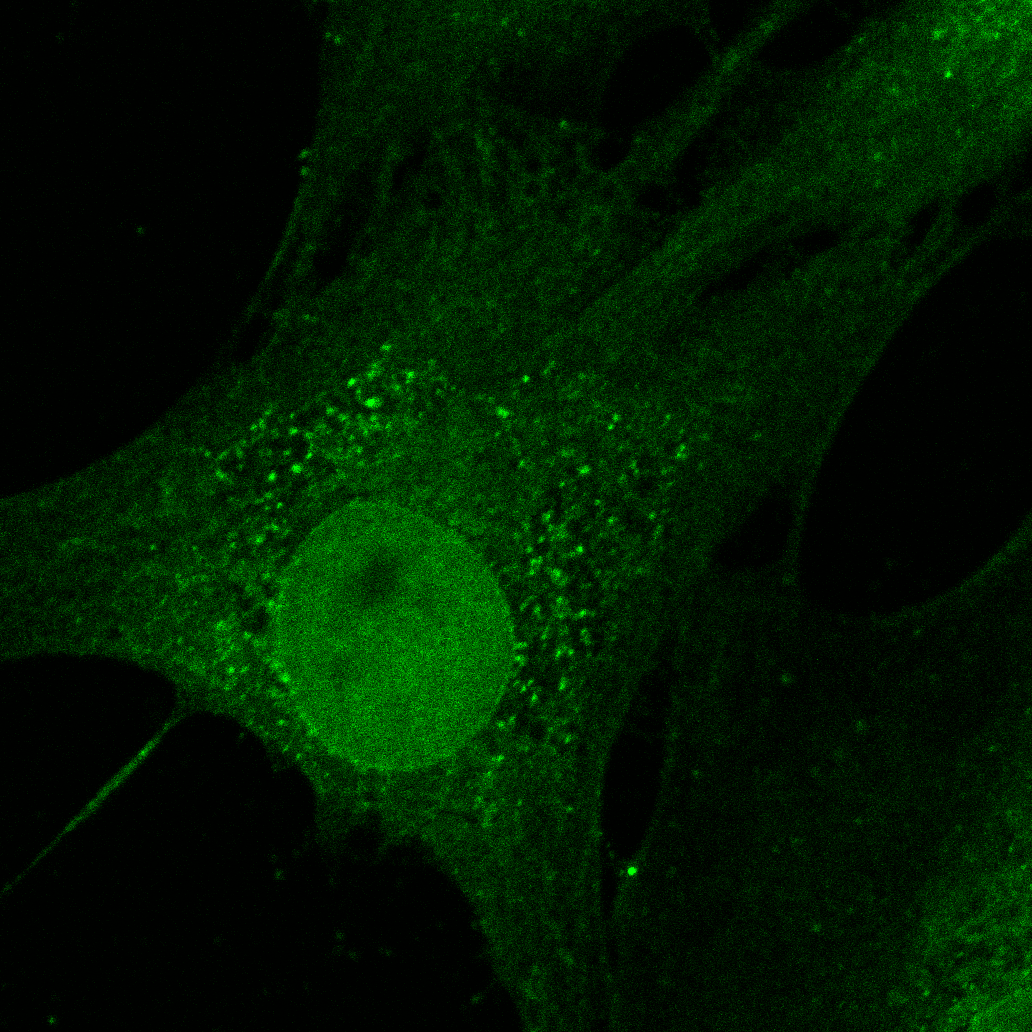

Supplement: Supplementary file 7 — Source data Fig. 5 [file 44319_2025_423_MOESM7_ESM.zip › Figure 5/5A/ARF1 WT_24h cGAMP_STING.tif]

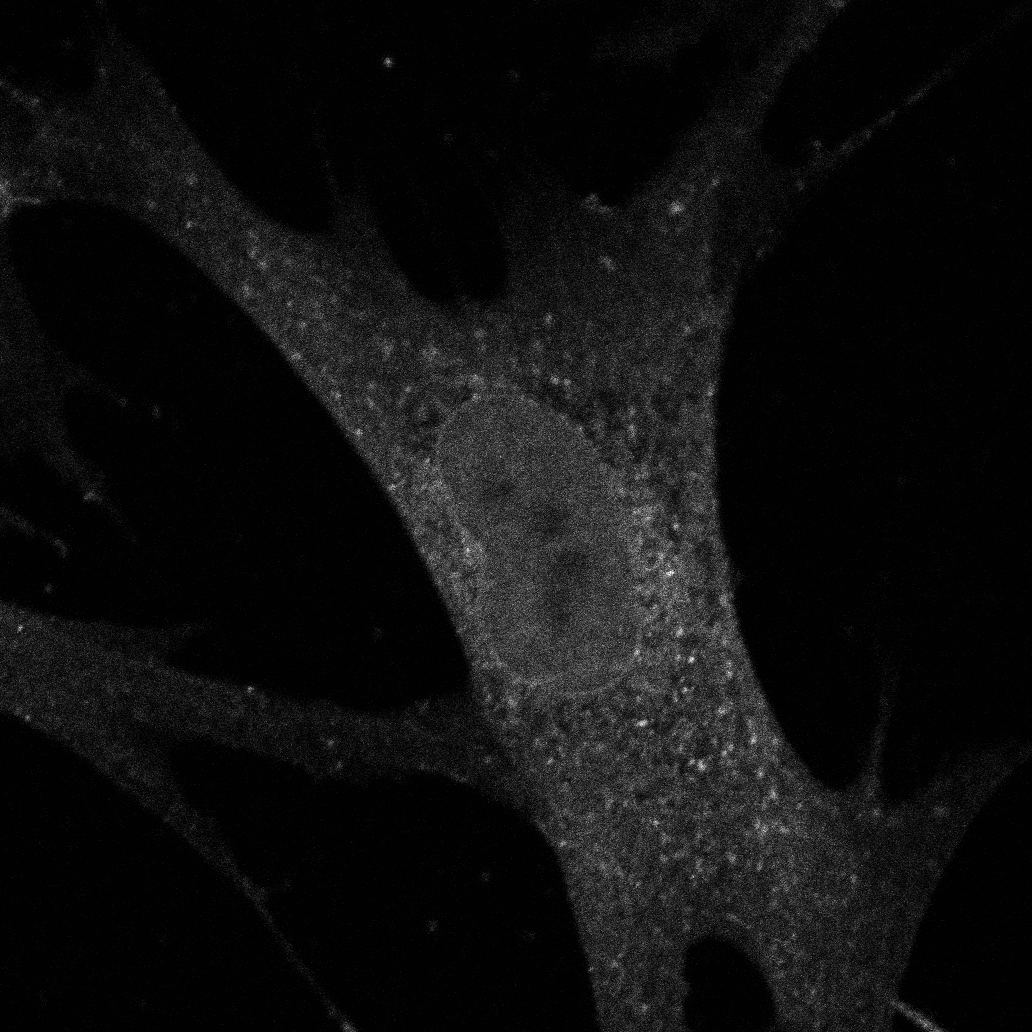

Supplement: Supplementary file 7 — Source data Fig. 5 [file 44319_2025_423_MOESM7_ESM.zip › Figure 5/5A/ARF1 WT_4h cGAMP.tif]

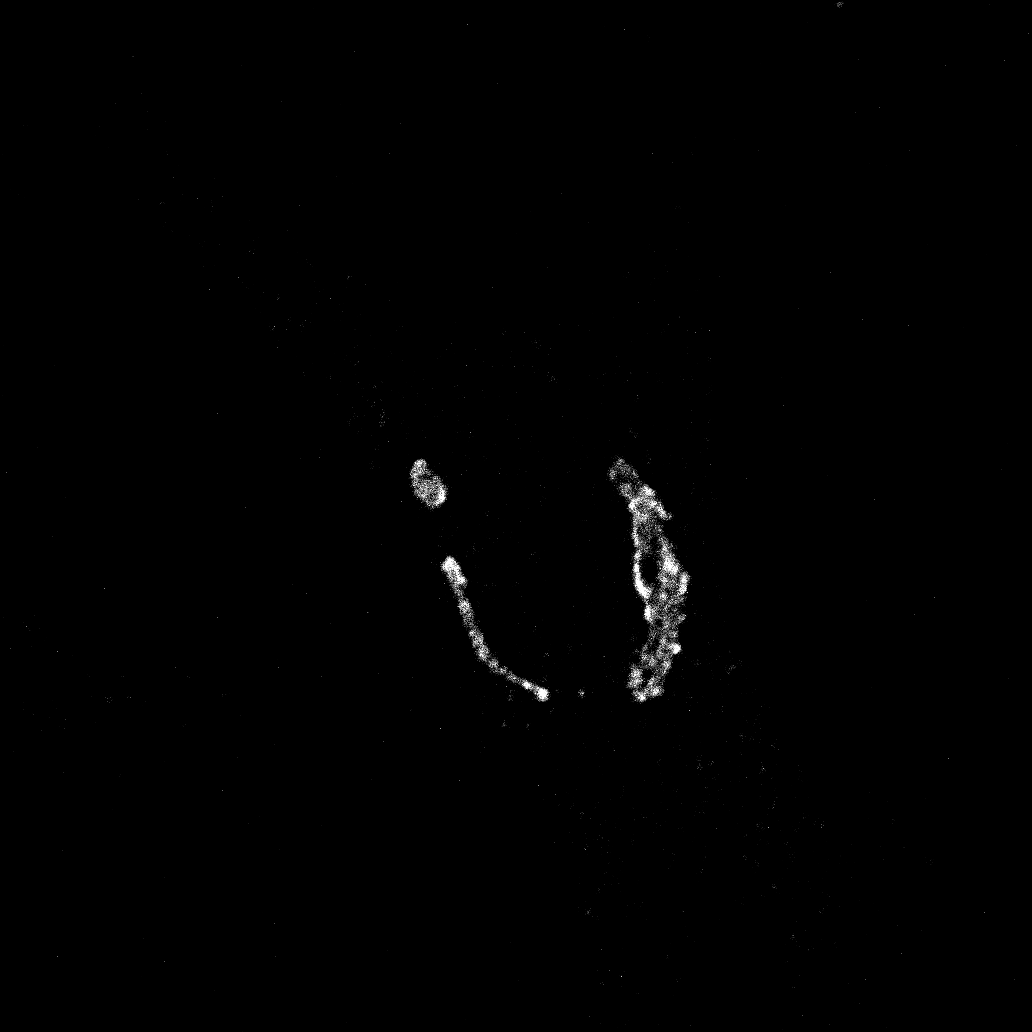

Supplement: Supplementary file 7 — Source data Fig. 5 [file 44319_2025_423_MOESM7_ESM.zip › Figure 5/5A/ARF1 WT_4h cGAMP_GM130.tif]

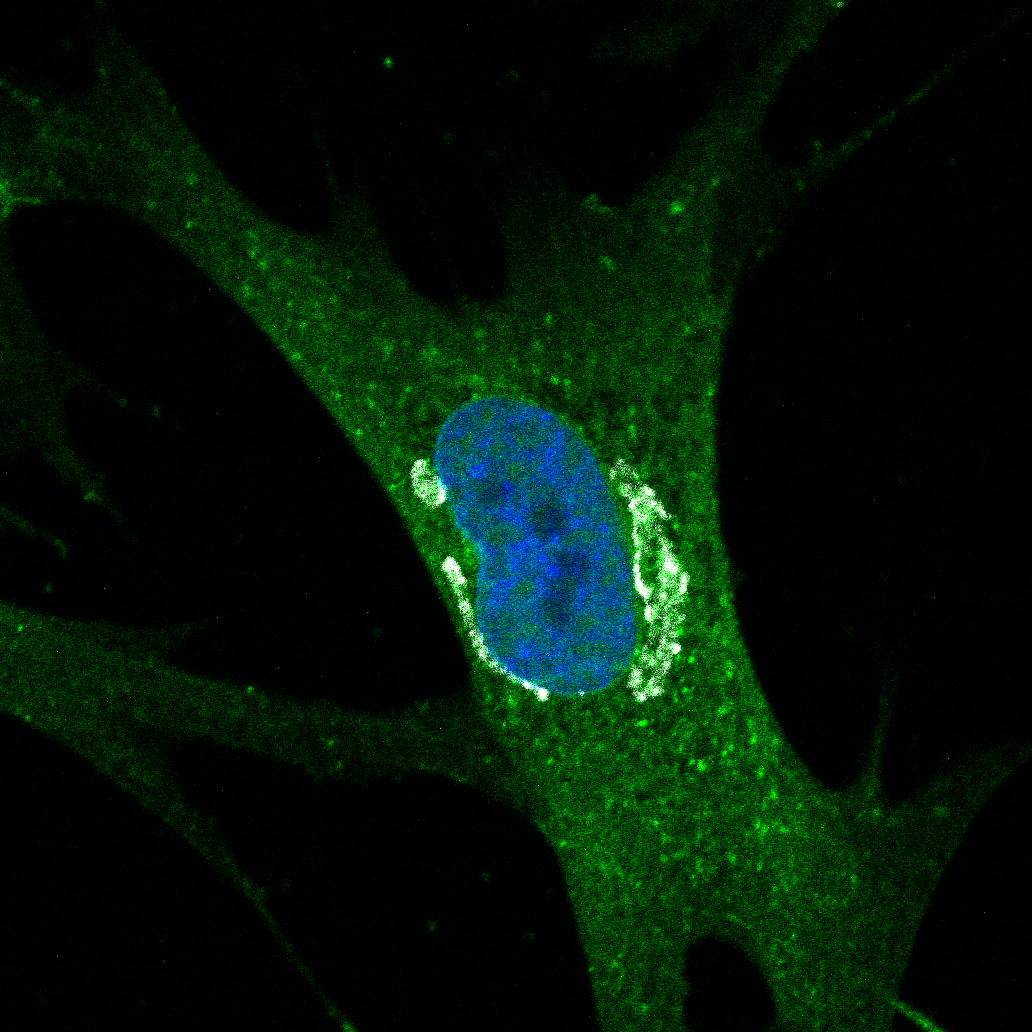

Supplement: Supplementary file 7 — Source data Fig. 5 [file 44319_2025_423_MOESM7_ESM.zip › Figure 5/5A/ARF1 WT_4h cGAMP_merge.tif]

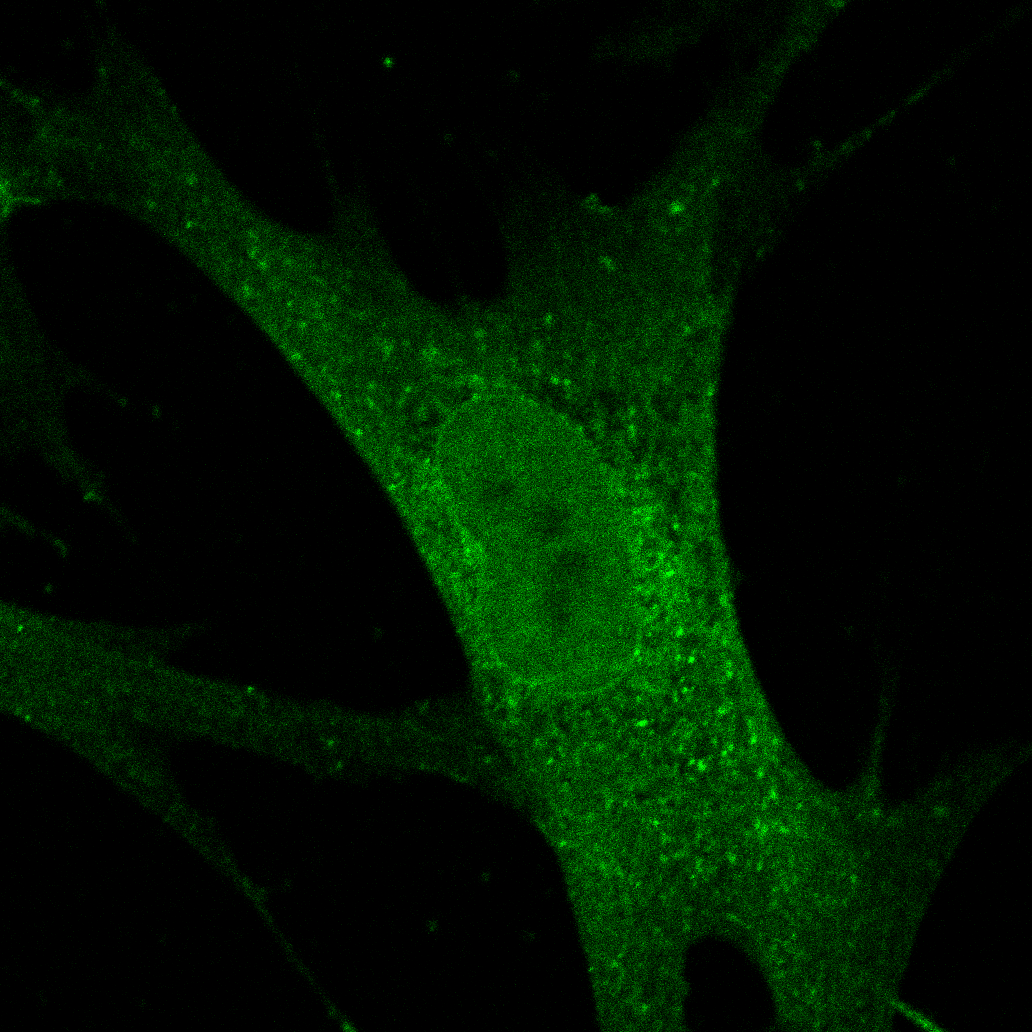

Supplement: Supplementary file 7 — Source data Fig. 5 [file 44319_2025_423_MOESM7_ESM.zip › Figure 5/5A/ARF1 WT_4h cGAMP_STING.tif]

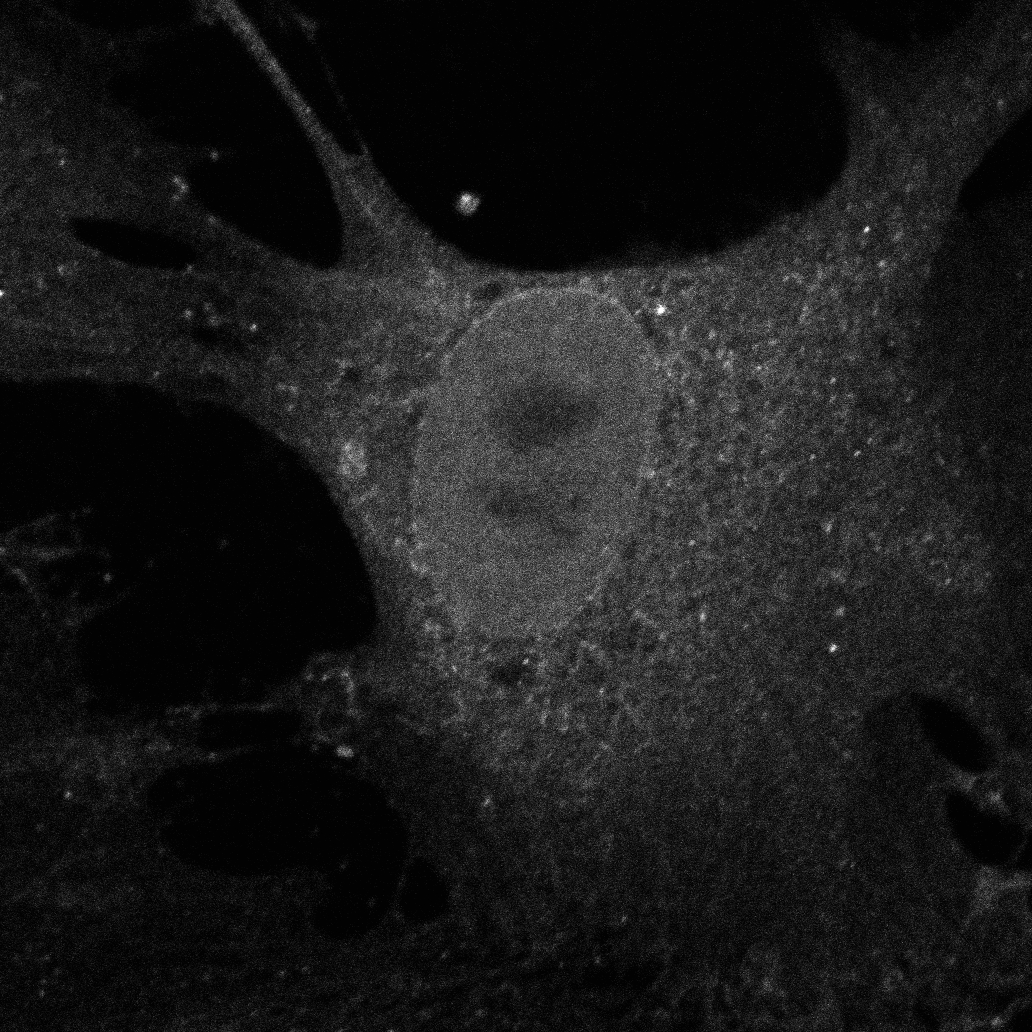

Supplement: Supplementary file 7 — Source data Fig. 5 [file 44319_2025_423_MOESM7_ESM.zip › Figure 5/5A/ARF1 WT_8h cGAMP.tif]

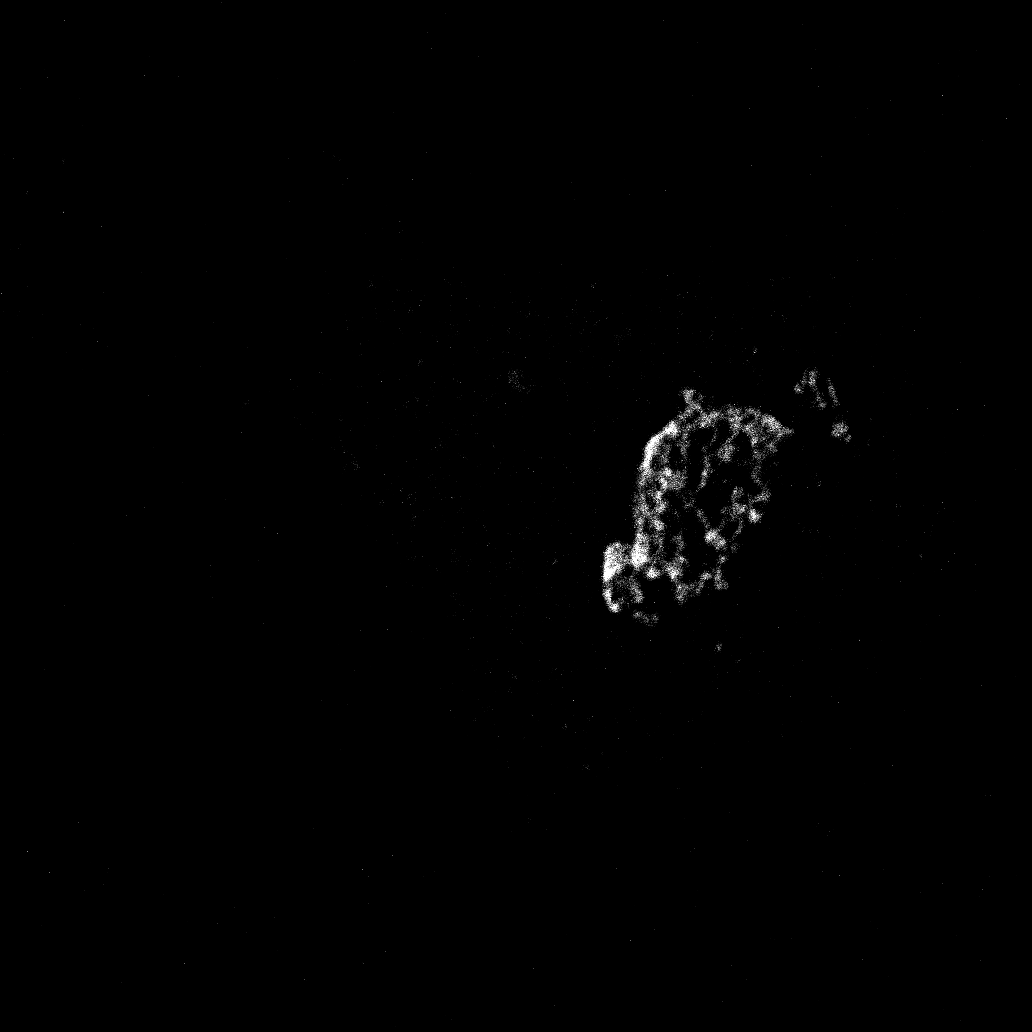

Supplement: Supplementary file 7 — Source data Fig. 5 [file 44319_2025_423_MOESM7_ESM.zip › Figure 5/5A/ARF1 WT_8h cGAMP_GM130.tif]

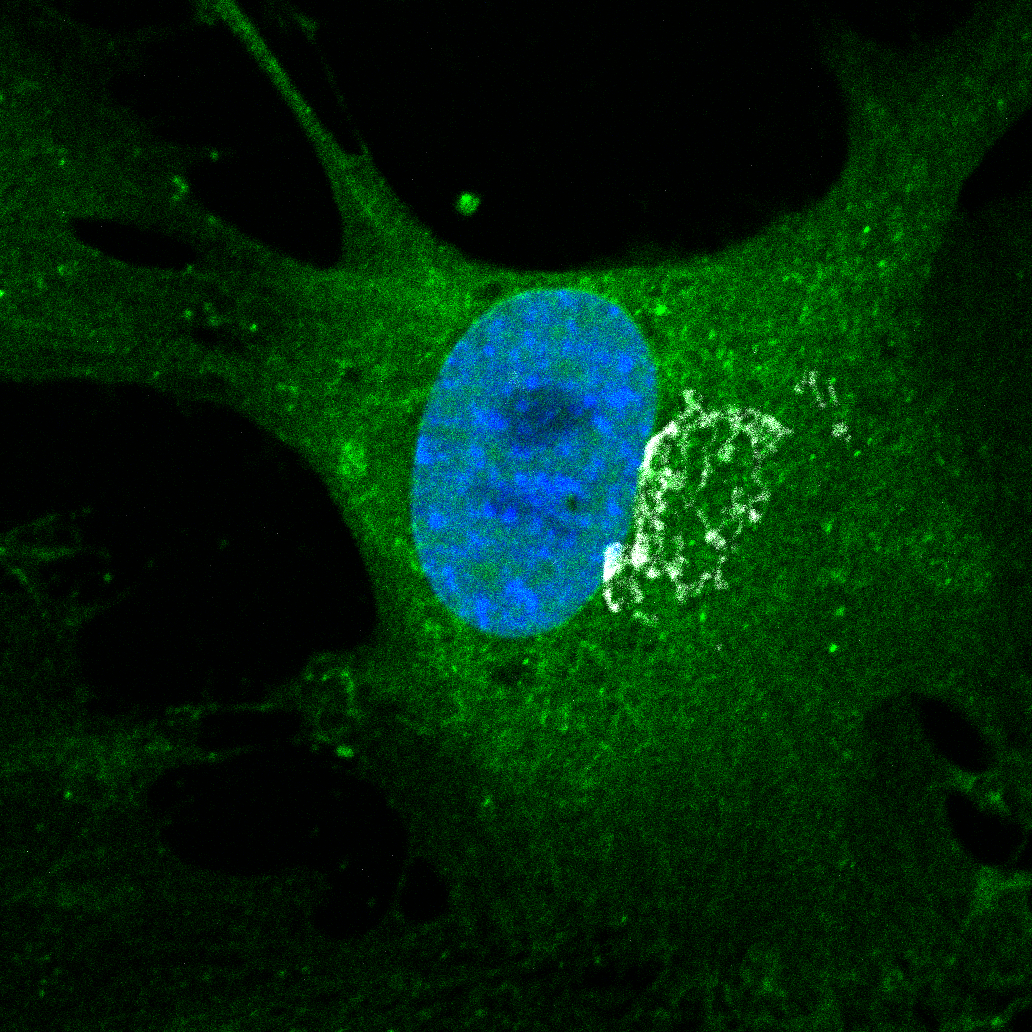

Supplement: Supplementary file 7 — Source data Fig. 5 [file 44319_2025_423_MOESM7_ESM.zip › Figure 5/5A/ARF1 WT_8h cGAMP_merge.tif]

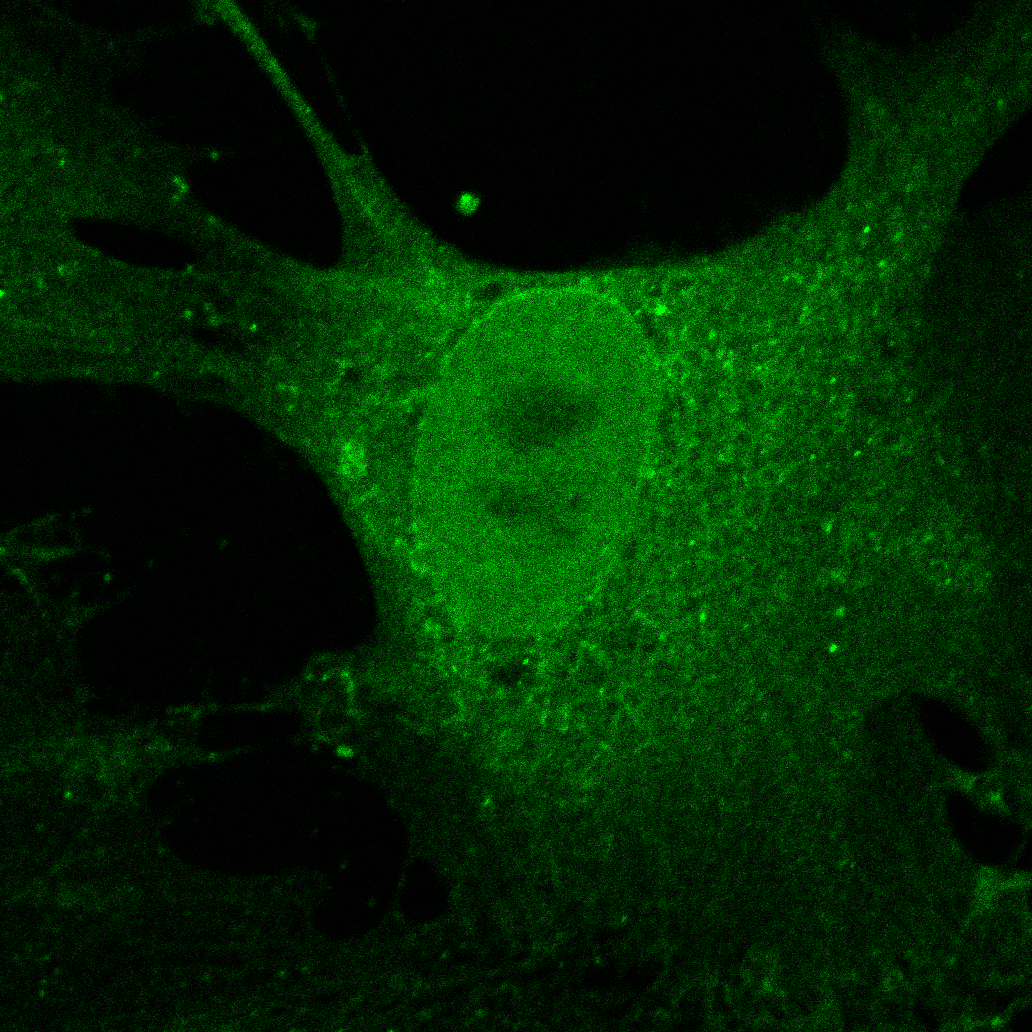

Supplement: Supplementary file 7 — Source data Fig. 5 [file 44319_2025_423_MOESM7_ESM.zip › Figure 5/5A/ARF1 WT_8h cGAMP_STING.tif]

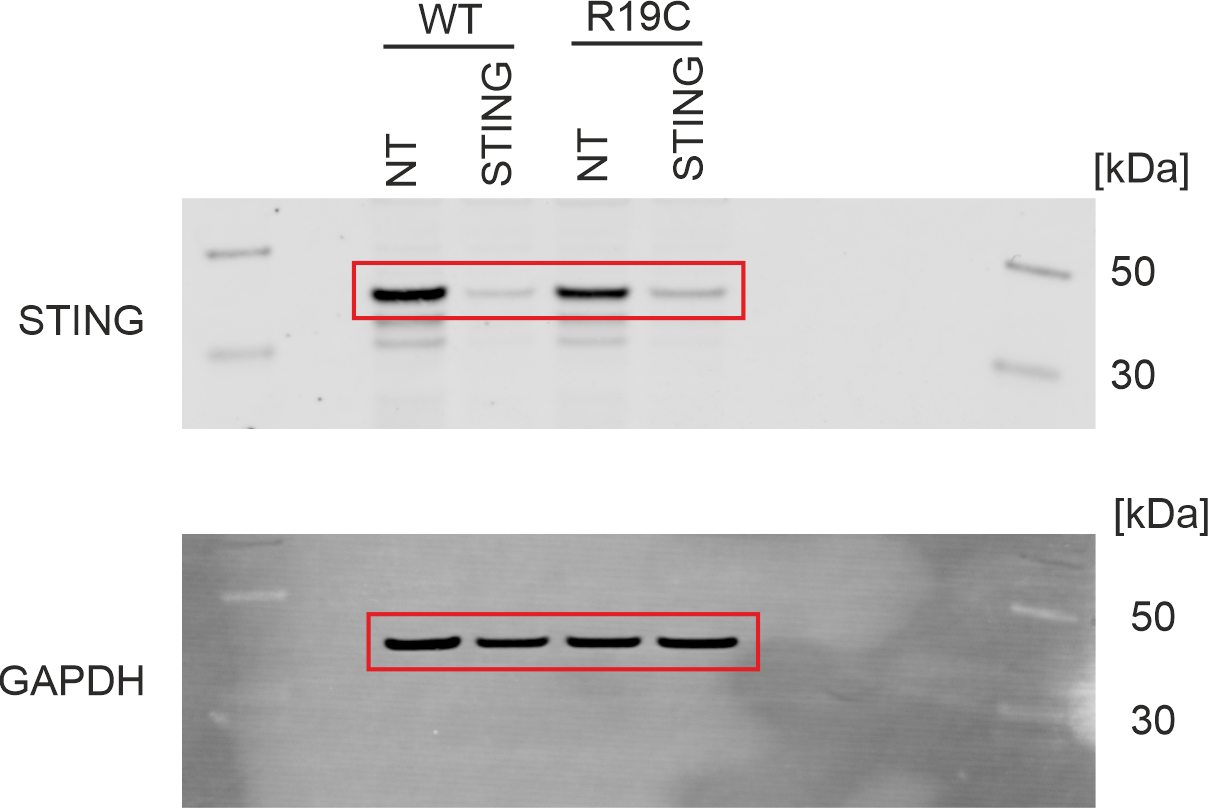

Supplement: Supplementary file 7 — Source data Fig. 5 [file 44319_2025_423_MOESM7_ESM.zip › Figure 5/5F/Western Blot STING, GAPDH - WT, R19C.png]

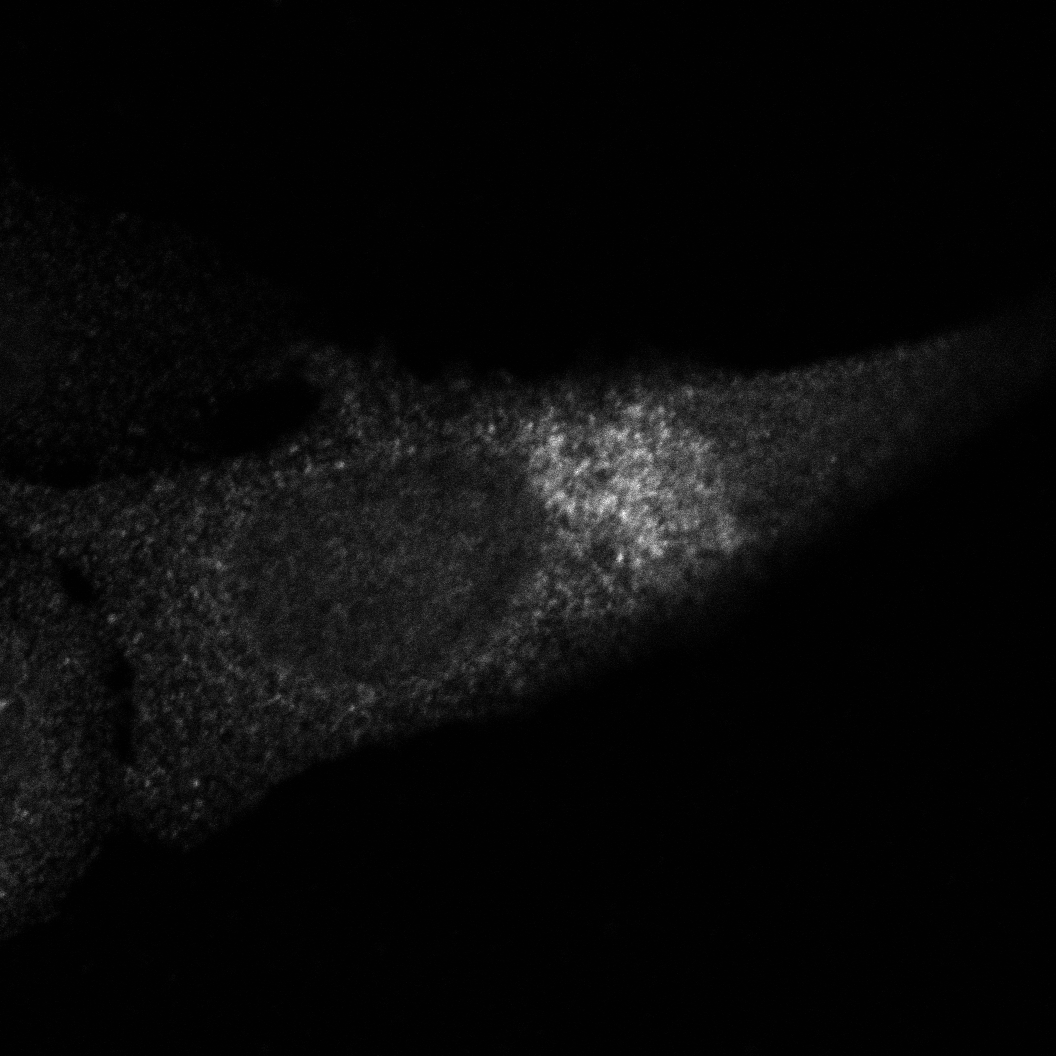

Supplement: Supplementary file 8 — Figure EV2 Source Data [file 44319_2025_423_MOESM8_ESM.zip › Figure EV2/2A/R19C.tif]

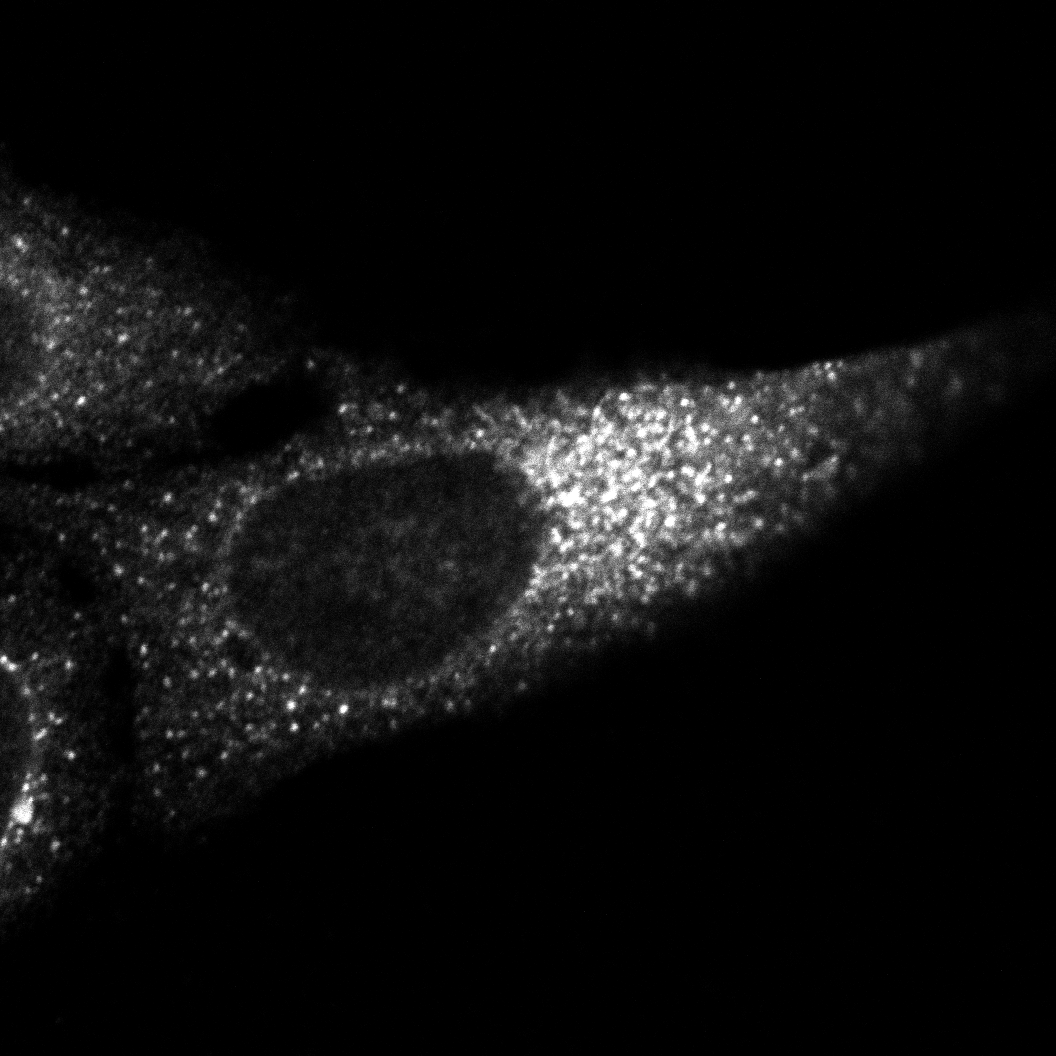

Supplement: Supplementary file 8 — Figure EV2 Source Data [file 44319_2025_423_MOESM8_ESM.zip › Figure EV2/2A/R19C_ERGIC-53.tif]

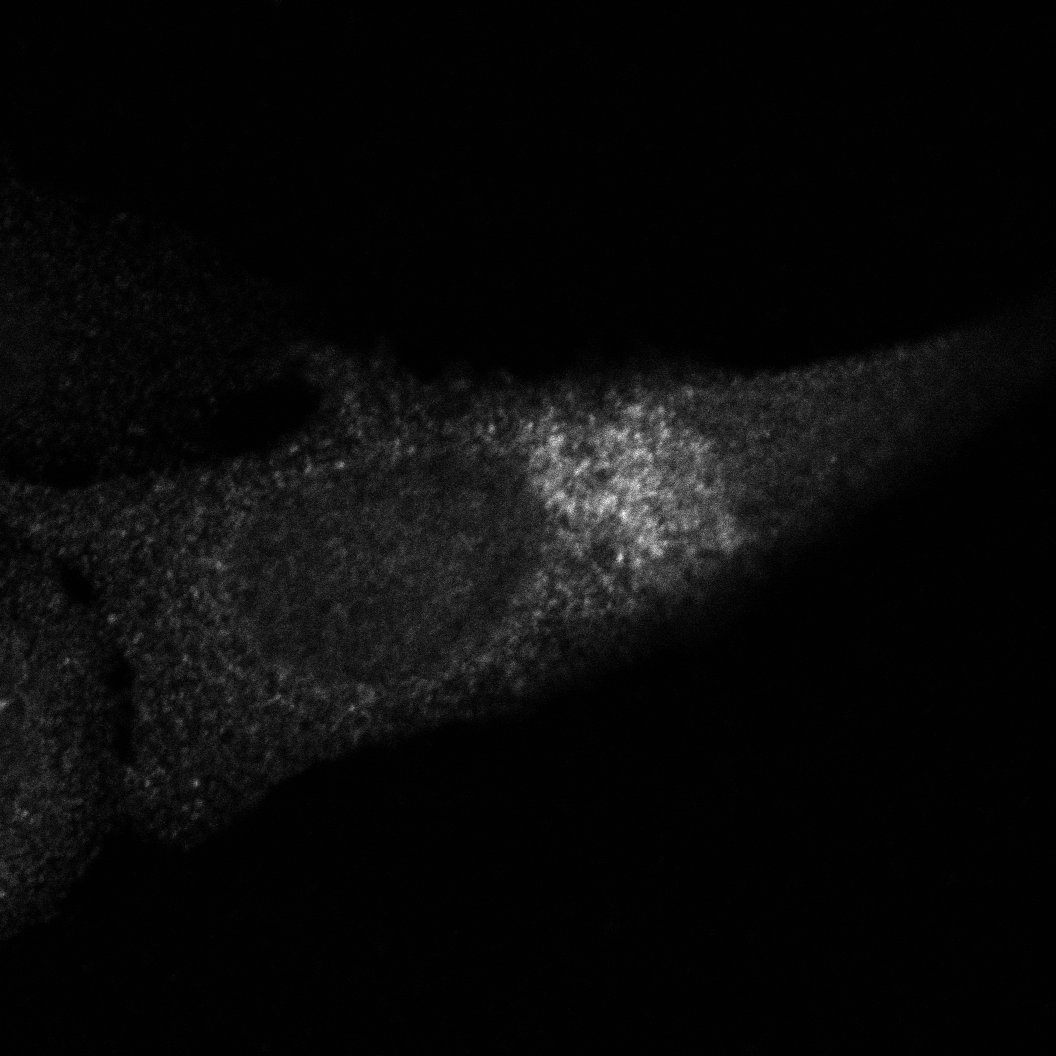

Supplement: Supplementary file 8 — Figure EV2 Source Data [file 44319_2025_423_MOESM8_ESM.zip › Figure EV2/2A/R19C_FLAG.tif]

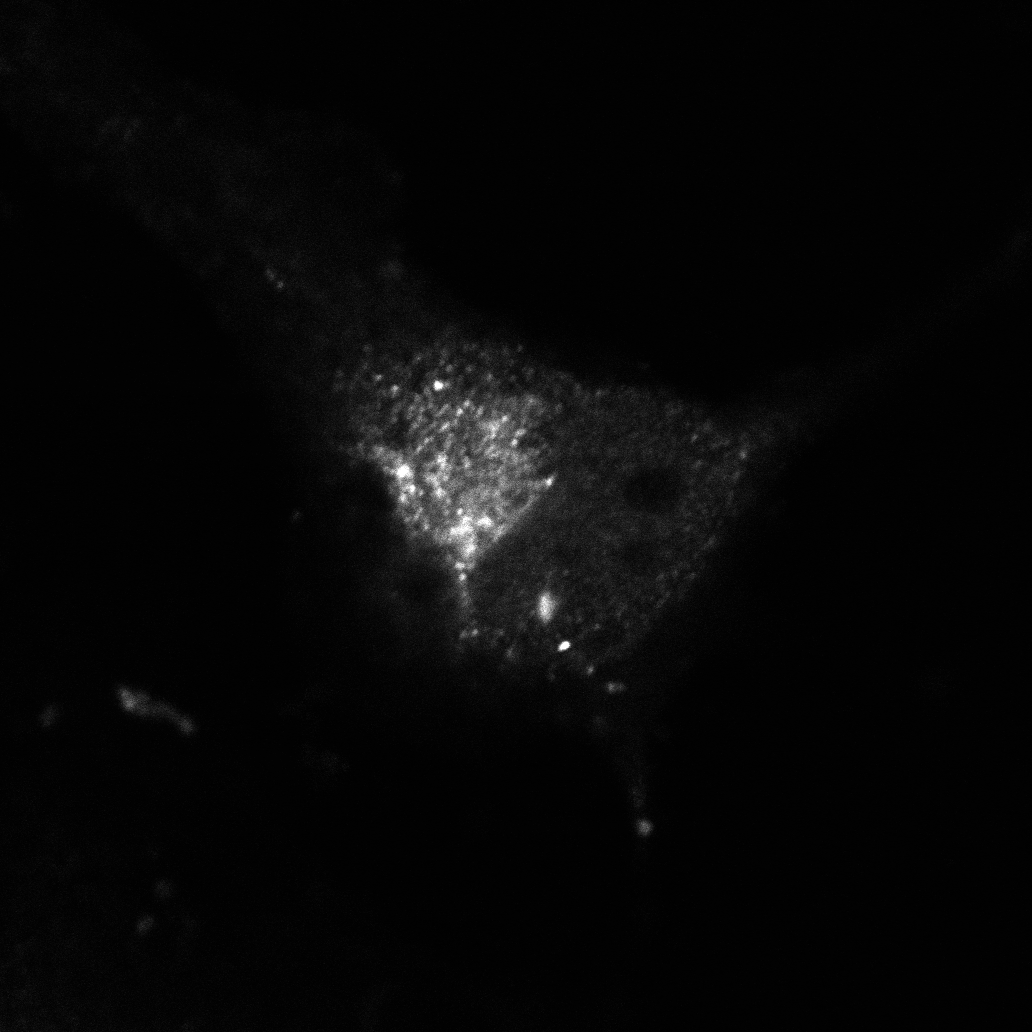

Supplement: Supplementary file 8 — Figure EV2 Source Data [file 44319_2025_423_MOESM8_ESM.zip › Figure EV2/2A/R99C.tif]

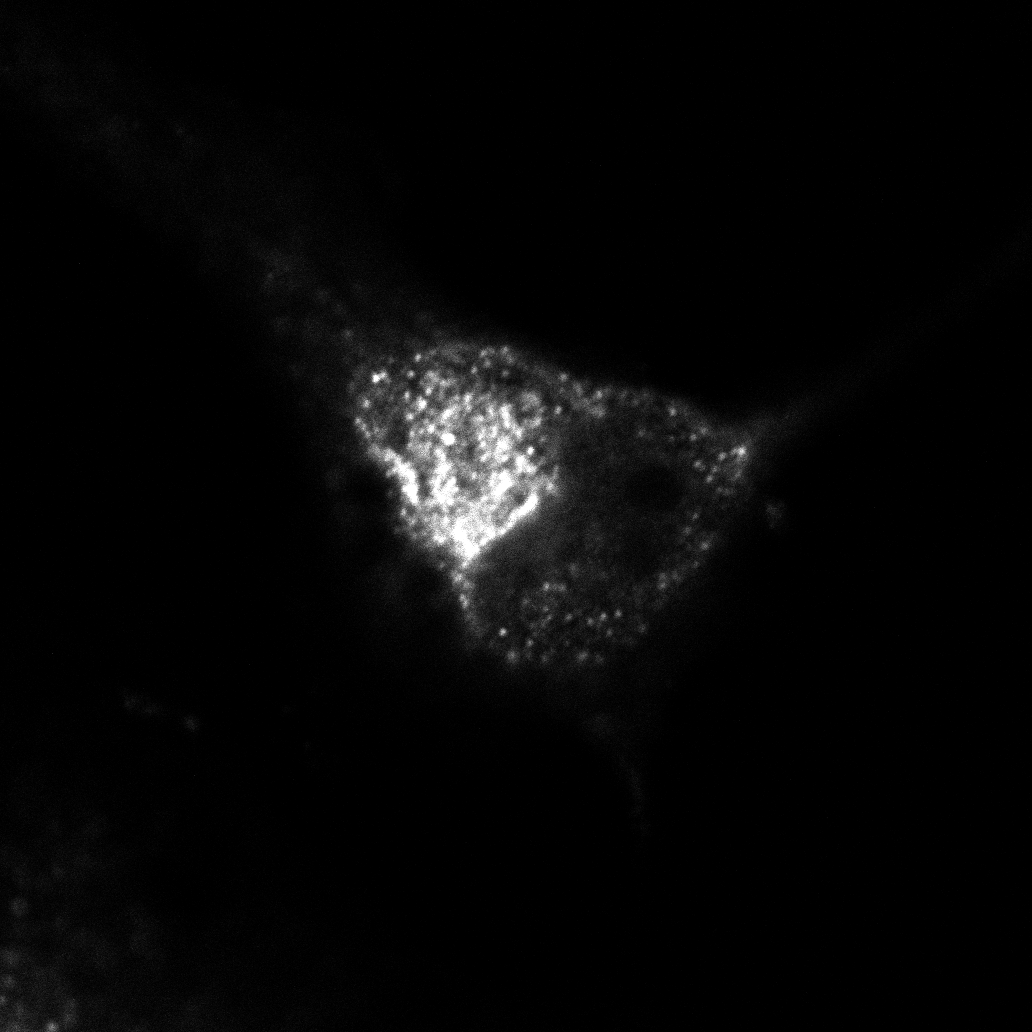

Supplement: Supplementary file 8 — Figure EV2 Source Data [file 44319_2025_423_MOESM8_ESM.zip › Figure EV2/2A/R99C_ERGIC-53.tif]

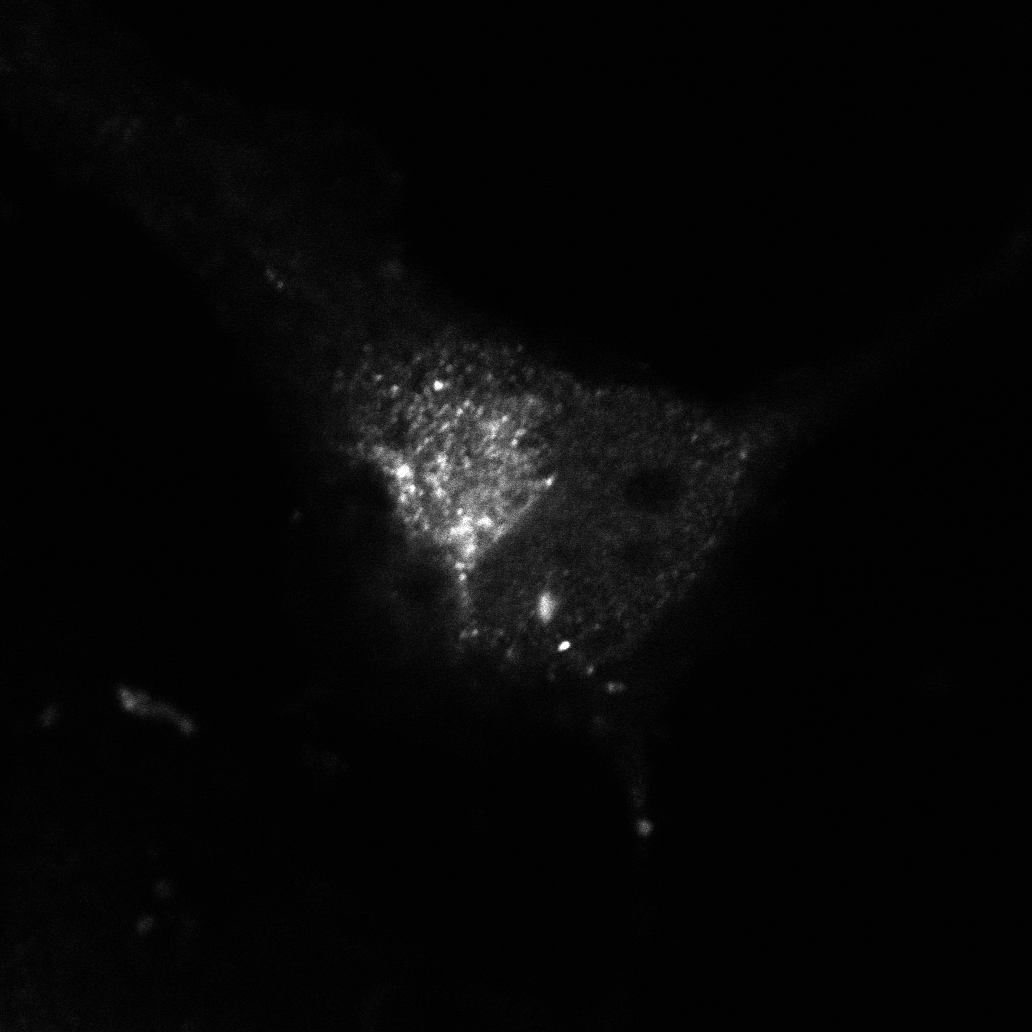

Supplement: Supplementary file 8 — Figure EV2 Source Data [file 44319_2025_423_MOESM8_ESM.zip › Figure EV2/2A/R99C_FLAG.tif]

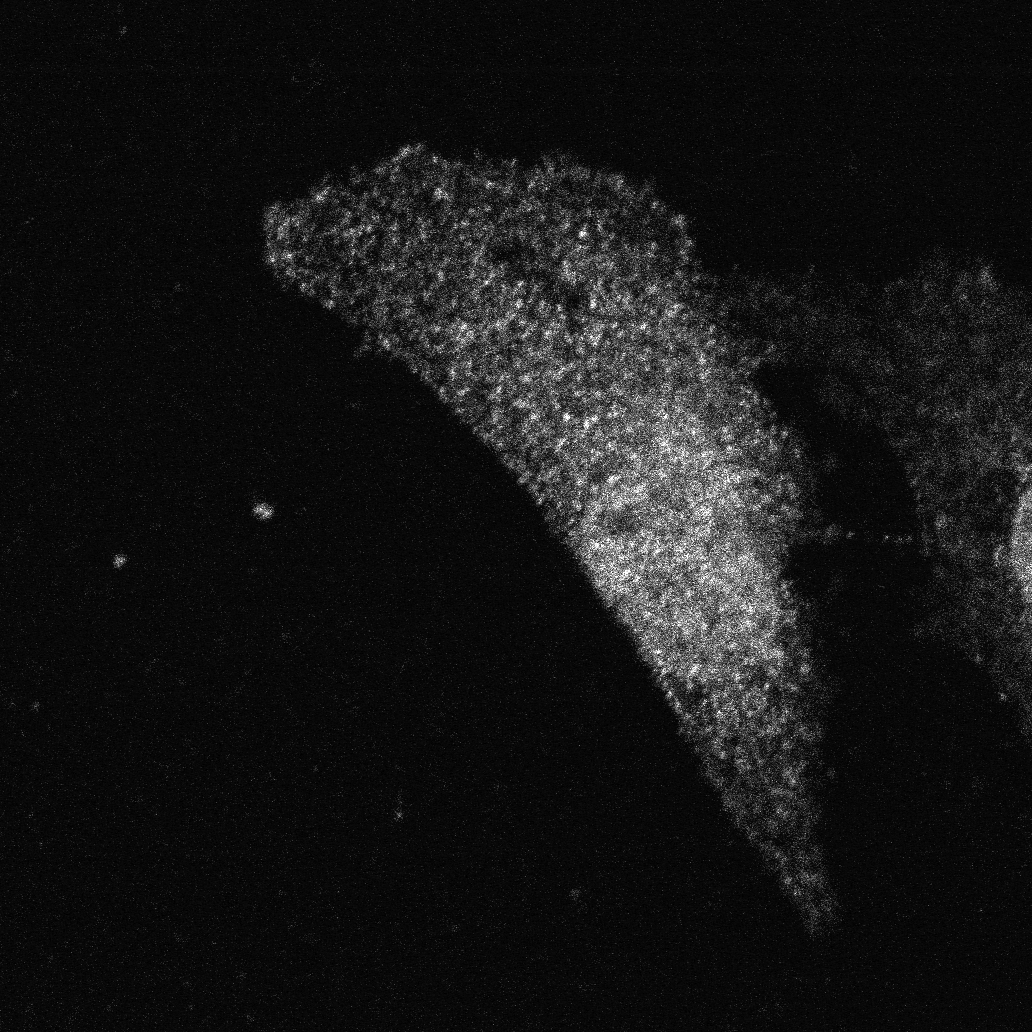

Supplement: Supplementary file 8 — Figure EV2 Source Data [file 44319_2025_423_MOESM8_ESM.zip › Figure EV2/2A/Vector.tif]

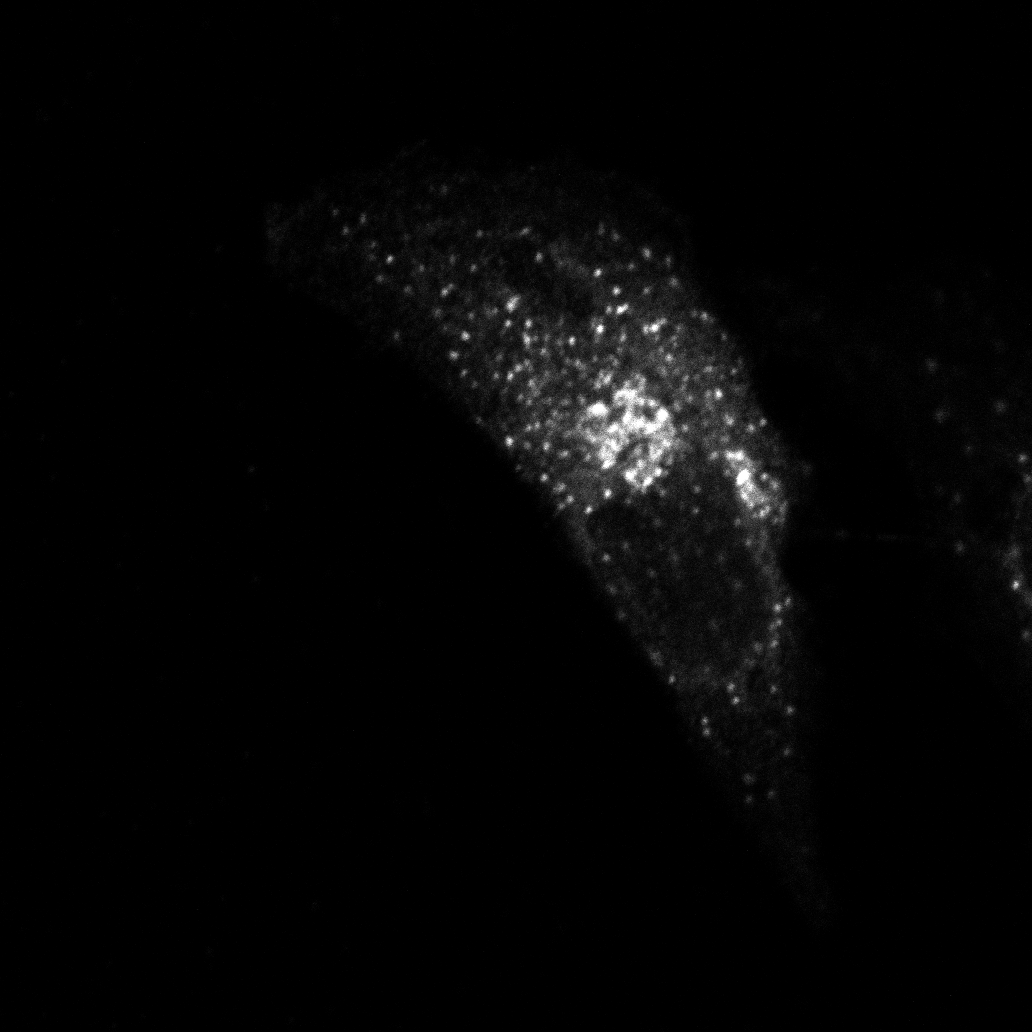

Supplement: Supplementary file 8 — Figure EV2 Source Data [file 44319_2025_423_MOESM8_ESM.zip › Figure EV2/2A/Vector_ERGIC-53.tif]

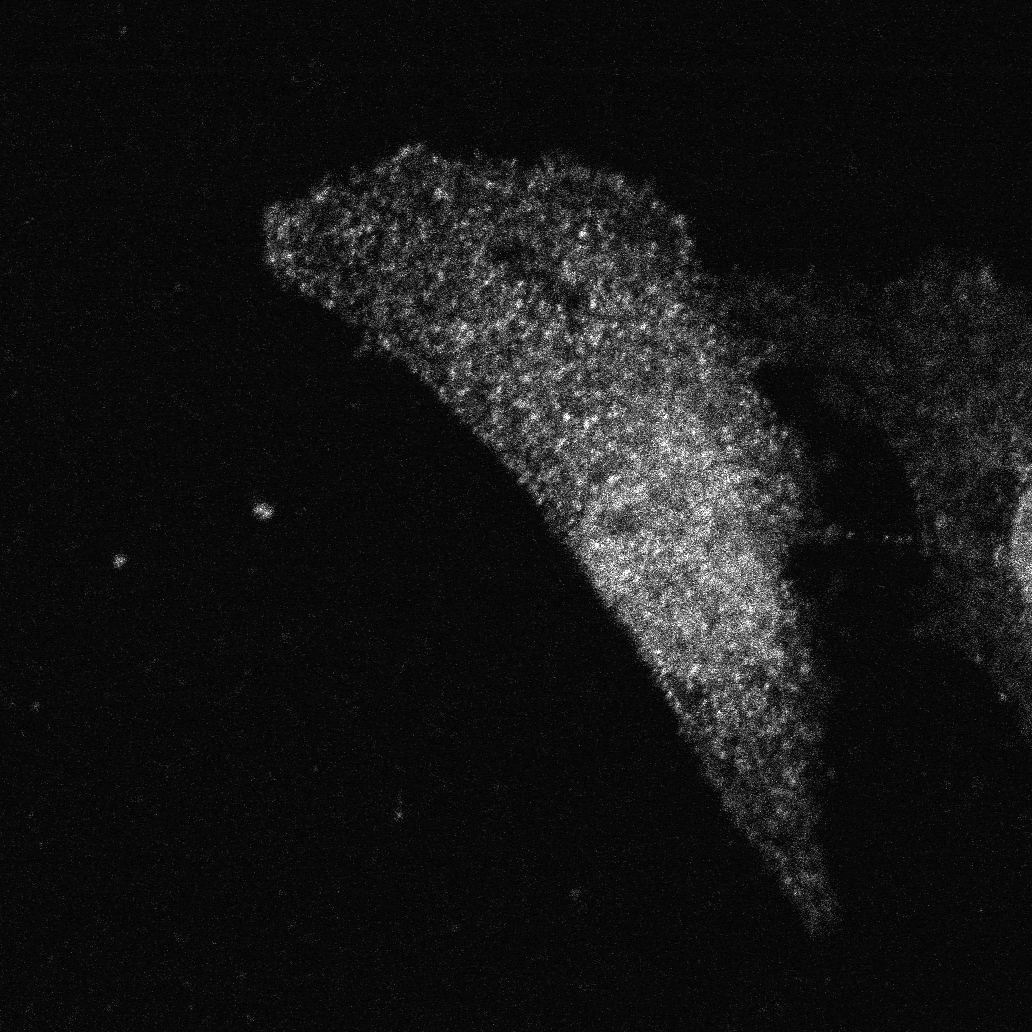

Supplement: Supplementary file 8 — Figure EV2 Source Data [file 44319_2025_423_MOESM8_ESM.zip › Figure EV2/2A/Vector_FLAG.tif]

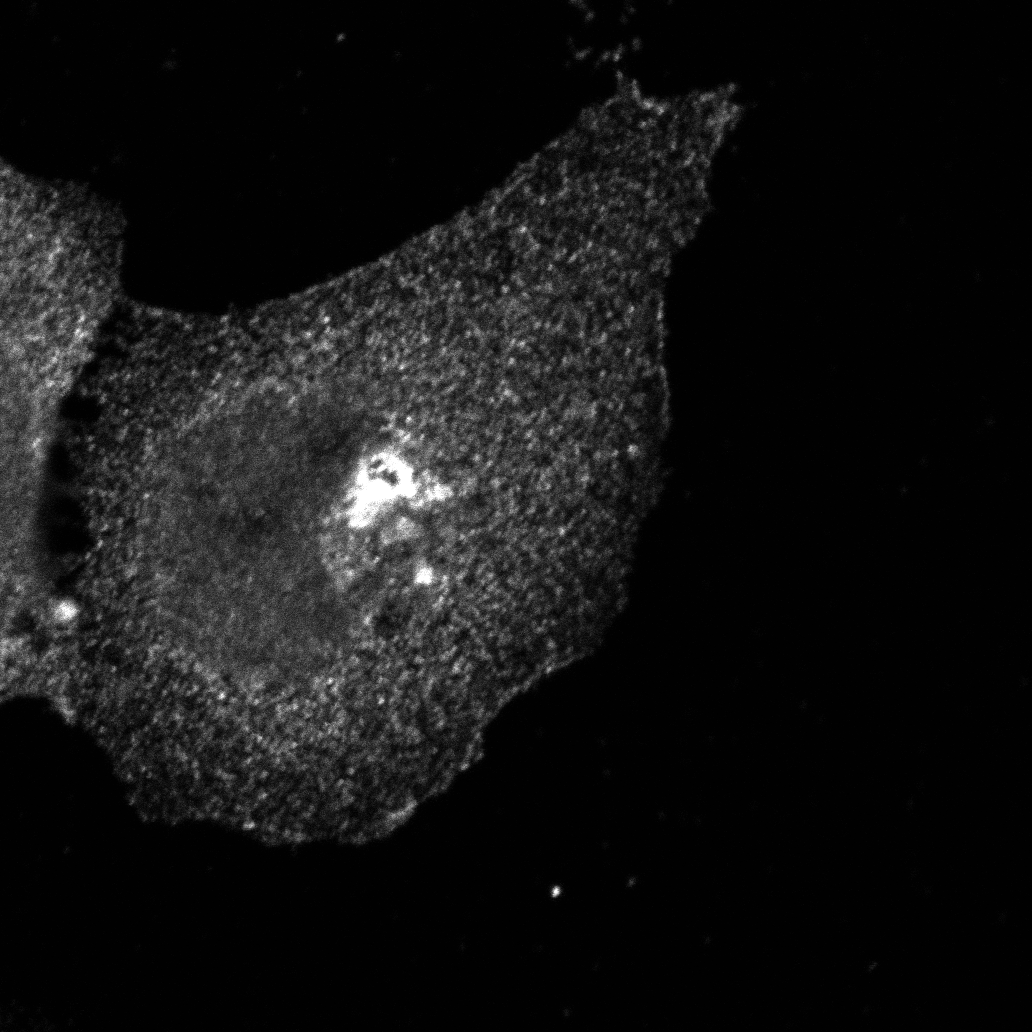

Supplement: Supplementary file 8 — Figure EV2 Source Data [file 44319_2025_423_MOESM8_ESM.zip › Figure EV2/2A/WT.tif]

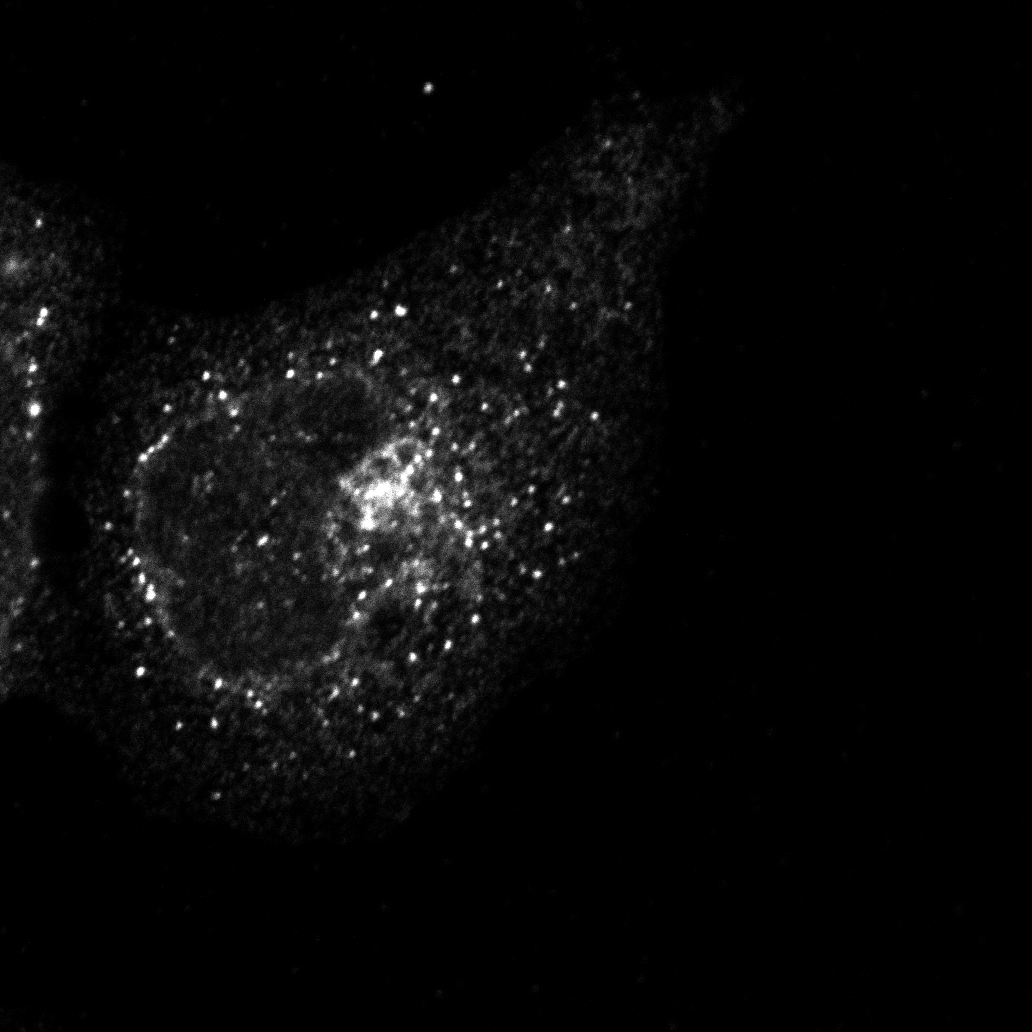

Supplement: Supplementary file 8 — Figure EV2 Source Data [file 44319_2025_423_MOESM8_ESM.zip › Figure EV2/2A/WT_ERGIC-53.tif]

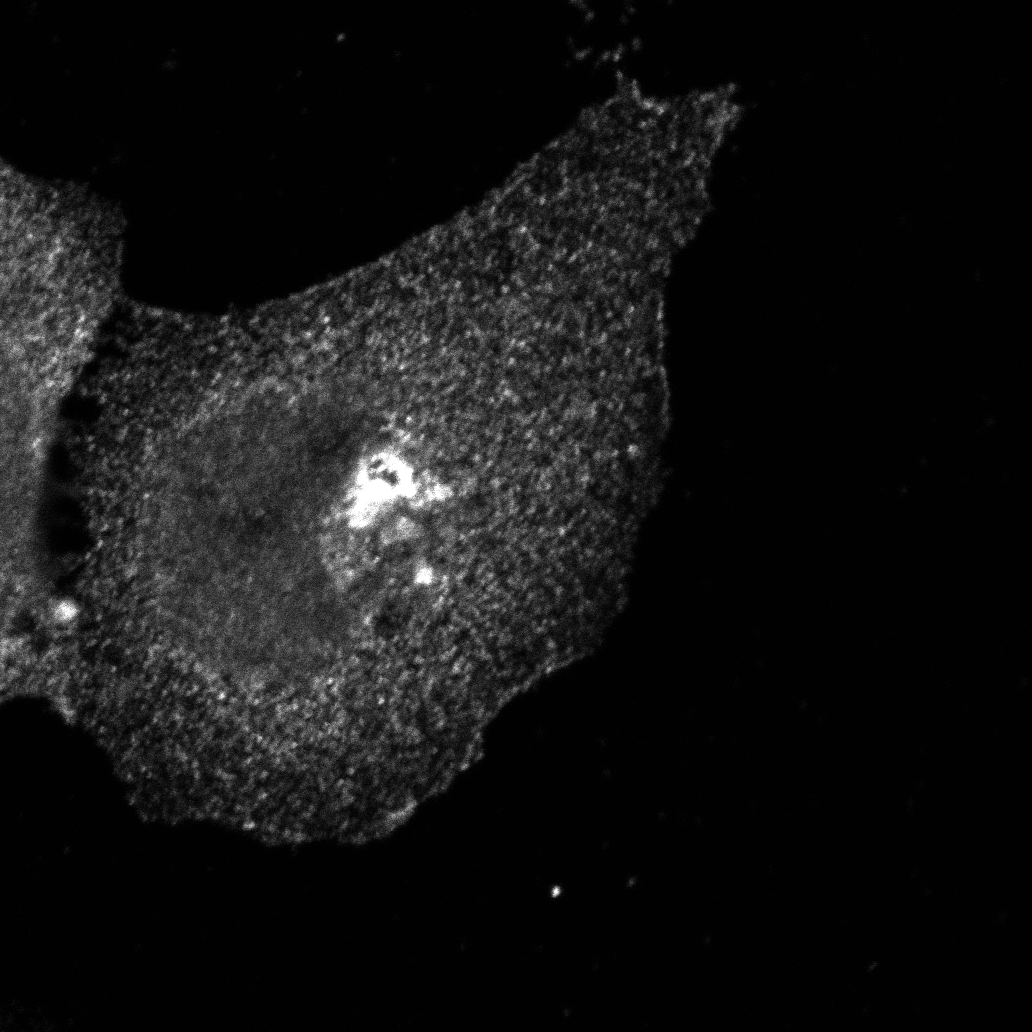

Supplement: Supplementary file 8 — Figure EV2 Source Data [file 44319_2025_423_MOESM8_ESM.zip › Figure EV2/2A/WT_FLAG.tif]

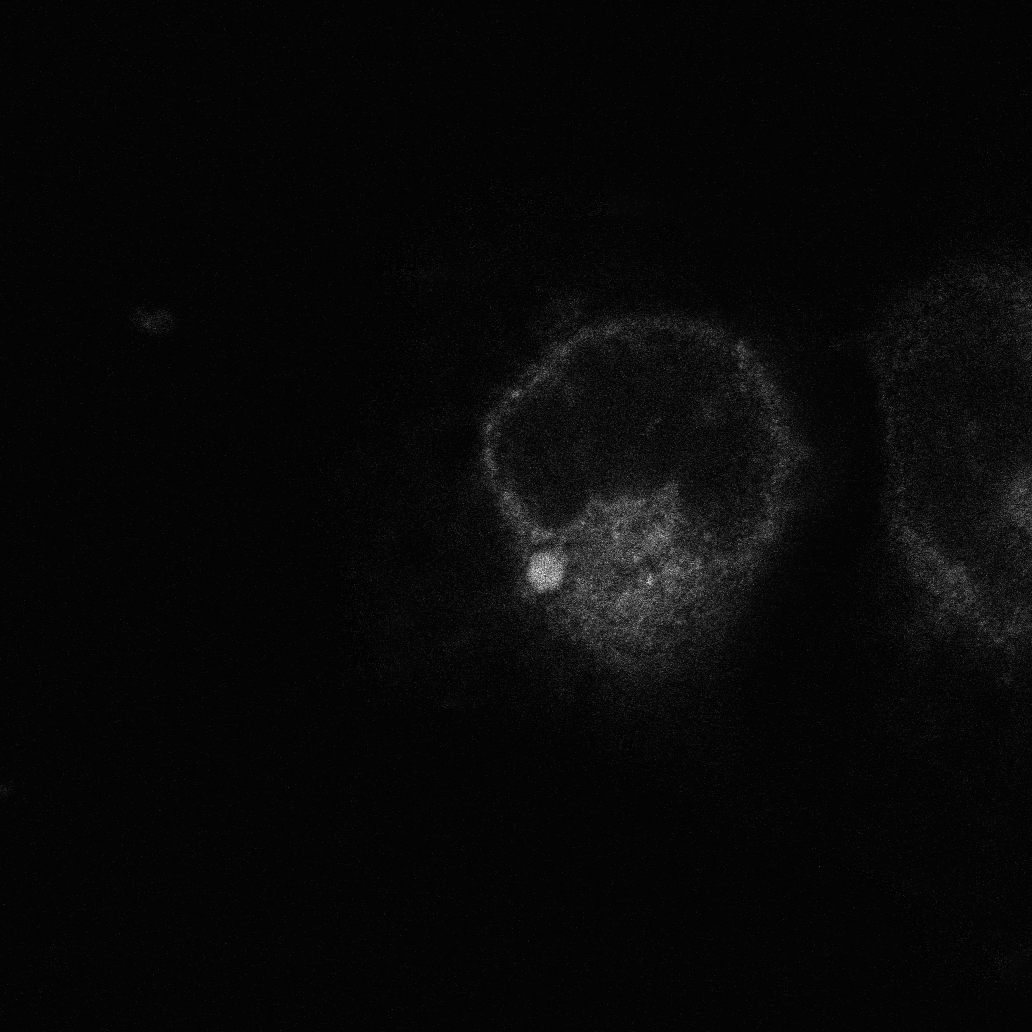

Supplement: Supplementary file 9 — Figure EV3 Source Data [file 44319_2025_423_MOESM9_ESM.zip › Figure EV3/3A/R19C_37-32-40.tif]

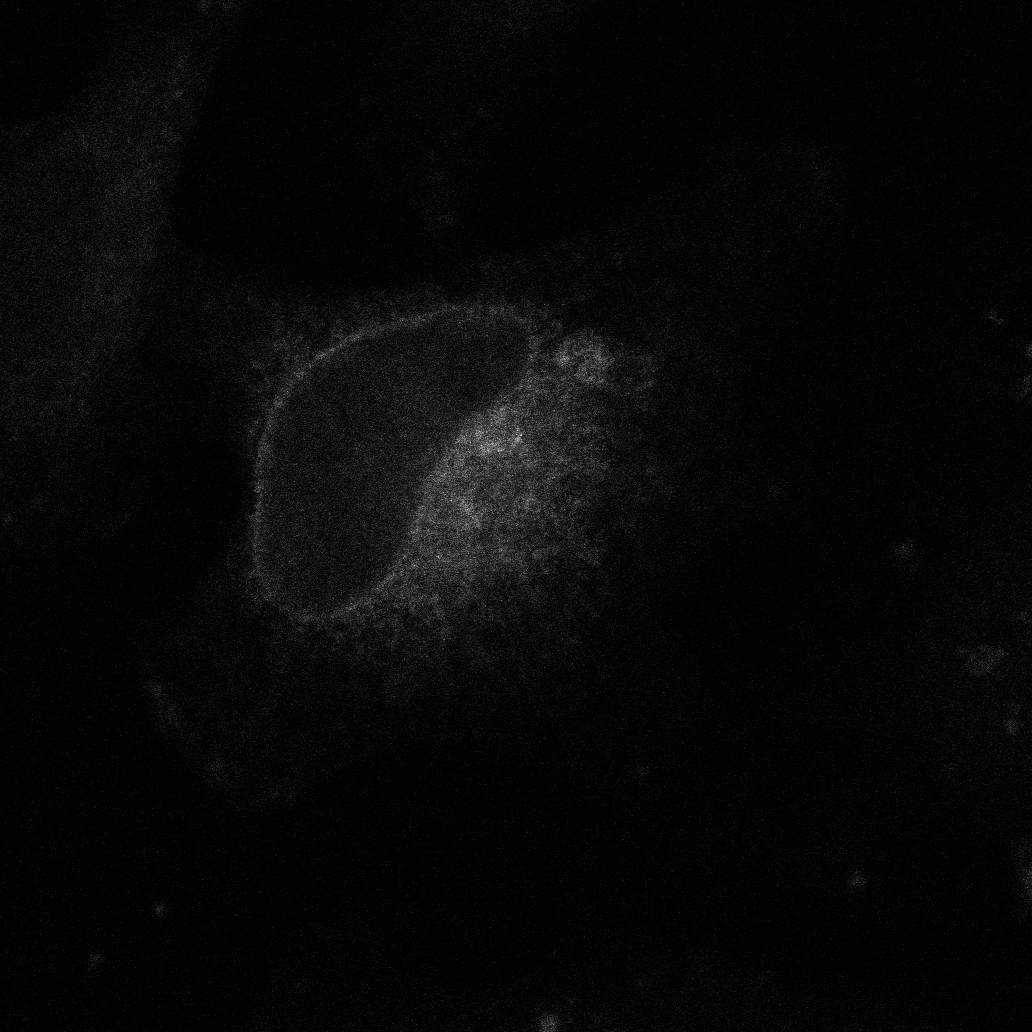

Supplement: Supplementary file 9 — Figure EV3 Source Data [file 44319_2025_423_MOESM9_ESM.zip › Figure EV3/3A/R19C_37-32.tif]

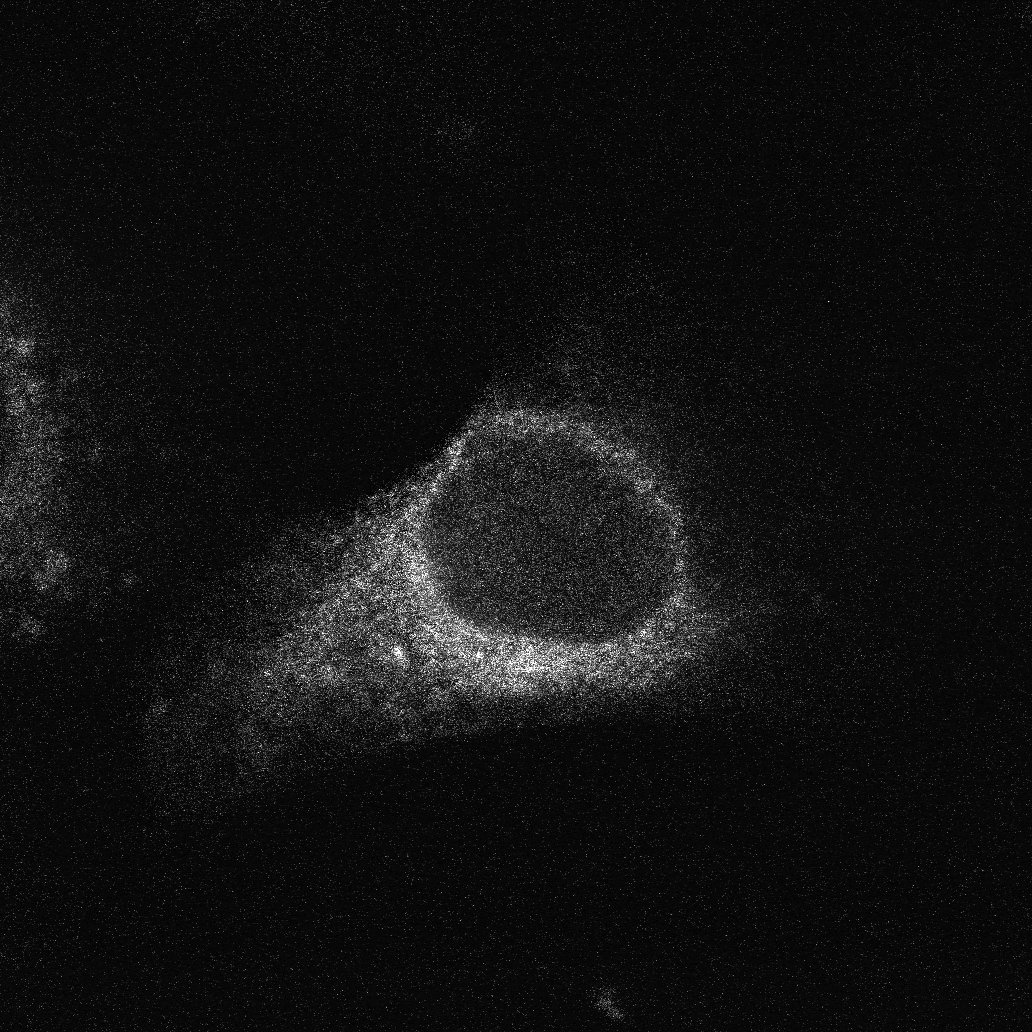

Supplement: Supplementary file 9 — Figure EV3 Source Data [file 44319_2025_423_MOESM9_ESM.zip › Figure EV3/3A/R19C_37.tif]

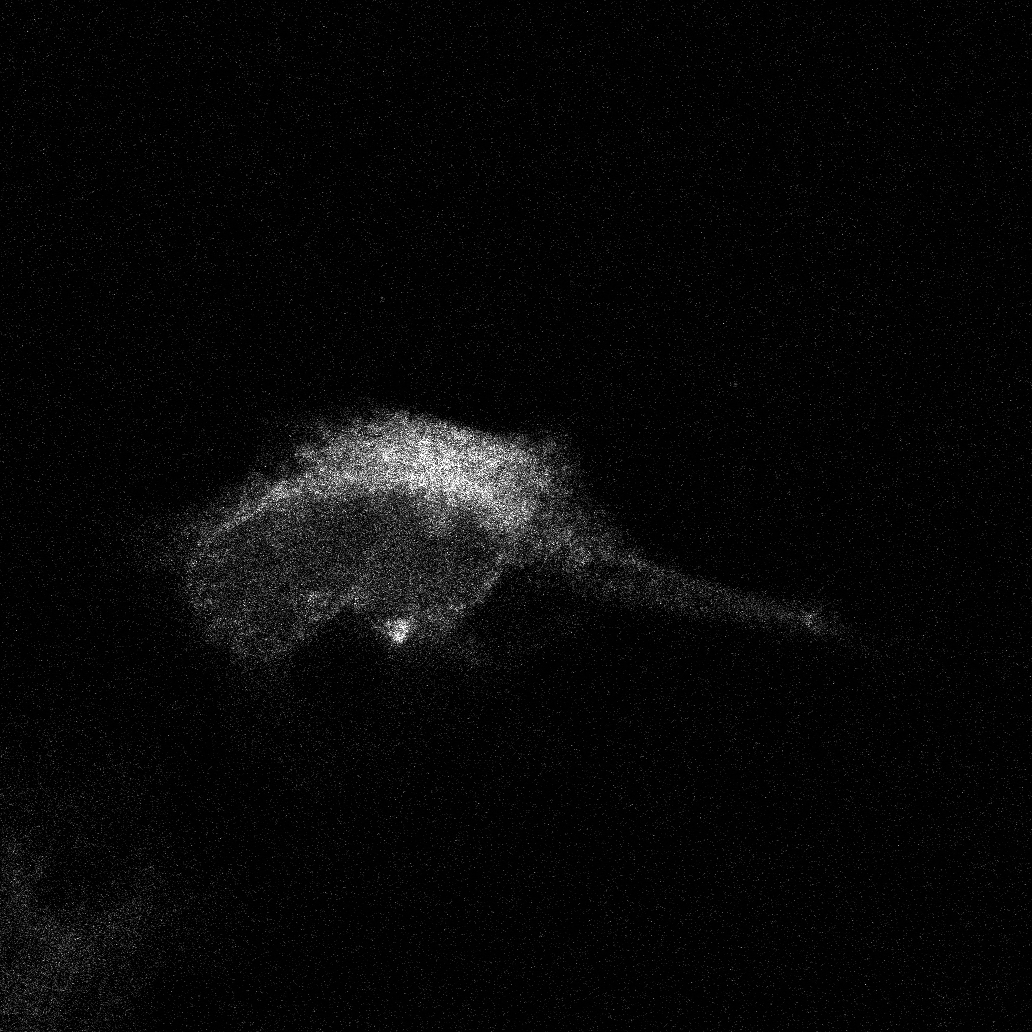

Supplement: Supplementary file 9 — Figure EV3 Source Data [file 44319_2025_423_MOESM9_ESM.zip › Figure EV3/3A/R99C_37-32-40.tif]

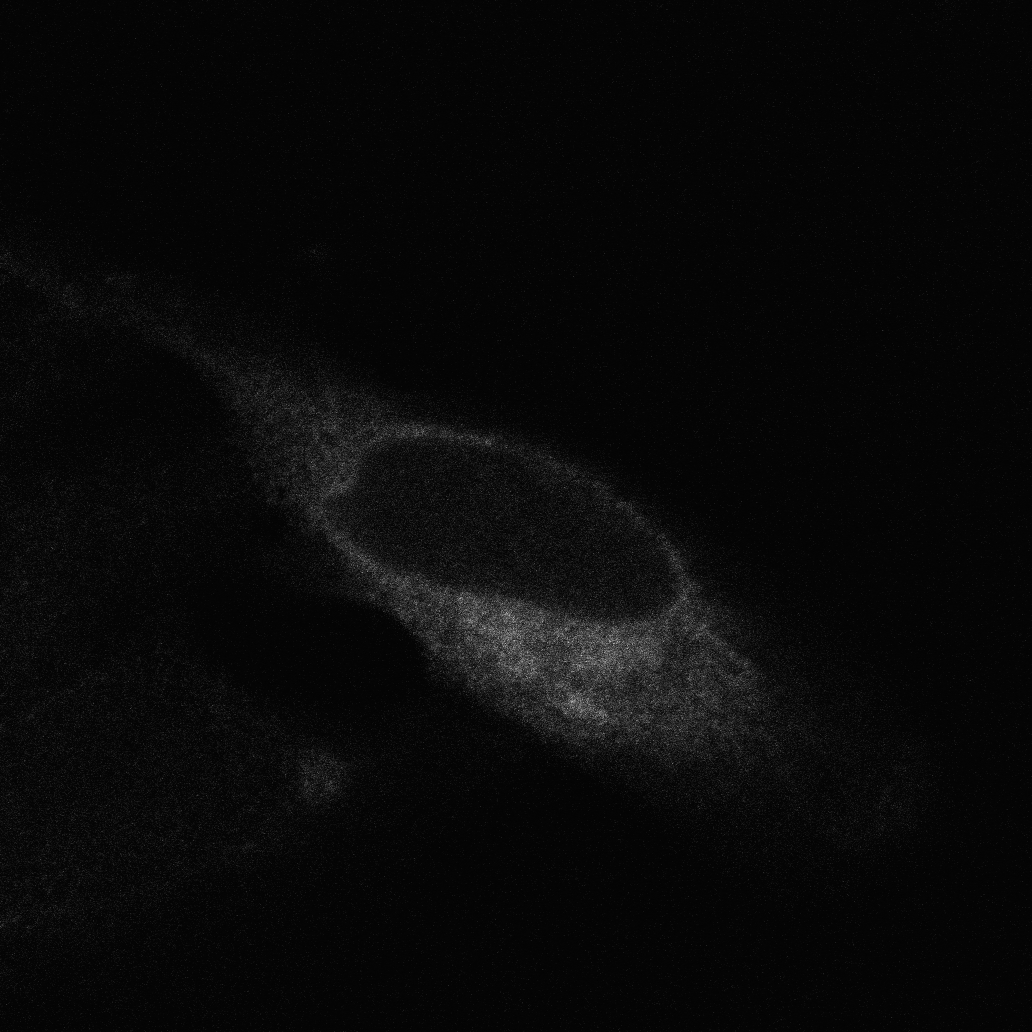

Supplement: Supplementary file 9 — Figure EV3 Source Data [file 44319_2025_423_MOESM9_ESM.zip › Figure EV3/3A/R99C_37-32.tif]

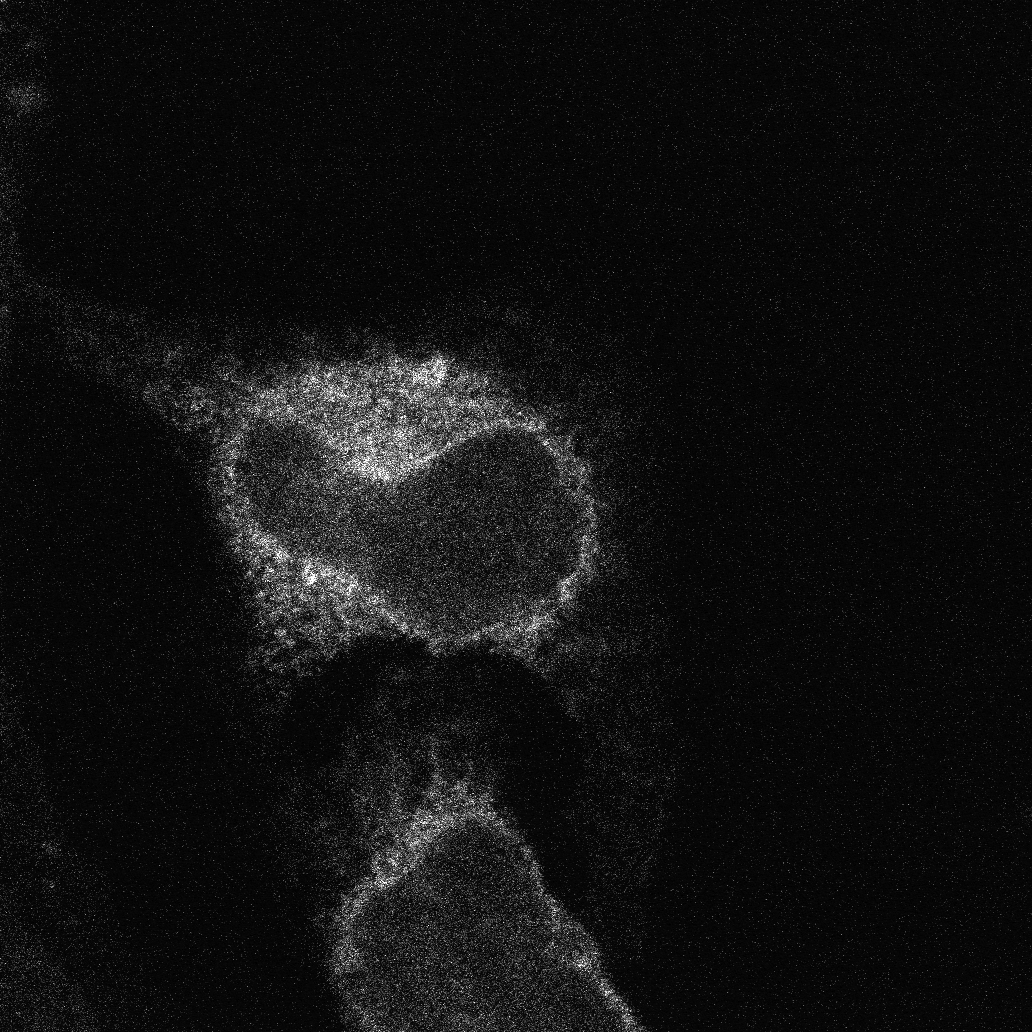

Supplement: Supplementary file 9 — Figure EV3 Source Data [file 44319_2025_423_MOESM9_ESM.zip › Figure EV3/3A/R99C_37.tif]

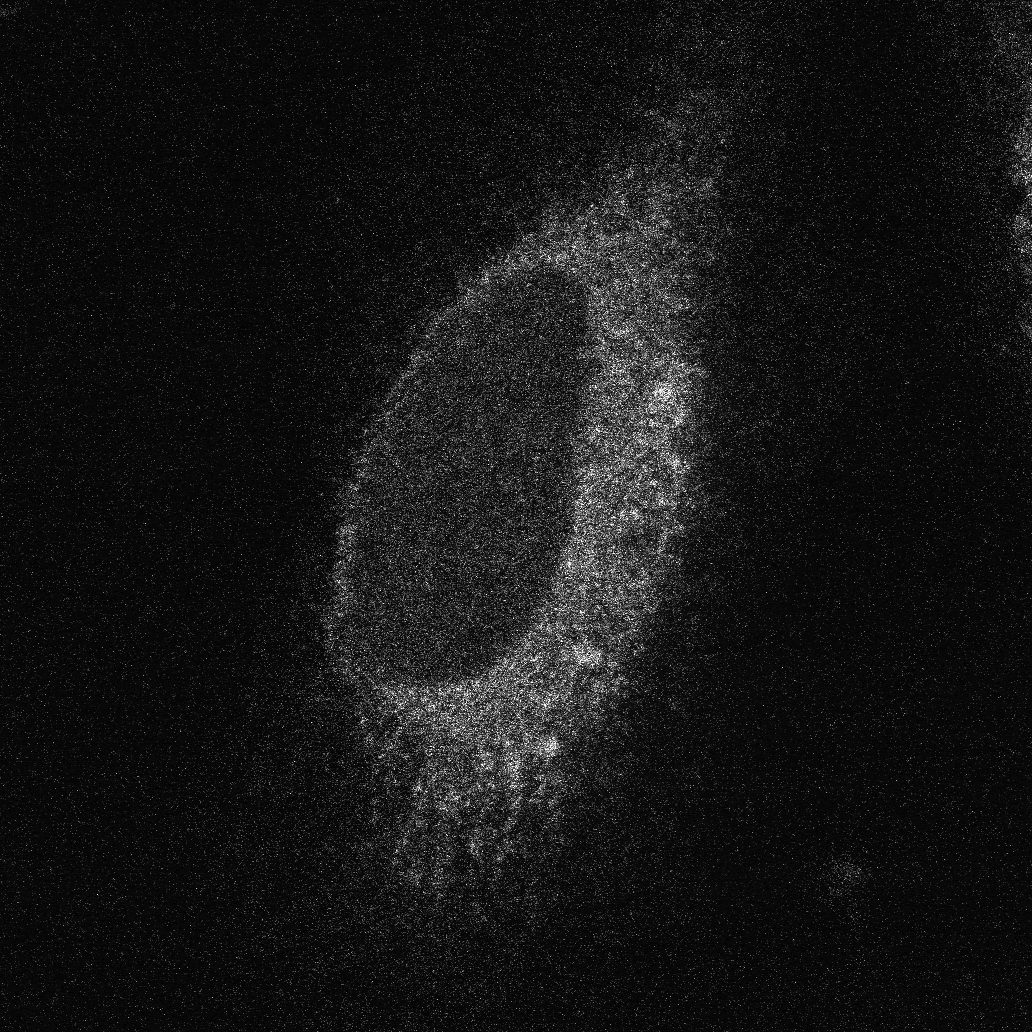

Supplement: Supplementary file 9 — Figure EV3 Source Data [file 44319_2025_423_MOESM9_ESM.zip › Figure EV3/3A/vector_37-32-40.tif]

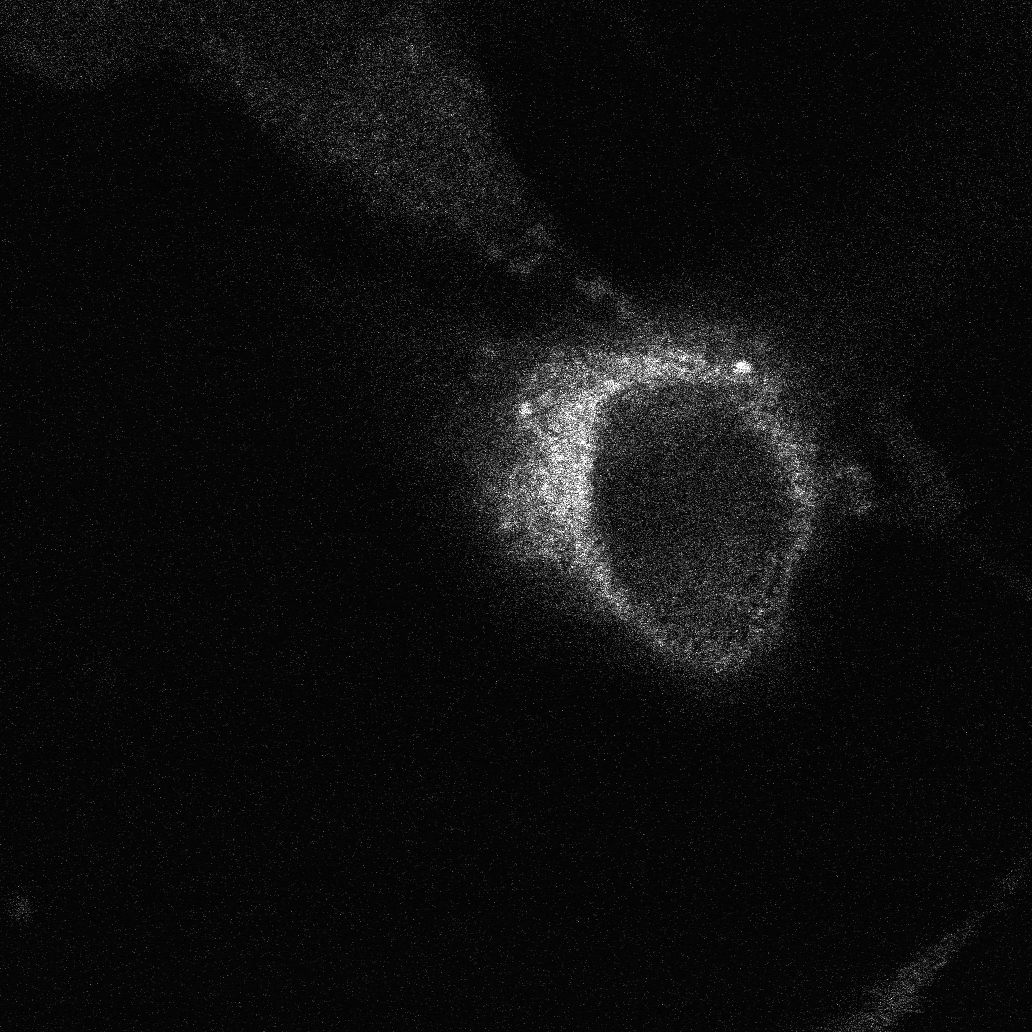

Supplement: Supplementary file 9 — Figure EV3 Source Data [file 44319_2025_423_MOESM9_ESM.zip › Figure EV3/3A/vector_37-32.tif]

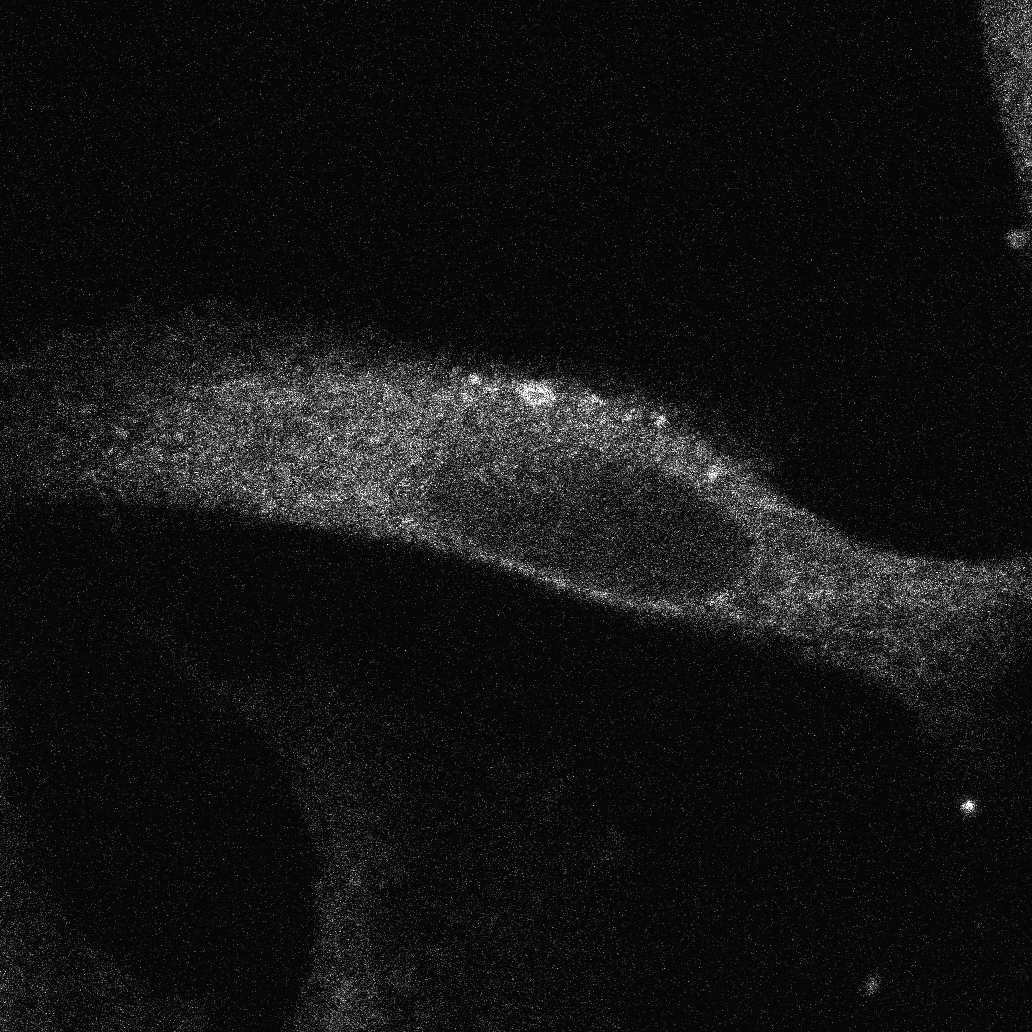

Supplement: Supplementary file 9 — Figure EV3 Source Data [file 44319_2025_423_MOESM9_ESM.zip › Figure EV3/3A/vector_37.tif]

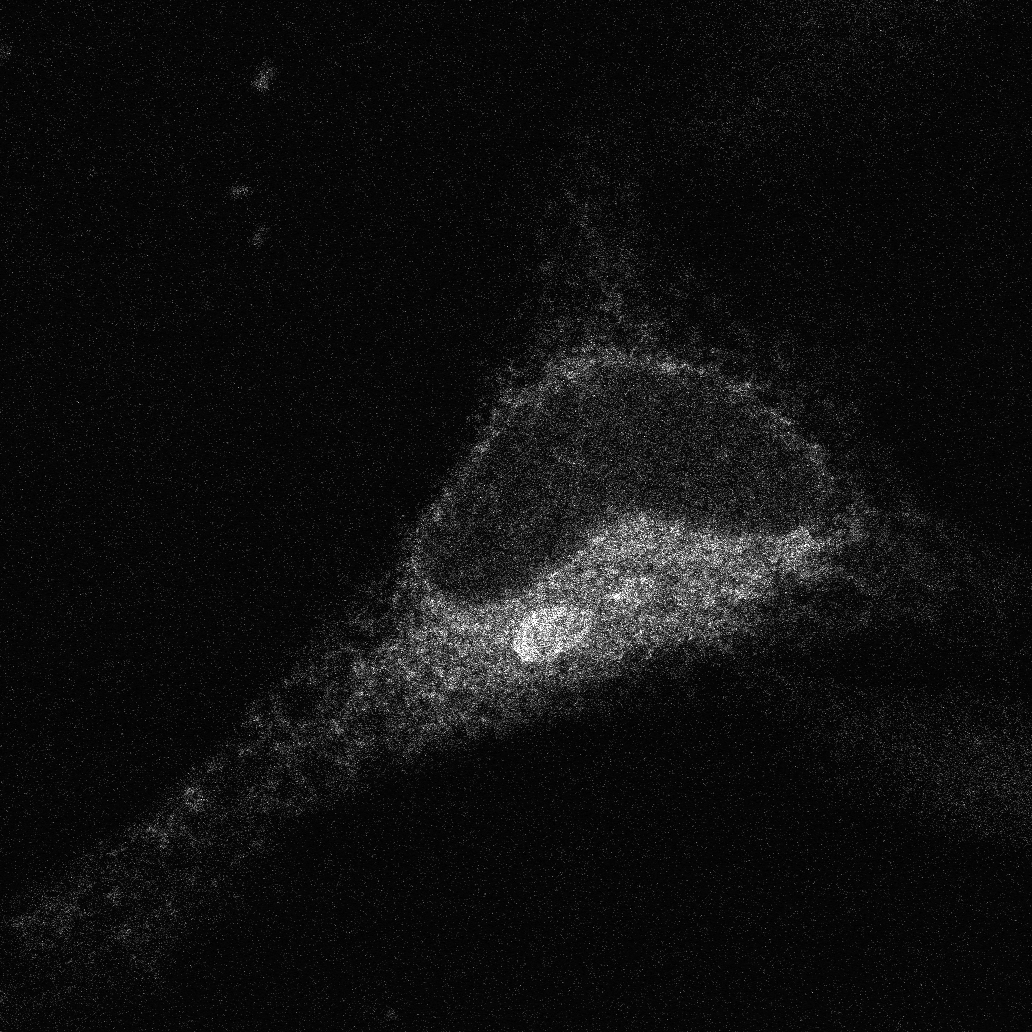

Supplement: Supplementary file 9 — Figure EV3 Source Data [file 44319_2025_423_MOESM9_ESM.zip › Figure EV3/3A/WT_37-32-40.tif]

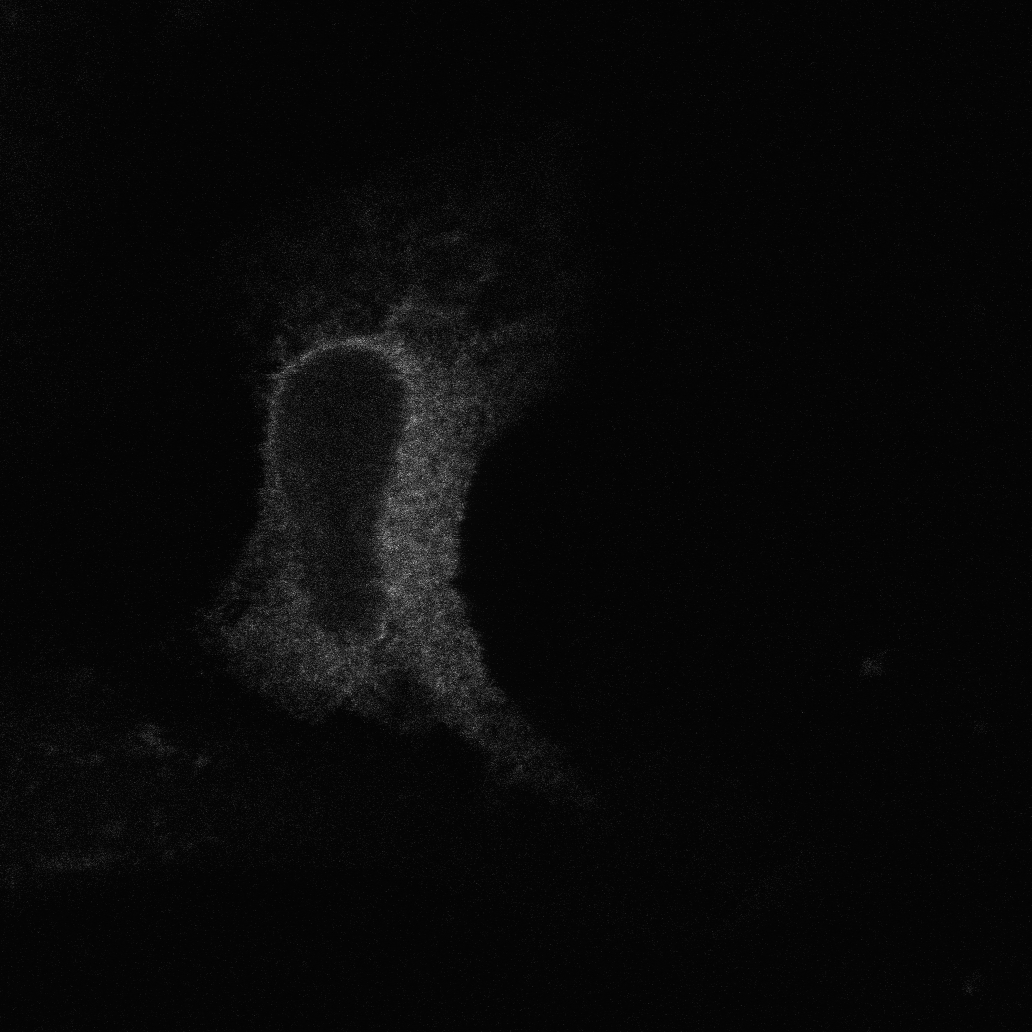

Supplement: Supplementary file 9 — Figure EV3 Source Data [file 44319_2025_423_MOESM9_ESM.zip › Figure EV3/3A/WT_37-32.tif]
