# Supplementary figures and images for: Distinct pathogenic mutations in ARF1 allow dissection of its dual role in cGAS-STING signalling (part 2 of 2)
Source: EMBO Rep. 2025 Mar 24;26(9):2232–61. doi: 10.1038/s44319-025-00423-7 (PMC7617634; doi:10.1038/s44319-025-00423-7)

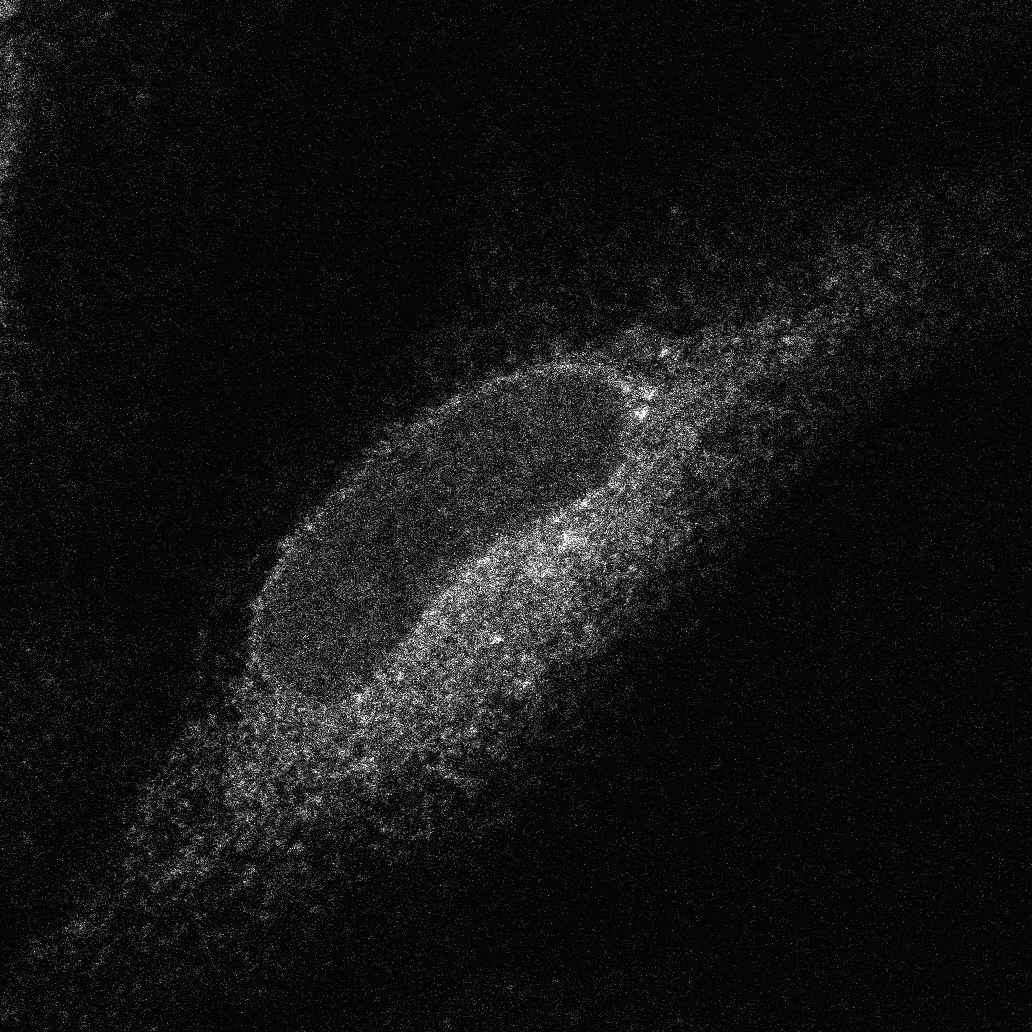

Supplement: Supplementary file 9 — Figure EV3 Source Data [file 44319_2025_423_MOESM9_ESM.zip › Figure EV3/3A/WT_37.tif]
