# Supplementary material for: Distinct pathogenic mutations in ARF1 allow dissection of its dual role in cGAS-STING signalling
Source: EMBO Rep. 2025 Mar 24;26(9):2232–61. doi: 10.1038/s44319-025-00423-7 (PMC7617634; doi:10.1038/s44319-025-00423-7)
Supplement: Supplementary file 10 — Expanded View Figures [file 44319_2025_423_MOESM10_ESM.pdf]

Expanded View Figures

Figure EV1. ARF1 expression and impact on mitochondrial integrity.

(**A**) Representative immunoblots of HEK293T cells transiently expressing increasing amounts of FLAG-tagged ARF1 WT, R19C or R99C, together with STING (+STING) or vector control (-STING. Blots were stained with anti-GAPDH. (**B**) Representative immunoblots of HEK293T cells transiently expressing increasing amounts (10–100 ng, in steps of 10 ng) of FLAG-tagged ARF1 WT, R19C or R99C, together with STING. Blots were stained with anti-GAPDH. (**C**) qPCR analysis of mitochondrial (mt) DNA (MT-ND-1) in the cytosolic fraction of Fig. 2B, normalised to total cellular mtDNA (MT-ND-1/KCNJ10). Bars represent the mean of $n = 6 \pm$ SEM (biological replicates). Statistical analysis was performed using two-tailed Student's t test with Welch's correction. *$p < 0.05$ ($p = 0.0184$ WT vs ABT/QVD); ns, not significant ($p = 0.0921$ WT vs R19C, $p = 0.0542$ WT vs R99C)). (**D**) Mitochondrial membrane potential (MMP) of HEK293T cell expressing ARF1 WT, R19C, R99C or vector control or treated with FCCP (10 µM/ 4 h). MMP was measured using flow cytometry and data depicted as the ratio of Mitotracker Red MFI (MMP) to Mitotracker Green MFI (mitochondrial mass), expressed as percentage of the vector control. Lines represent the mean of $n = 3 \pm$ SEM (biological replicates). Statistical analysis was performed using two-tailed Student's t test with Welch's correction. ***$p < 0.001$ ($p < 0.0001$ WT vs FCCP); ns, not significant ($p = 0.9464$ WT vs R19C). (**E**) Representative immunoblots of HEK293-STING cells transiently expressing increasing amounts of FLAG-tagged ARF1 WT, R19C, R99C or vector control. Blots were stained with anti-STING and anti-GAPDH.

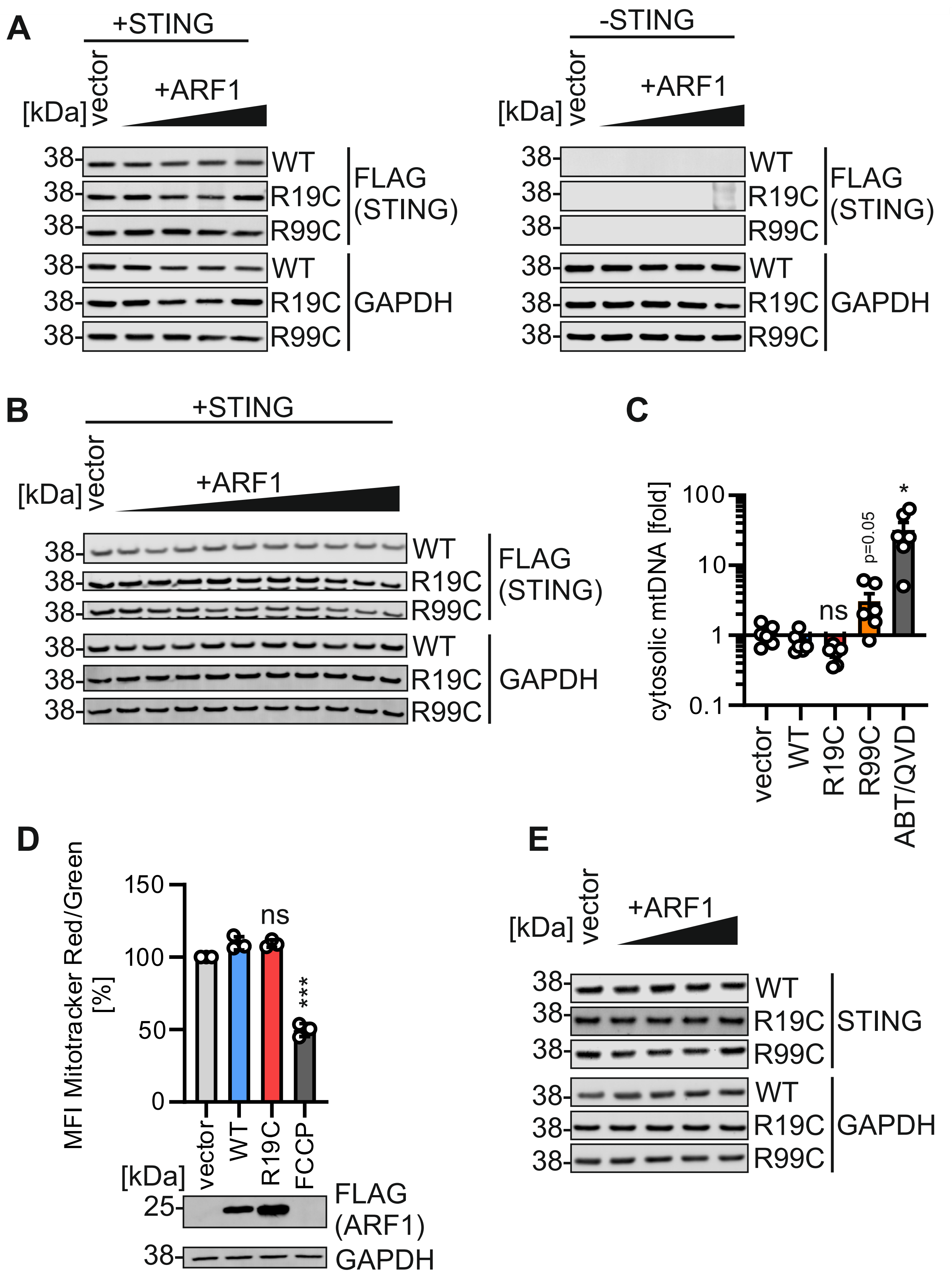
A
+STING
[kDa]
vector
+ARF1
38-
WT
R19C
R99C
FLAG (STING)
WT
R19C
R99C
GAPDH
-STING
B
+STING
C
cytosolic mtDNA [fold]
100
10
1
0.1
ns
p=0.05
*
vector
WT
R19C
R99C
ABT/QVD
D
MFI Mitotracker Red/Green [%]
150
100
50
0
ns
***
vector
WT
R19C
FCCP
25-
FLAG (ARF1)
GAPDH
E
STING

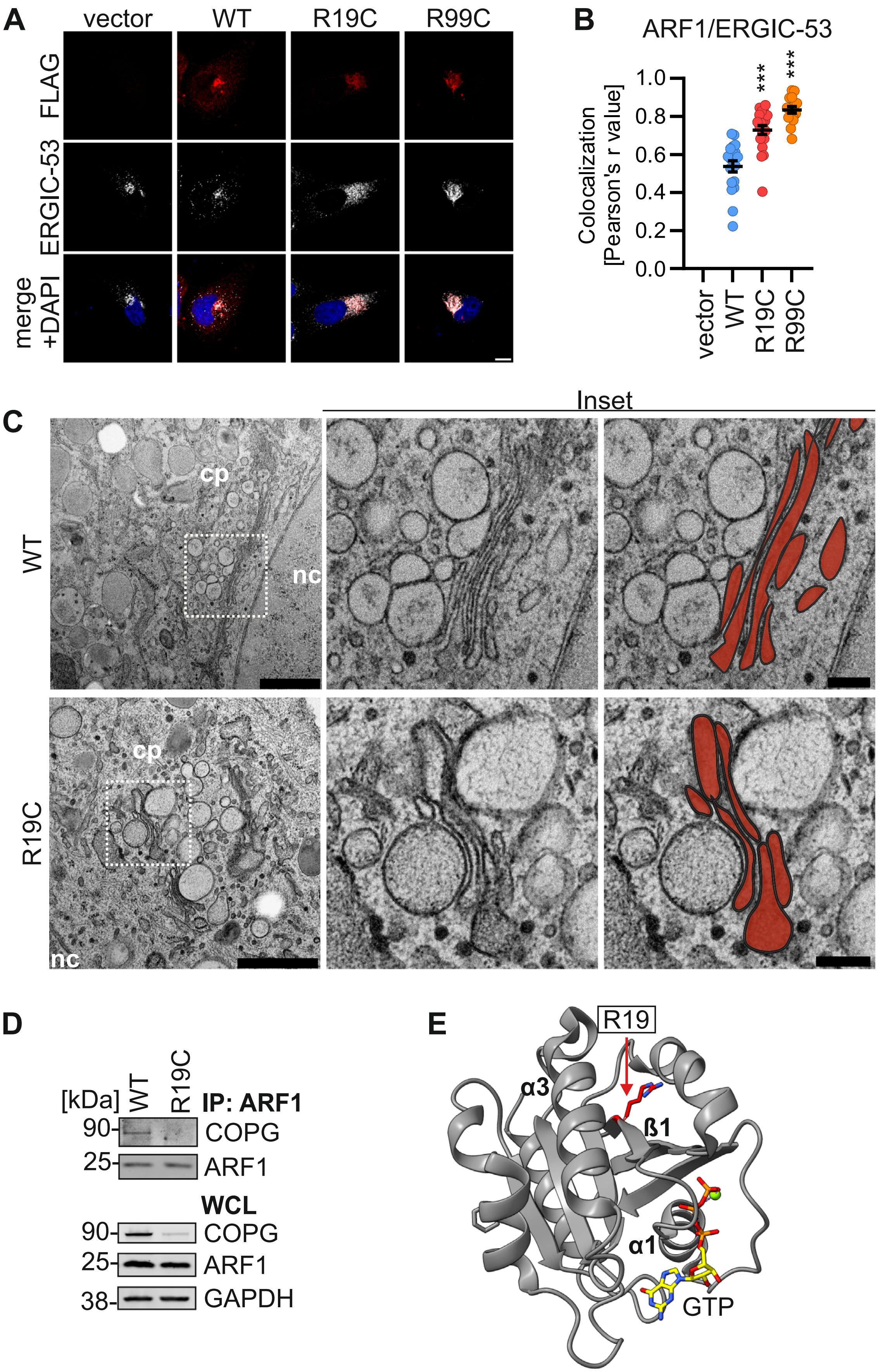
A
vector
WT
R19C
R99C
FLAG
ERGIC-53
merge +DAPI
B
ARF1/ERGIC-53
Colocalization [Pearson's r value]
1.0
0.8
0.6
0.4
0.2
0.0
***
***
vector
WT
R19C
R99C
C
Inset
WT
R19C
cp
nc
D
[kDa]
WT
R19C
IP: ARF1
90-
COPG
25-
ARF1
WCL
90-
COPG
25-
ARF1
38-
GAPDH
E
R19
α3
ß1
α1
GTP

Figure EV2. Localisation of ARF1 R19C and the impact on Golgi morphology and COPG interaction.

(**A**) Representative immunofluorescence images of Hela cells expressing ARF1 WT, R19C, R99C or vector control. 24 h post transfection the cells were stained with anti-FLAG (red) and anti- ERGIC-53 (white). Nuclei: DAPI (blue). Scale bar: 10 µm. (**B**) Colocalization (Pearson's correlation coefficient, r) between ARF1 and ERGIC-53 from the images shown in (**A**). Lines represent the mean of $n = 16–21 \pm$ SEM (individual cells). Statistical analysis was performed using two-tailed Student's t test with Welch's correction. ***$p < 0.001$ ($p < 0.0001$ WT vs R19C, WT vs R99C). (**C**) Representative transmission electron microscopy images of the Golgi area of primary fibroblasts from a healthy donor (WT) or patient AGS3238 (R19C). Stacked Golgi cisternae are highlighted in red. cp, cytoplasm. nc, nucleus. Scale bar: 1 µm (overview images), 200 nm (higher magnification image). (**D**) Immunoprecipitation (IP) of WCLs of primary fibroblasts from a healthy donor (WT) or patient AGS3238 (R19C) using anti-ARF1 coupled dynabeads. Immunoblots of the WCL and the IP were stained with anti-COPG, anti-ARF1 and anti-GAPDH. (**E**) Ribbon diagram of ARF1 in GTP-bound state (PDB: 2J59). R19 is highlighted in red, GTP in yellow. Source data are available online for this figure.

 © The Author(s)

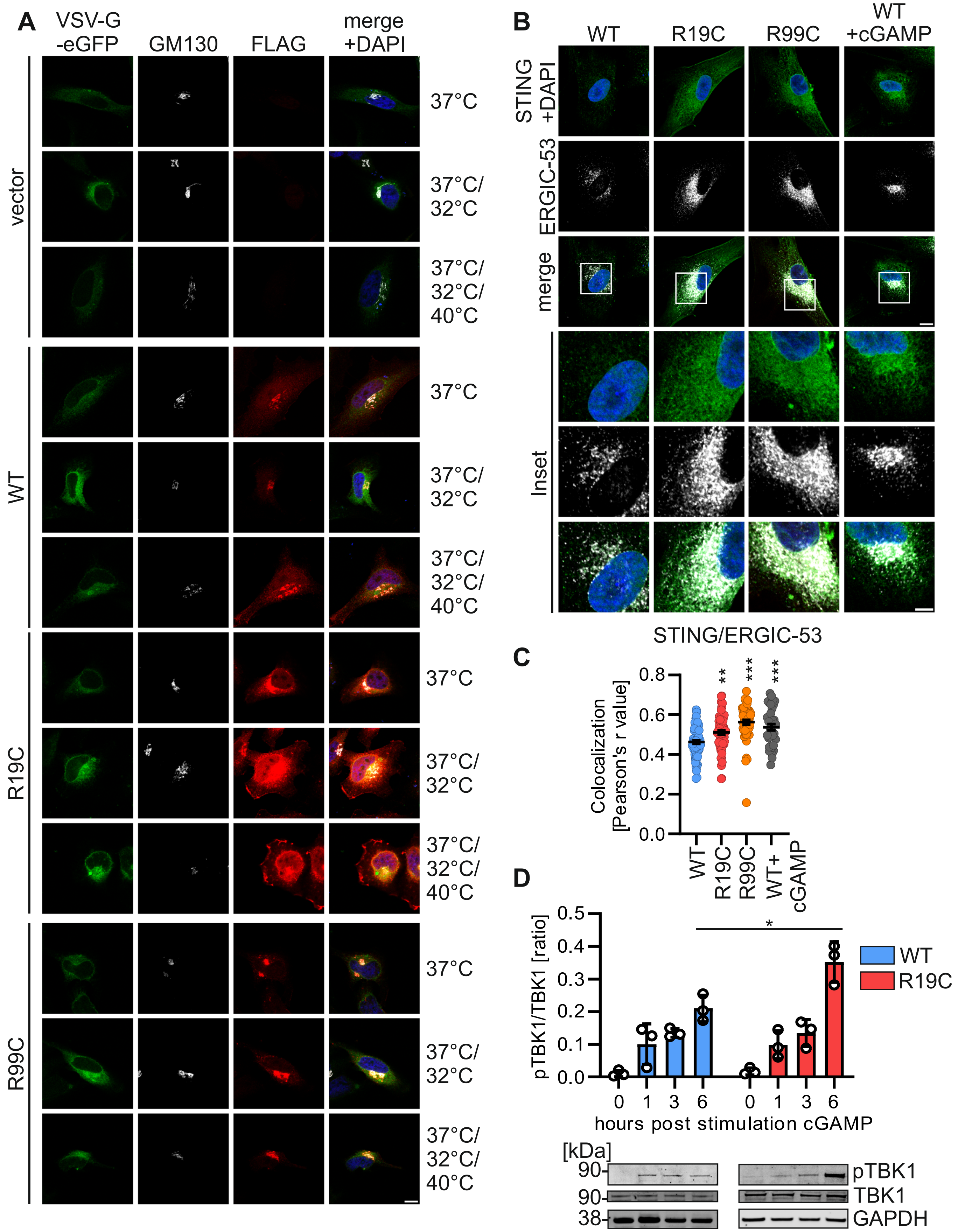
A
VSV-G -eGFP
GM130
FLAG
merge +DAPI
vector
WT
R19C
R99C
37°C
37°C/ 32°C
37°C/ 32°C/ 40°C
B
WT
R19C
R99C
WT +cGAMP
STING +DAPI
ERGIC-53
merge
Inset
C
STING/ERGIC-53
Colocalization [Pearson's r value]
0.0
0.2
0.4
0.6
0.8
**
***
***
WT
R19C
R99C
WT+ cGAMP
D
pTBK1/TBK1 [ratio]
0.0
0.1
0.2
0.3
0.4
0.5
*
WT
R19C
0 1 3 6 0 1 3 6
hours post stimulation cGAMP
[kDa]
90
90
38
pTBK1
TBK1
GAPDH

Figure EV3. ARF1 R19C impairs retrograde transport and accumulates STING at the ERGIC.

(**A**) Representative immunofluorescence images of Hela cells expressing ARF1 WT, R19C, R99C or vector control together with VSV-G-eGFP (green). 24 h post transfection the cells were subjected to the indicated temperature shifts (37 °C/32 °C/40 °C) and stained with anti-FLAG (red) and anti-GM130 (white). Nuclei: DAPI (blue). Scale bar: 10 µm. (**B**) Representative immunofluorescence images of primary fibroblasts of a healthy donor (WT), patient AGS3238 (R19C) or patient AGS460 (R99C). Healthy donor fibroblasts treated with cGAMP (20 µg/ml, 2 h) were used as positive control. Cells were stained with anti-STING (green) and anti-ERGIC-53 (white). Nuclei: DAPI (blue). Insets are shown in higher magnification. Scale bar: 10 µm (full size images), 5 µm (insets). (**C**) Colocalization (Pearson's correlation coefficient, r) between STING and ERGIC-53 from the images shown in (**B**). Lines represent the mean of $n = 33–77 \pm$ SEM (individual cells). Statistical analysis was performed using two-tailed Student's t test with Welch's correction. **$p < 0.01$ ($p = 0.0010$ WT vs R19C); ***$p < 0.001$ ($p < 0.0001$ WT vs R99C, WT vs cGAMP). (**D**) Quantification of the pTBK1 band intensities normalized to TBK1 band intensities in primary fibroblasts from a healthy donor (WT) or patient AGS3238 (R19C) at the indicated time points post stimulation with cGAMP (20 µg/ml). Bars represent mean of $n = 3 \pm$ SEM (biological replicates). Statistical analysis was performed using two-tailed Student's t test with Welch's correction. *$p < 0.05$ ($p = 0.0375$ WT vs R19C (6 h)). Blots were stained with anti-pTBK1, anti-TBK1 and anti-GAPDH. Source data are available online for this figure.

© The Author(s)

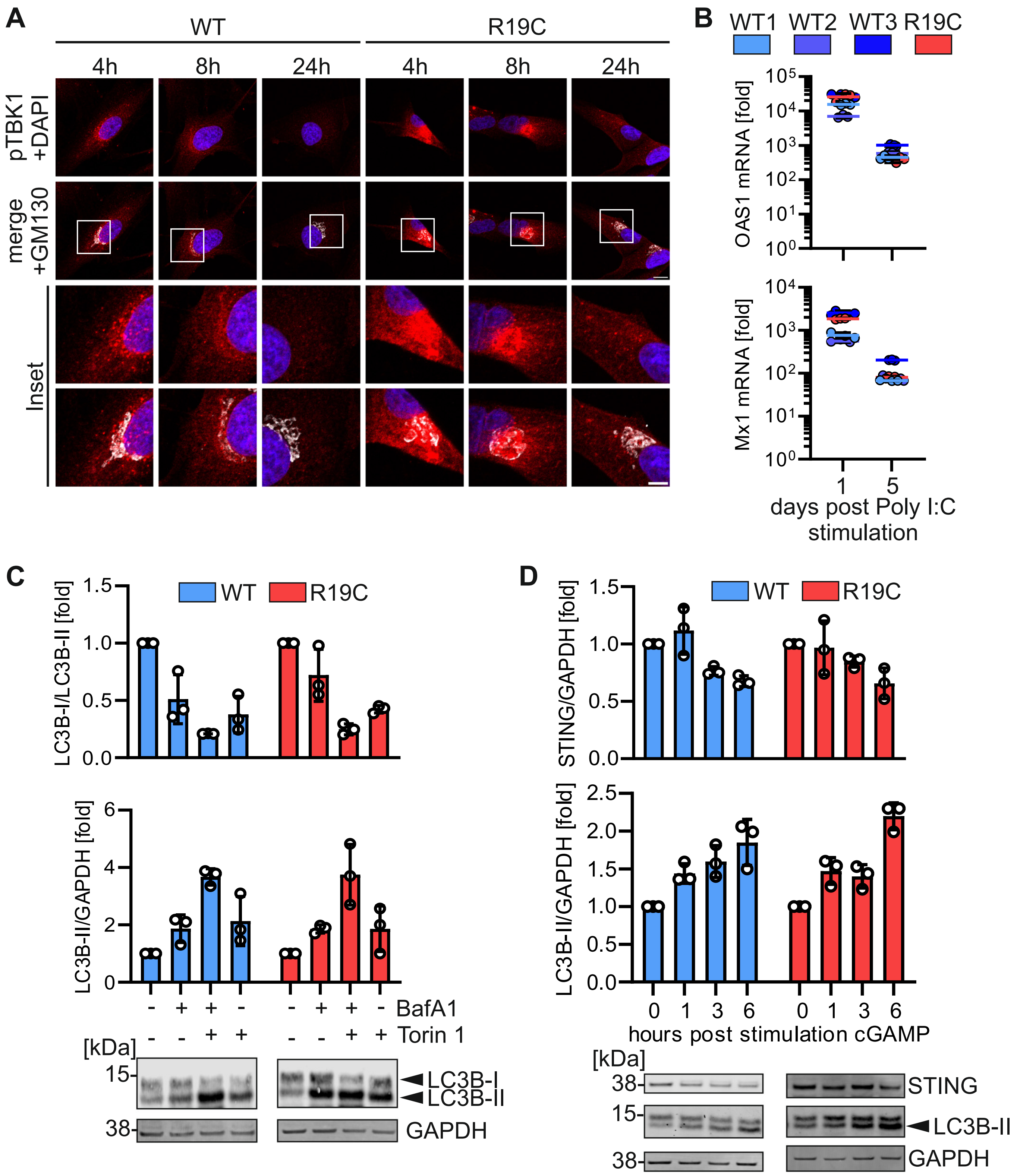
A
WT
R19C
4h
8h
24h
4h
8h
24h
pTBK1 +DAPI
merge +GM130
Inset
B
WT1 WT2 WT3 R19C
OAS1 mRNA [fold]
Mx1 mRNA [fold]
1
5
days post Poly I:C stimulation
C
WT
R19C
LC3B-I/LC3B-II [fold]
LC3B-II/GAPDH [fold]
BafA1
Torin 1
[kDa]
15
38
LC3B-I
LC3B-II
GAPDH
D
WT
R19C
STING/GAPDH [fold]
LC3B-II/GAPDH [fold]
0 1 3 6 0 1 3 6
hours post stimulation cGAMP
[kDa]
38
15
38
STING
LC3B-II
GAPDH

Figure EV4. Impact of cGAMP-stimulation on TBK1 activation and STING degradation.

(**A**) Representative immunofluorescence images of primary fibroblasts of a healthy donor (WT) or patient AGS3238 (R19C). Cells were treated with cGAMP (50 µg/ml) and stained at the indicated timepoints post stimulation with anti-pTBK1 (red) and anti-GM130 (white). Nuclei: DAPI (blue). Insets are shown in higher magnification. Scale bar: 10 µm (full size images), 5 µm (insets). (**B**) fold induction of OAS1 (top) and Mx1 (bottom) mRNA levels in primary fibroblasts from healthy donors (WT) or patient AGS3238 (R19C) as assessed by qPCR at indicated time points following stimulation with Poly I:C (1 µg/ml). Lines represent the mean of $n = 3 \pm$ SEM (biological replicates). (**C**) Quantification of the LC3B-I/LC3B-II (top) or LC3B-II/GAPDH (bottom) band intensities in primary fibroblasts from a healthy donor (WT) or patient AGS3238 (R19C) 4 h post treatment with bafilomycin A1 (BafA1, 250 nM), Torin 1 (1 µM) or both. Bars represent mean of $n = 3 \pm$ SEM (biological replicates). Blots were stained with anti-LC3B and anti-GAPDH. (**D**) Quantification of the STING (top) or LC3B-II (bottom) band intensities normalized to GAPDH band intensities in primary fibroblasts from a healthy donor (WT) or patient AGS3238 (R19C) at the indicated time points post stimulation with cGAMP (20 µg/ml). Bars represent mean of $n = 3 \pm$ SEM (biological replicates). Blots were stained with anti-STING, anti-LC3B and anti-GAPDH.

 © The Author(s)

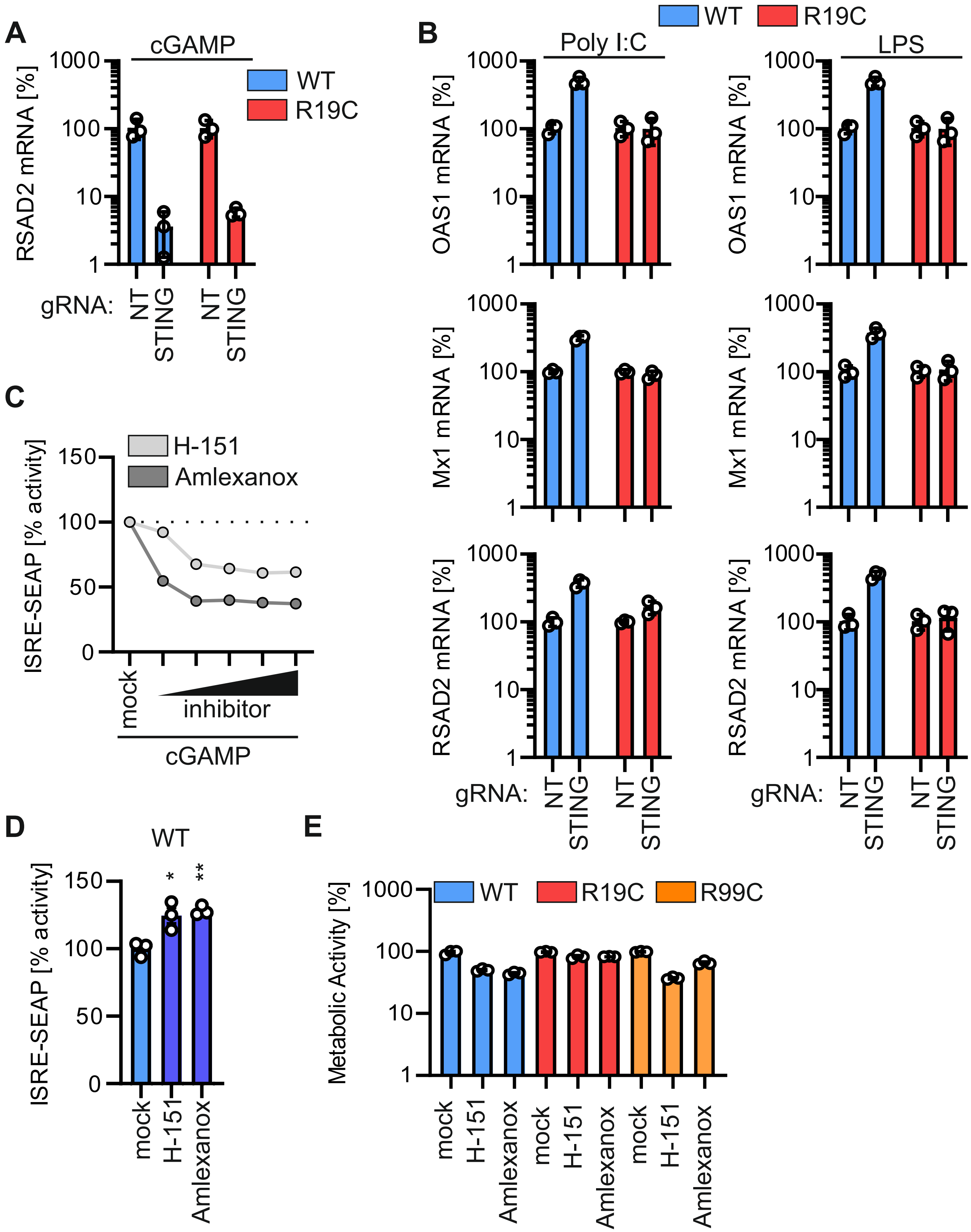
A
cGAMP
WT
R19C
RSAD2 mRNA [%]
1000
100
10
1
gRNA: NT STING NT STING
B
WT R19C
Poly I:C
LPS
OAS1 mRNA [%]
Mx1 mRNA [%]
RSAD2 mRNA [%]
gRNA: NT STING NT STING
C
H-151
Amlexanox
ISRE-SEAP [% activity]
150
100
50
0
mock
inhibitor
cGAMP
D
WT
ISRE-SEAP [% activity]
*
**
mock
H-151
Amlexanox
E
WT R19C R99C
Metabolic Activity [%]
mock H-151 Amlexanox mock H-151 Amlexanox mock H-151 Amlexanox

Figure EV5. Dysregulated type I IFN signalling is specific for the cGAS-STING pathway and treatment approaches.

(**A**) fold induction of RSAD2 mRNA levels in primary fibroblasts from a healthy donor (WT) or patient AGS3238 (R19C) as assessed by qPCR 16 h post stimulation with cGAMP (20 µg/ml). Treatment was performed 96 h post electroporation with Cas9/gRNA RNPs targeting either STING or non-targeting (NT). Lines represent the mean of $n = 3 \pm$ SEM (biological replicates). (**B**) fold induction of Oas1 (top), Mx1 (middle) and RSAD2 (bottom) mRNA levels in primary fibroblasts from a healthy donor (WT) or patient AGS3238 (R19C) as assessed by qPCR 16 h post stimulation with Poly I:C (1 µg/ml, left panels) or LPS (5 µg/ml, right panels). Treatment was performed 96 h post electroporation with Cas9-gRNA complexes targeting either STING or non-targeting (NT). Lines represent the mean of $n = 3 \pm$ SEM (biological replicates). (**C**) ISRE-SEAP activity of HEK293-STING cells stimulated with 20 µg/ml cGAMP (2 h prior to H 151, 1 h prior to amlexanox) and treated with increasing concentrations of H-151 (0.1 µM, 1 µM, 2 µM, 5 µM, 10 µM) or amlexanox (10 µg/ml, 20 µg/ml, 33 µg/ml, 40 µg/ml, 50 µg/ml). SEAP activity was quantified 16 h post treatment and normalized to cGAMP-stimulated control (mock). Dots present the mean of $n = 3 \pm$ SEM (biological replicates). (**D**) Supernatant transfer from primary fibroblasts of a healthy donor (WT) consecutively treated four times every 48 h with H-151 (2 µM) or amlexanox (33 µg/ml) to HEK293-STING cells (293-Dual-hSTING-R232). ISRE-SEAP activity was quantified 48 h post transfer and normalised to metabolic activity. Lines represent the mean of $n = 3 \pm$ SEM (biological replicates). Statistical analysis was performed using two-tailed Student's t test with Welch's correction. $^*p < 0.05$ ($p = 0.0361$ mock vs H-151); $^{**}p < 0.01$ ($p = 0.0028$ mock vs amlexanox). (**E**) Cell viability of the primary fibroblasts from Figs. 5G, H and EV3D prior to supernatant transfer as assessed by MTT assay and normalized to the respective mock control. Lines represent the mean of $n = 3 \pm$ SEM (biological replicates).

 © The Author(s)
