# Supplementary material for: Distinct pathogenic mutations in ARF1 allow dissection of its dual role in cGAS-STING signalling
Source: EMBO Rep. Author manuscript; Available in PMC 2025 Apr 30. (PMC7617634; doi:10.1038/s44319-025-00423-7)
Supplement: Supplementary file 1 [file EMS204466-supplement-Supplementary_figures.pdf]

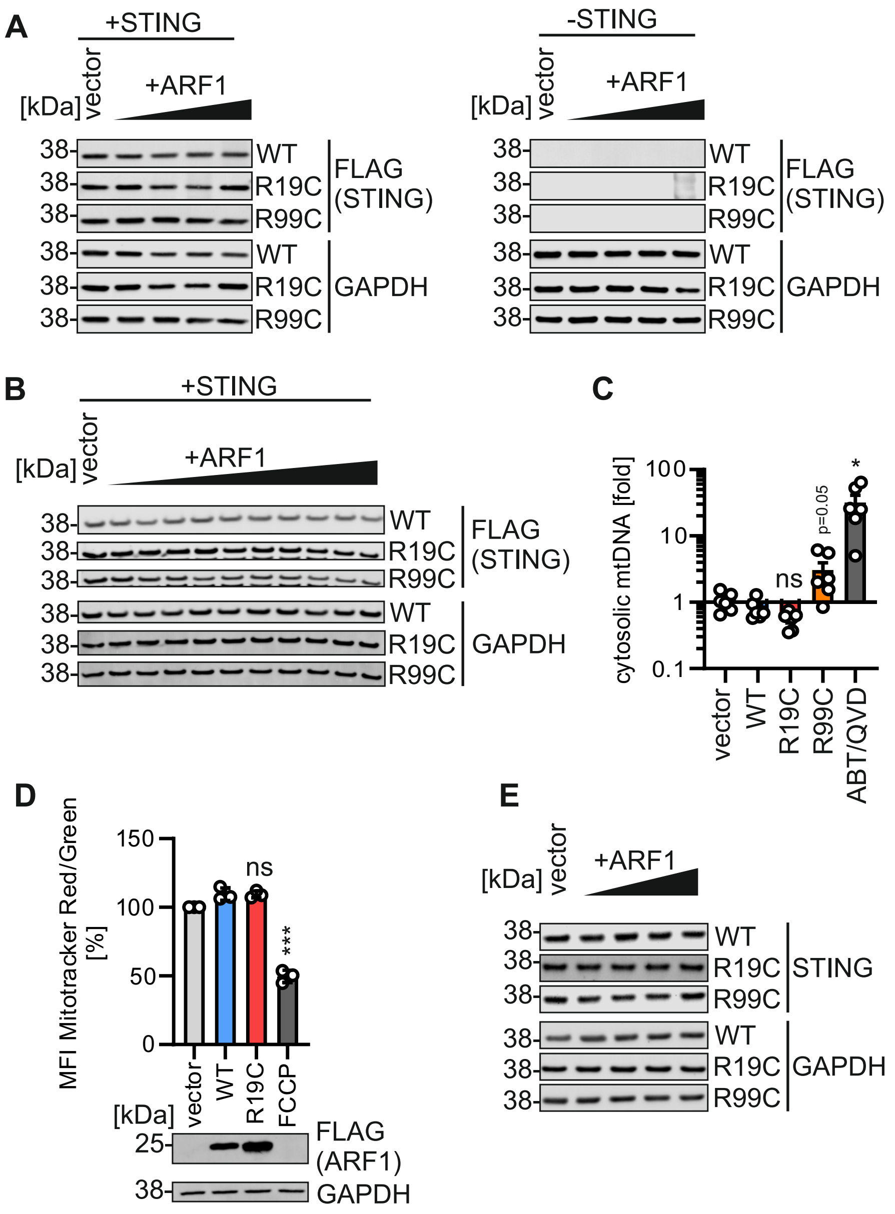
A
+STING
-STING
vector
+ARF1
[kDa]
38
WT
R19C
R99C
FLAG (STING)
GAPDH
B
+STING
vector
+ARF1
[kDa]
38
WT
R19C
R99C
FLAG (STING)
GAPDH
C
cytosolic mtDNA [fold]
100
10
1
0.1
ns
p=0.05
*
vector
WT
R19C
R99C
ABT/QVD
D
MFI Mitotracker Red/Green [%]
150
100
50
0
ns
***
vector
WT
R19C
FCCP
[kDa]
25
38
FLAG (ARF1)
GAPDH
E
vector
+ARF1
[kDa]
38
WT
R19C
R99C
STING
GAPDH

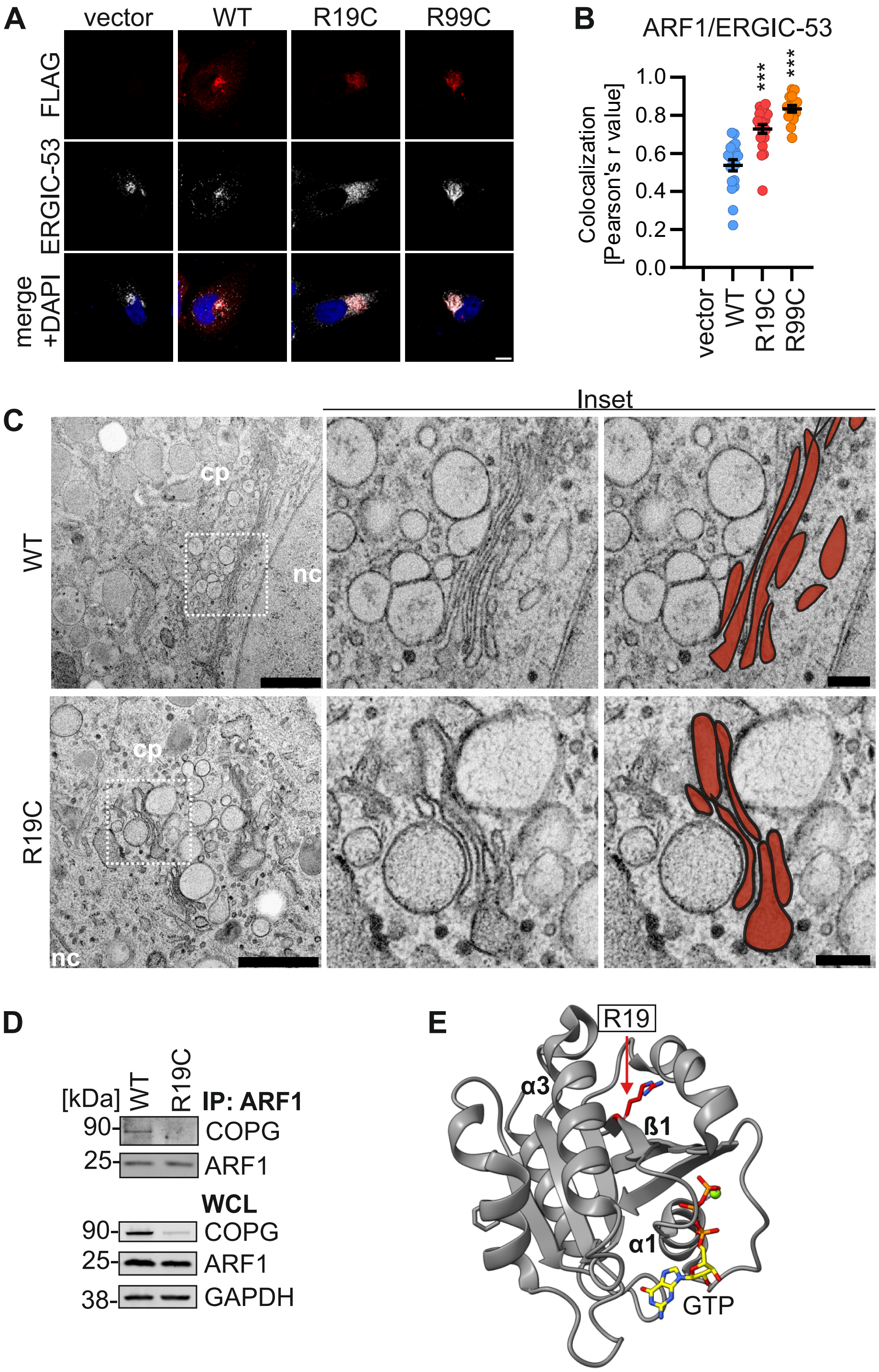
A
vector
WT
R19C
R99C
FLAG
ERGIC-53
merge +DAPI
B
ARF1/ERGIC-53
Colocalization [Pearson's r value]
1.0
0.8
0.6
0.4
0.2
0.0
***
***
vector
WT
R19C
R99C
C
Inset
WT
R19C
cp
nc
D
[kDa]
WT
R19C
IP: ARF1
90-
COPG
25-
ARF1
WCL
90-
COPG
25-
ARF1
38-
GAPDH
E
R19
α3
ß1
α1
GTP

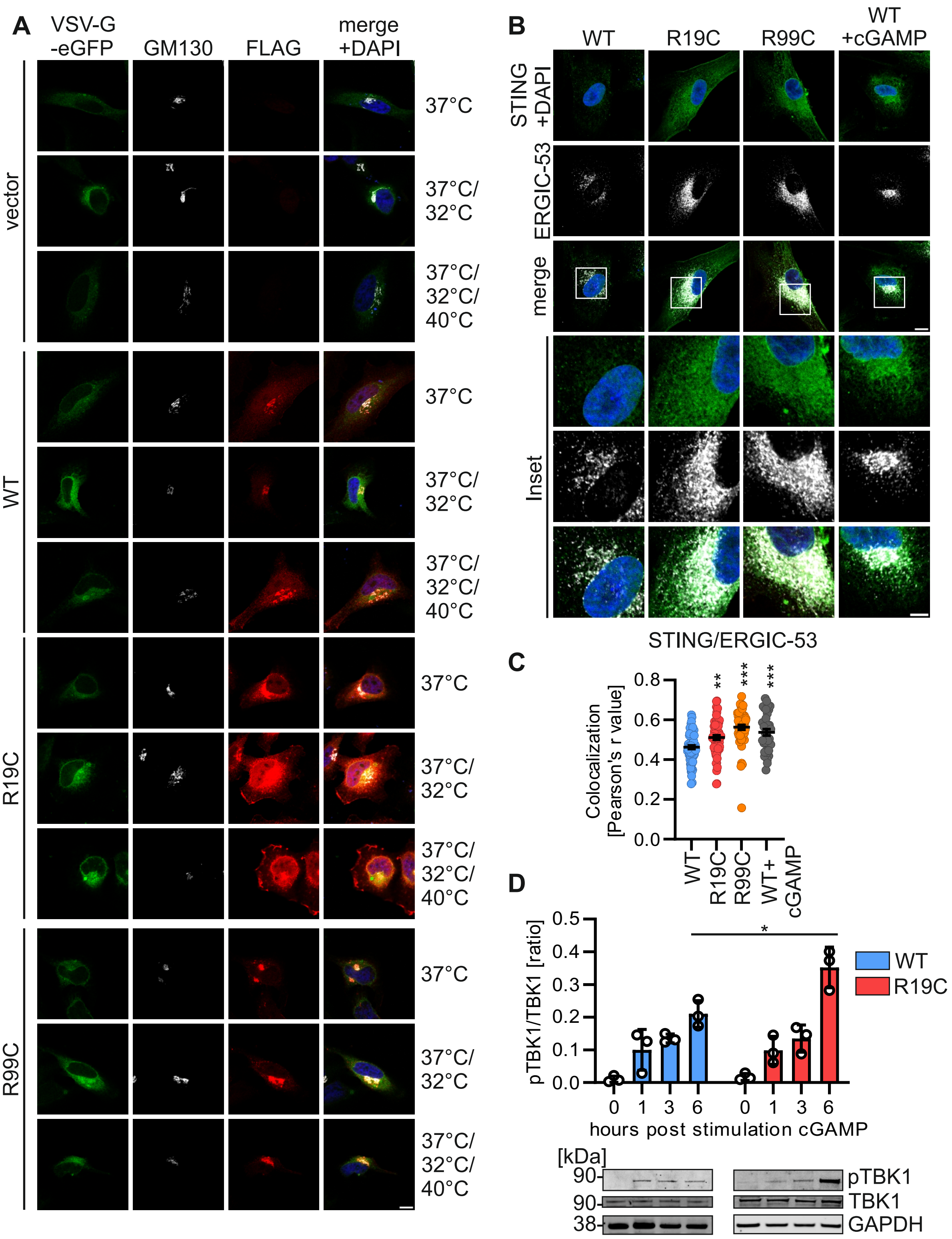
A
VSV-G -eGFP
GM130
FLAG
merge +DAPI
vector
WT
R19C
R99C
37°C
37°C/ 32°C
37°C/ 32°C/ 40°C
B
WT
R19C
R99C
WT +cGAMP
STING +DAPI
ERGIC-53
merge
Inset
C
STING/ERGIC-53
Colocalization [Pearson's r value]
0.0
0.2
0.4
0.6
0.8
**
***
***
WT
R19C
R99C
WT+ cGAMP
D
pTBK1/TBK1 [ratio]
0.0
0.1
0.2
0.3
0.4
0.5
*
WT
R19C
0
1
3
6
hours post stimulation cGAMP
[kDa]
90
90
38
pTBK1
TBK1
GAPDH

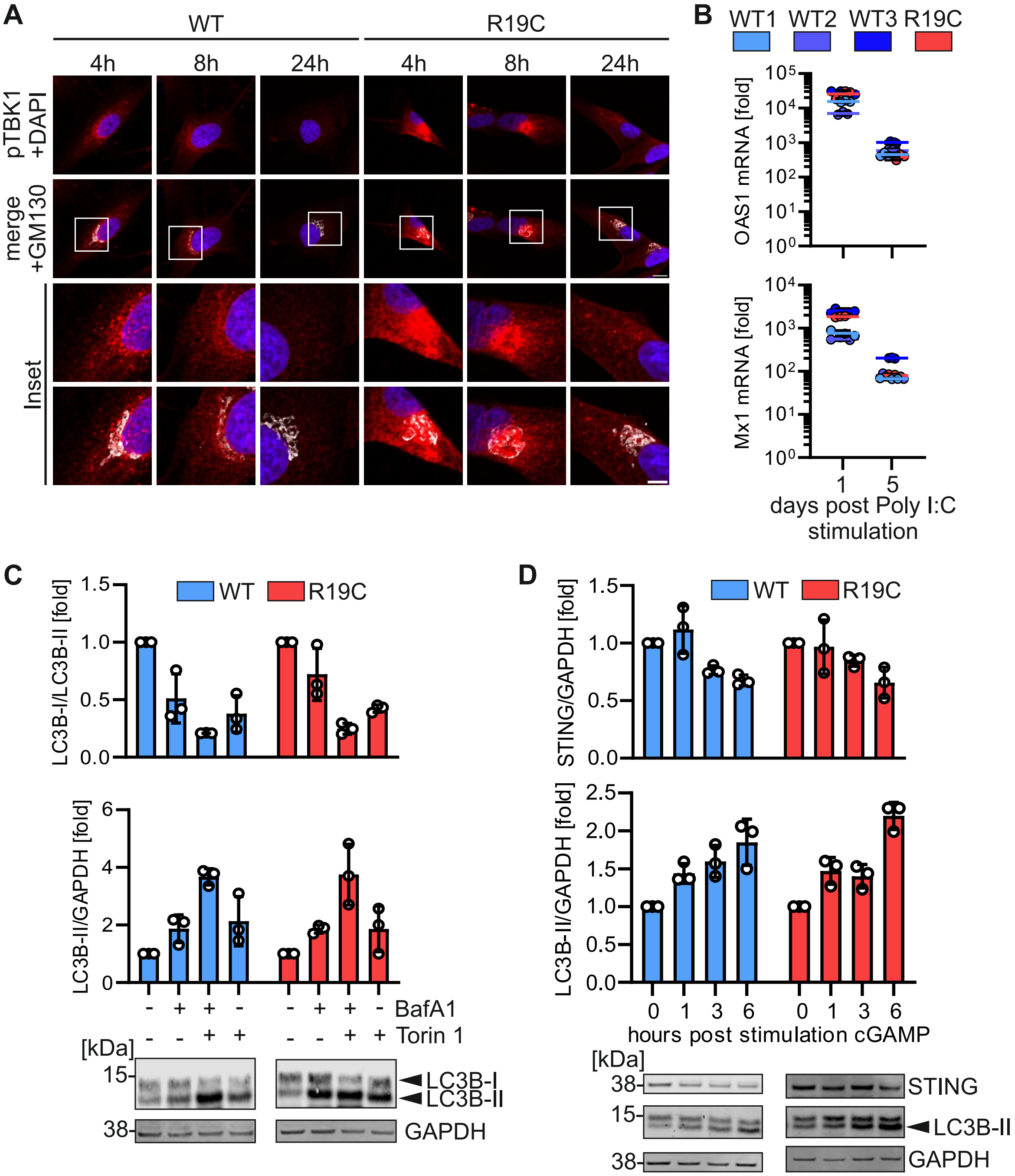
A
WT
R19C
4h
8h
24h
pTBK1 +DAPI
merge +GM130
Inset
B
WT1
WT2
WT3
R19C
OAS1 mRNA [fold]
Mx1 mRNA [fold]
1
5
days post Poly I:C stimulation
C
WT
R19C
LC3B-I/LC3B-II [fold]
LC3B-II/GAPDH [fold]
BafA1
Torin 1
[kDa]
15
38
LC3B-I
LC3B-II
GAPDH
D
WT
R19C
STING/GAPDH [fold]
LC3B-II/GAPDH [fold]
0
1
3
6
hours post stimulation cGAMP
[kDa]
38
15
STING
LC3B-II
GAPDH

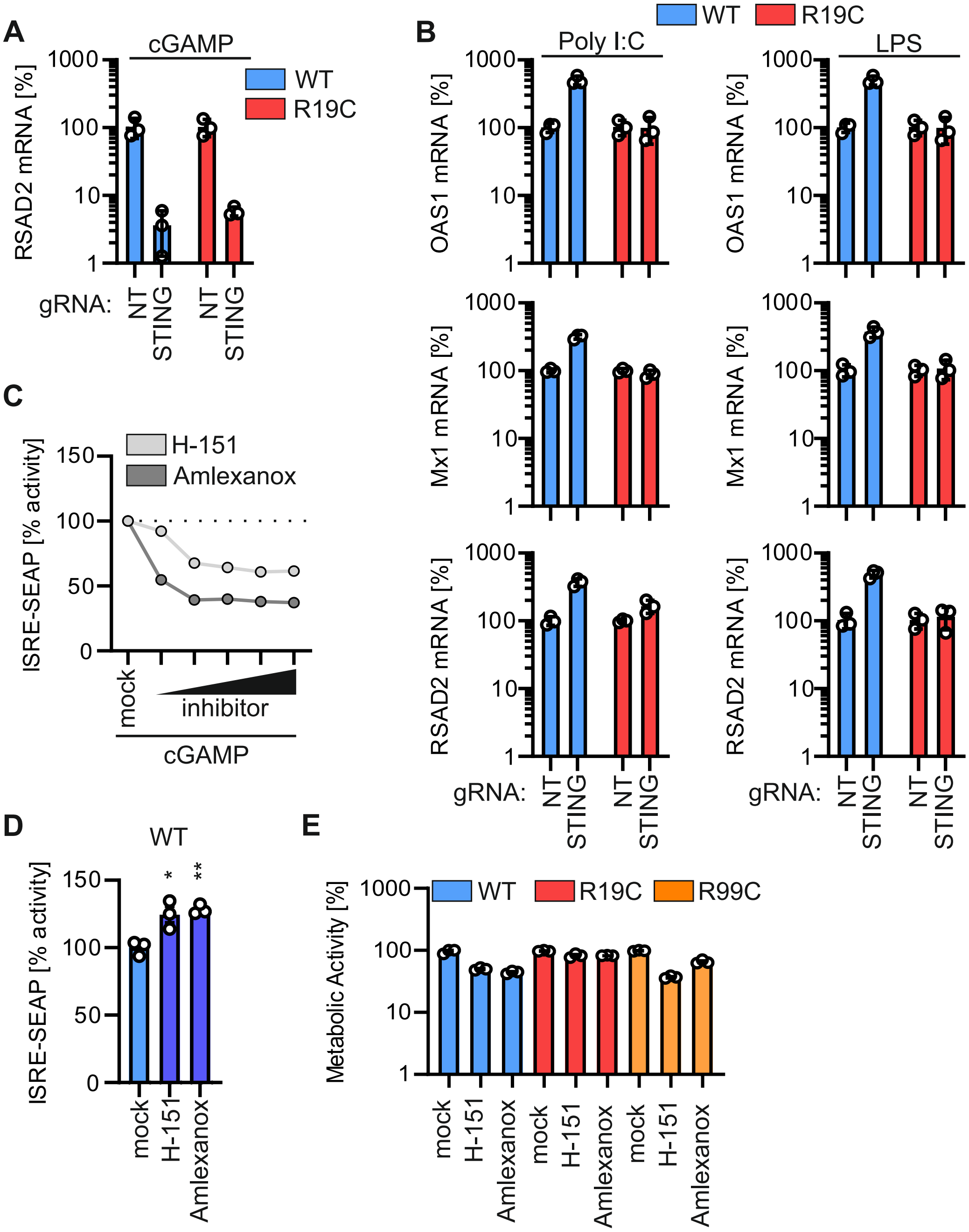
A
cGAMP
RSAD2 mRNA [%]
1000
100
10
1
WT
R19C
gRNA:
NT
STING
NT
STING
B
WT
R19C
Poly I:C
LPS
OAS1 mRNA [%]
Mx1 mRNA [%]
RSAD2 mRNA [%]
1000
100
10
1
gRNA:
NT
STING
NT
STING
C
ISRE-SEAP [% activity]
150
100
50
0
H-151
Amlexanox
mock
inhibitor
cGAMP
D
WT
ISRE-SEAP [% activity]
150
100
50
0
*
**
mock
H-151
Amlexanox
E
Metabolic Activity [%]
1000
100
10
1
WT
R19C
R99C
mock
H-151
Amlexanox
mock
H-151
Amlexanox
mock
H-151
Amlexanox
